# Positional interpretation of cis-regulatory code and nucleosome organization with deep learning models

**DOI:** 10.1038/s41467-026-74807-1

**Published:** 2026-07-04

**Authors:** Charles E. McAnany, Melanie Weilert, Grishma Mehta, Fahad Kamulegeya, Jennifer M. Gardner, Jacob Schreiber, Anshul Kundaje, Julia Zeitlinger

**Affiliations:** 1 Stowers Institute for Medical Research, Kansas City, MO USA; 2 Indian Institute of Science Education & Research, Pune, India; 3 Research Institute of Molecular Pathology, Vienna BioCenter, Vienna, Austria; 4 Department of Genomics and Computational Biology, UMass Chan Medical School, Worcester, MA USA; 5 Department of Genetics, Stanford University, Palo Alto, CA USA; 6 Department of Computer Science, Stanford University, Palo Alto, CA USA; 7 Department of Pathology & Laboratory Medicine, The University of Kansas Medical Center, Kansas City, KS USA

**Keywords:** Chromatin remodelling, Epigenomics, Transcriptional regulatory elements, Chromatin analysis

## Abstract

Sequence-to-function neural networks learn cis-regulatory sequence rules driving many types of genomic data. Interpreting these models to relate the sequence rules to underlying biological processes remains challenging, especially for complex genomic readouts such as MNase-seq, which maps nucleosome occupancy but is confounded by experimental bias. Here, we introduce pairwise influence by sequence attribution (PISA), which uses attribution to combinatorially decode which bases contributed to the readout at a specific genomic coordinate. PISA visualizes the effects of transcription factor motifs, detects undiscovered motifs with complex contribution patterns, and reveals experimental biases. By learning the bias for MNase-seq, PISA enables unprecedented nucleosome prediction models. These models allow the de novo discovery of nucleosome-positioning motifs and reveal the basis of Micro-C chromatin domain boundaries through systematic motif perturbations. Finally, these models allow the design of sequences with altered nucleosome configurations. These results show that PISA is a versatile tool that expands our ability to train and interpret sequence-to-function neural networks on genomics data and understand the underlying cis-regulatory code.

## Abstract (web summary)

Cis-regulatory sequence models capture genomic function, but interpreting their underlying rules is challenging. Here, the authors develop PISA, an attribution-based framework that decodes sequence contributions, revealing regulatory motifs, correcting experimental biases, and enabling prediction and design of nucleosome positioning.

## Introduction

Deciphering the cis-regulatory code of gene regulation in non-coding genomic sequences is one of the remaining grand challenges in biology. A complete understanding would allow us to read the gene regulatory information in the human genome, identify genetic variants involved in disease, and design synthetic regulatory sequences for therapeutic and industrial purposes^1^. A key breakthrough to learning these cis-regulatory sequence rules is sequence-to-function neural networks^1^. These models take DNA sequence as input and are trained to predict the readout of genomics assays that measure various aspects of gene regulation, including transcription factor (TF) binding, chromatin accessibility, nucleosome organization, transcription initiation, and gene expression^2–12^.

An example is the BPNet family of models, convolutional neural networks that predict the profile of genomics data at base resolution, in addition to predicting the total read counts per region^3^. BPNet was originally designed to learn high-resolution TF binding data^3,13,14^, but the sequence-to-profile predictions and the lightweight architecture make it a robust and versatile framework for many data types, including chromatin accessibility data^15,16^ and nascent transcript data^9^. Similar architectures have successfully predicted STARR-seq/MPRA data^17^ and MNase-seq nucleosome maps^18^. During training, these models learn the combinatorial interplay by which short sequence motifs, typically recognized by TFs, generate the experimental readouts for each genomic region. For example, a model trained to predict the binding of a single TF will not only learn that TF’s binding motif, but also other sequence patterns, such as motifs for cooperative binding partners^3,13^.

Discovering novel cis-regulatory features depends, however, on effectively interpreting a trained sequence-to-function model. Neural networks have traditionally been seen as uninterpretable black boxes, but thanks to several post-hoc interpretation tools specifically designed for sequence-to-function models, sequence rules can be extracted from trained models^3,19–25^. This can be done by various attribution methods, including in silico saturation mutagenesis^2,26^, integrated gradients^27,28^, or corrected gradients^23^. The attribution methods DeepLIFT^29^ and DeepSHAP^30^ use Shapley values to assign each base in the input sequence a contribution score based on how much it contributed to the predicted output. Motifs are typically among the highest contributing bases due to their crucial role in the cis-regulatory code, and they are readily summarized by TF-MoDISco^31^. Rules by which motifs cooperate with each other can also be extracted from models by systematically predicting the effect of synthetic sequences in silico^3^. However, the motifs and their interaction rules tend to be abstract, making it challenging to deduce how motifs exert their function at individual regions. Thus, while these tools have made sequence-to-function models more interpretable, they are still limited in what they reveal.

A major drawback is that current attribution strategies for sequence-to-profile models (such as the counts and profile metrics used in BPNet) rely on reductive representations of how each base impacts an output prediction. Attribution methods typically quantify an input’s effects on the entire output window, and thus do not reveal where in the experimental profile a motif exerts its influence and whether the influence is narrow or broad. Furthermore, the contribution scores of motifs represent a sum of the motif’s effects and thus do not reveal whether motifs have both positive and negative effects on the predictions. For example, a motif may cause an output feature to shift to the side, thus the motif’s effect would be positive in one part and negative in another part of the experimental profile, making it challenging to assign a single numerical score to such a motif. When such mixed contributions cancel each other out, motifs could even be missed by standard attribution methods. Finally, some bases may be assigned contribution scores because they predict experimental biases in the data. Unless such biases are explicitly regressed out^15,32^, it is difficult to identify such biases and understand their contribution to the output prediction.

Here, we overcome these obstacles by introducing a strategy called pairwise influence by sequence attribution (PISA) to visualize the range and level by which each individual base impacts each genomic coordinate at an individual locus. We implemented PISA using DeepSHAP in a package called BPReveal, which expands the capabilities of BPNet and ChromBPNet to support multiple different data types. We note however, that PISA values can be calculated in any sequence-to-profile modeling framework to visualize and interpret the learned sequence rules^2,4,8^ and that PISA values can also be calculated using saturation mutagenesis (Supplementary Fig. 1). As an example, we have implemented PISA into tangermeme, a PyTorch-based suite of tools for BPNet-like models^33^.

We first describe PISA and the two types of plots it creates for individual genomic regions: squid plots and heatmaps. We then analyze previously modeled genomics data by re-training these data on BPReveal and creating PISA plots for known regulatory regions. These plots reveal details consistent with expected biological mechanisms, as well as sequence rules that were previously hidden. They show a motif’s influence range, uncover previously hidden motifs that contain a mixture of positive and negative contributions, and reveal the experimental bias of MNase-seq that can be made into a synthetic bias track.

We then leverage the synthetic MNase-seq bias track to train a model that predicts bias-minimized MNase-seq nucleosome data^34^. These models allow us to de novo discover TF motifs important for nucleosome positioning and visualize their range of effects. We show that the effects of these motifs are bound by barriers akin to chromatin domain boundaries, which we discover de novo at higher resolution than Micro-C data. Finally, we show as proof-of-principle that the model can be used to generate sequence designs with altered nucleosome positioning, some of which we validate experimentally. These results pave the way to more systematically study the relationship between DNA sequence, nucleosome organization, and gene regulation.

## Results

### PISA reveals pairwise relationships between the input sequence and the output profile

**Fig. 1:**
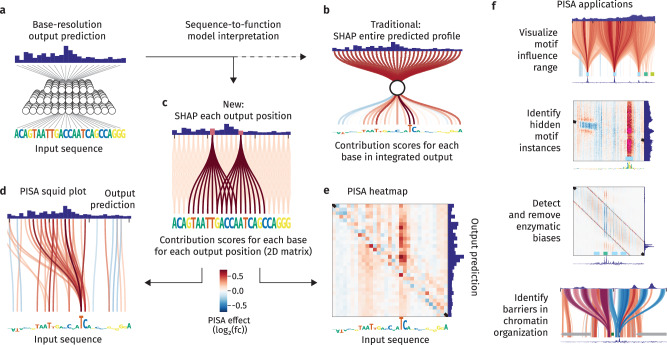
PISA and its applications. **a** A sequence-to-profile model uses DNA sequence as input and learns to predict experimental readouts from genomic assays. **b** Previous interpretation tools have assigned contribution scores to the input bases based on properties of the integrated output profile. **c** Our PISA approach assigns contribution scores for each output individually, resulting in a 2D matrix ${{\mathbb{P}}}_{i\to j}$. **d** Squid plots show high PISA values as colored lines that connect an input base to an output position. In this example, the TCA motif has a strong effect, and the bulk of this effect is to the left of the motif’s position. **e** A heatmap of the same region used in **d** shows PISA values as a colored grid. While less immediately readable than the squid plots, heatmaps are useful when multiple effects overlap or when experimental biases are present at the diagonal. **f** PISA is implemented in the BPReveal package and has multiple applications, further described in this study.

PISA is made for sequence-to-profile models that make separate predictions for each output position, e.g. each base (Fig. 1a). This feature allows attribution methods such as DeepSHAP^29,30^ to be applied to individual output positions, rather than for the entire predicted output window as done traditionally^3^ (Fig. 1b). In our implementation, we use DeepSHAP on a BPNet model and generate, for each output base *j*, contribution scores for each input base *i*. Thus, for a particular input sequence, ${{\mathbb{P}}}_{i\to j}$ represents the Shapley value assigned to base *i* from the model’s output at base *j*. ${{\mathbb{P}}}_{i,j}$ represents these values in a two-dimensional matrix (Fig. 1c).

To visualize this matrix, we made two types of PISA plots. In the **PISA squid plot** (Fig. 1d), we sought to create a simple summary of the range by which sequence patterns impact the output signal. The input bases are displayed at the bottom of the plot as traditional contribution scores, with lines drawn from base *i* to base *j* in the predicted output profile at the top. The color of the line represents the contribution score ${{\mathbb{P}}}_{i\to j}$ (positive in red, negative in blue). PISA values below a threshold are not shown for clarity.

The **PISA heatmap** (Fig. 1e) provides a finer-grained representation, where the entire PISA matrix is visualized as a colored grid: The pixel at position *i* on the x-axis and position *j* on the y-axis represents ${{\mathbb{P}}}_{i\to j}$ (positive in red, negative in blue). For reference, the traditional contribution scores are again shown at the bottom, while the output prediction is shown on the right. In this grid, the diagonal is where the input base affects the output at the same position (i.e., *i* ≈ *j*). As we will see later, this diagonal of local influence is often where experimental bias is found.

To leverage PISA and its downstream applications (Fig. 1f), we implemented a package called BPReveal. Since it encompasses the capabilities of BPNet^3^, ProCapNet^9^, and ChromBPNet^15^, BPReveal models can make use of any combination of multi-head, multi-strand architectures and leverage separate bias models to remove experimental biases from data (see architecture in Supplementary Fig. 2).

### PISA visualizes the influence range of TF motifs

**Fig. 2:**
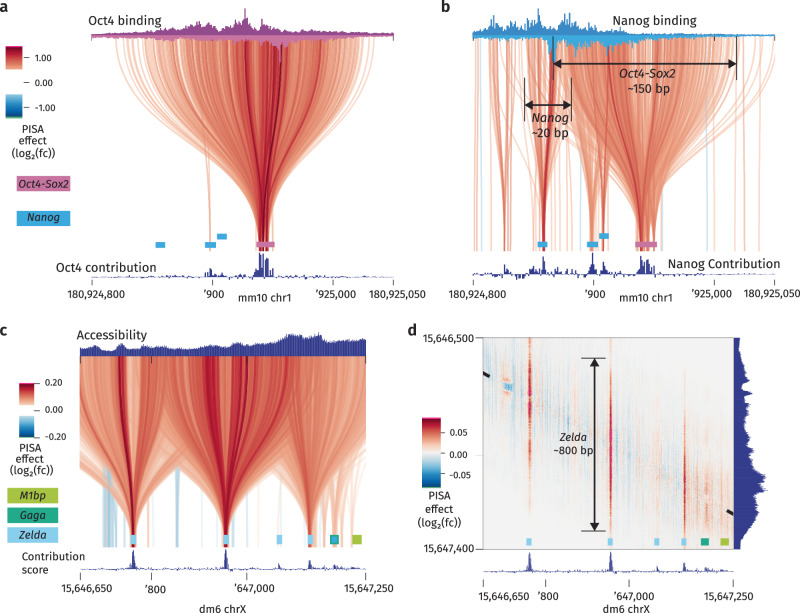
Visualizing the influence range of motifs using PISA. **a** Squid plot showing that the *Oct4-Sox2* motif (purple) determines Oct4 binding in a broad window. A multi-head model was trained on stranded high-resolution binding data (ChIP-nexus) for the pluripotency TFs Oct4, Sox2, Klf4, and Nanog. The predicted Oct4 ChIP-nexus data at the *Lefty1* enhancer are shown at the top (plus strand in dark purple, minus strand in light purple). The PISA squid plots (both strands are overlaid) show that the Oct4 task assigns broad importance to the *Oct4-Sox2* motif, consistent with Oct4 being a pioneer TF whose activity is not governed by other nearby motifs. **b** Squid plot of the same region as **a**, showing that *Nanog* motifs (blue) promote Nanog binding in a narrow range, while the *Oct4-Sox2* motif (pink) has a broad influence. To demonstrate the influence of the *Oct4-Sox2* motif, a separate model was trained only on Nanog binding. The predicted Nanog ChIP-nexus data at the *Lefty1* enhancer are shown at the top (plus strand in dark blue, minus strand in light blue). The broad effect of the *Oct4-Sox2* motif and the narrow effect of the *Nanog* motifs are consistent with Oct4 and Sox2 being pioneering TFs, while Nanog is not. **c** Squid plot of the *sog* enhancer from a model trained on bias-minimized ATAC-seq data from fly embryos^16^. The chromatin accessibility is largely determined by three motifs for the pioneer factor Zelda (cyan). While aesthetically pleasing, the squid plot is difficult to interpret due to the many overlapping lines. **d** A PISA heatmap shows the overlapping effects of the three *Zelda* motifs more clearly, and reveals that the central motif drives chromatin accessibility over a window of ~800 bp. Note that the pattern on the upper left is shown enlarged in Figure 3a, left.

To visualize how TF motifs influence output predictions, we first applied PISA to TF binding data in a well-characterized system (Fig. 2a, b). We trained a BPReveal model on previously published high-resolution ChIP-nexus data of Oct4, Sox2, Klf4, and Nanog in mouse embryonic stem cells^3^, which gave results on par with the original BPNet model (Supplementary Table 1). Since our previous model interpretation found that Nanog binding is strongly dependent on other motifs, including the *Oct4-Sox2* motif, we generated PISA squid plots for Oct4 and Nanog binding at the *Lefty1* enhancer, which contains a *Oct4-Sox2* motif and three *Nanog* motifs (Fig. 2a, b).

The PISA squid plot for Oct4 binding (Fig. 2a) showed that the *Oct4-Sox2* motif directs the Oct4 binding footprint in a broad window of ~150 bp. The *Nanog* motifs show negligible contribution to Oct4 binding. When analyzing Nanog binding in the same region (Fig. 2b), we observe that the three *Nanog* motifs contribute to Nanog binding more locally and that the *Oct4-Sox2* motif promotes Nanog binding in a broader window. This was general, as PISA plots of 800 instances of each motif confirm the broad effect of *Oct4-Sox2* and the narrow effect of *Nanog* (Supplementary Fig. 3).

This pattern is consistent with our mechanistic understanding of how Nanog binding depends on the *Oct4-Sox2* motif. Oct4 and Sox2 are pioneer TFs that remove nucleosomes^35^ and thus have effects in the nucleosomal range. Nanog, on the other hand, is a non-pioneer TF^3,36,37^ that requires the *Oct4-Sox2* motif for chromatin accessibility. Once the chromatin is open, the *Nanog* motif directs Nanog binding only locally, at the size of its own binding footprint. We conclude that broad effects are likely to involve chromatin changes, while local effects do not.

To apply PISA to a different data type, we next trained a BPReveal model to predict bias-minimized ATAC-seq chromatin accessibility data from the early *Drosophila* embryo^16^ (Fig. 2c, d). As done previously, we used the bias correction strategy from ChromBPNet^15,16^, where a bias model is trained first on closed regions that contain the bias but minimal accessibility. A second model is then added to learn the residual signal beyond the bias, thus the sequence rules that predict chromatin accessibility. Our model yields results on par with the previously published model (Supplementary Table 2) and rediscovered the motif of the key pioneer TF Zelda^16,38–40^.

Using the well-studied *sog* shadow enhancer as an example, the PISA squid plot showed each of the three *Zelda* motifs as a wide squid-like pattern reaching a region of several hundreds of bases (Fig. 2c), ~800 bp for the central one (Fig. 2d). This is consistent with chromatin-mediated effects from pioneering. We conclude that PISA directly visualizes the influence range of motifs at single loci, providing clues to the effect of those motifs on chromatin state.

### PISA reveals complex motif effects

We next used PISA to explore motifs that influence the output signal positively at some parts of the region, while having a negative influence in other parts. When using traditional attribution methods, such mixed effects might cancel each other out, causing these motifs to be misrepresented or difficult to discover.

**Fig. 3:**
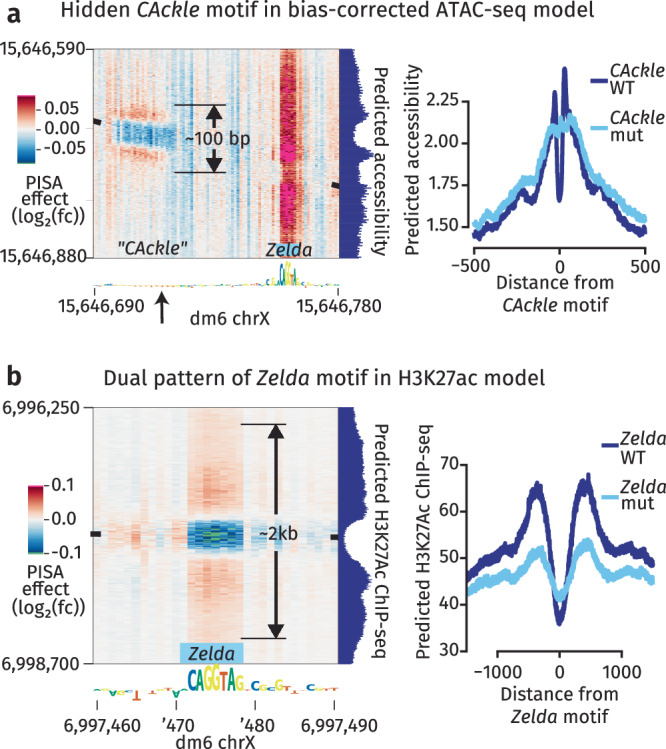
Two motifs with positive and negative effects. **a** A CA repeat creates a local dip in chromatin accessibility in ATAC-seq. (Left) The CA repeat motif, which we refer to as *CAckle*, has both negative (blue) and positive (red) contributions in a PISA heatmap, and thus is not detected in the counts contribution score (arrow). It creates a small (~50 bp) dip in accessibility surrounded by a shoulder of slightly higher accessibility, as seen in the predicted output profile on the right of the heatmap. The motif’s entire effect range is on the order of 100 bp, which is much smaller than the ~800 bp effect of the *Zelda* motif on the right of the heatmap. (Right) We simulated the effects of mutating *CAckle* motifs, and found that replacing the CA repeat with random nucleotides abolished the local dip and shoulder effect, but had a minimal effect on the global counts. **b** Positive and negative effects are a signature of histone modifications. (Left) A model trained on ChIP-seq data for H3K27Ac, a marker of enhancer activation, shows a similar negative and positive pattern around a *Zelda* motif. This is consistent with Zelda causing the nucleosomes in an enhancer to be depleted (hence the central dip) and neighboring nucleosomes to be acetylated (hence the positive shoulder). Unlike the short-range *CAckle* effect, the *Zelda* motif drives acetylation in a 2 kb window. (Right) Perturbing many instances of the *Zelda* motif shows the same effect genome-wide: Mutating the *Zelda* motif decreases both the depth of the central dip and the acetylation of flanking nucleosomes.

Serendipitously, the PISA heatmap of the chromatin accessibility at the *sog* shadow enhancer provided such a pattern (upper left in Fig. 2d, enlarged in Fig. 3a, left). This pattern showed central negative contributions (blue) that were flanked by positive contributions (red). This pattern mirrors the predicted output profile at this position, i.e. a pronounced central dip flanked by peaks on each side, as shown on the right (Fig. 3a, left). The mixed negative and positive contributions nullify each other since the count's contribution track below shows no signal (shown below with an arrow).

The sequences that produce this pattern correspond to a CA repeat (referred to as *CAckle*), which has previously been shown to boost TF binding and enhancer activity^14,41^. Although *CAckle* sites have dual positive and negative contributions, the total effect is, in some genomic instances, not neutral, allowing us to discover them across the genome using TF-MoDISco and motif scanning. To test their effect, we performed simulated mutations of 712 mapped *CAckle* motifs (Fig. 3a, right). Upon mutation, the dip with the flanking peaks flattened out, but the overall read counts remained similar, confirming a mixed negative and positive effect of *CAckle* motifs on the chromatin accessibility. We note that a *CAckle*-like motif was also learned as part of the Tn5 enzymatic bias, but these are distinct from the *CAckle* motifs that predict the dip in chromatin accessibility (Supplementary Fig. 4).

Another example of a complex motif effect is provided by ChIP-seq data of H3K27ac, a histone modification that flanks active enhancers^42^. We trained a model on H3K27ac ChIP-seq data from early *Drosophila* embryos^16^ and visualized known enhancers using PISA. This revealed that the H3K27ac profile predictions strongly depend on *Zelda* motifs (Fig. 3b, left). A *Zelda* motif creates a dip in H3K27ac at the center of the enhancer, seen as negative contributions (blue), while also promoting H3K27ac at the enhancer’s flanks, seen as positive contributions (red). This trend was again confirmed by mutating 325 *Zelda* motifs, which decreased H3K27ac at the flanks and made the central dip more shallow (Fig. 3b, right). This dual effect is consistent with Zelda’s role as a pioneer TF that depletes nucleosomes in the center, while recruiting the acetyltransferase Nejire to acetylate the flanking nucleosomes^43–45^.

The dual pattern of the *Zelda* motif in the H3K27ac model is similar to that of the *CAckle* motif in the chromatin accessibility model, but the range of the effect is an order of magnitude larger. While the contributions of *CAckle* spanned ~100 bp centered on its motif, those of *Zelda* extended to a ~2 kb region. These results show how PISA provides additional spatial resolution for interpreting how motifs exert their effect.

### PISA detects experimental biases and enables bias-corrected MNase-seq models

If PISA is able to identify how each base positionally impacts the output predictions, it should visualize experimental biases, which tend to be local. To test this, we inspected the ATAC-seq models from the early *Drosophila* embryo^16^ since we had explicitly trained a bias model that detects the local sequence preference of its key enzyme, the transposase Tn5 ^46^, before training a residual model that predicts the bias-corrected ATAC-seq data. We again generated PISA heatmaps of the *sog* shadow enhancer, but this time, we separately inspected the uncorrected model (which is the bias and residual model combined), the bias model, and the residual model.

**Fig. 4:**
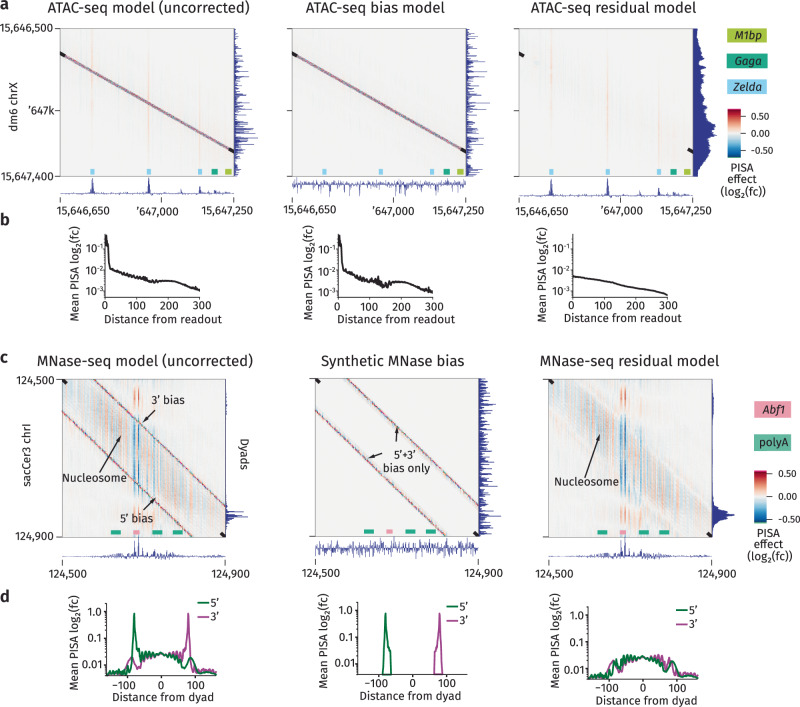
PISA reveals enzymatic biases. **a** The ChromBPNet bias correction strategy effectively removes experimental bias. (Left) A model trained on ATAC-seq endpoints in accessible regions learns the effects of motifs (faint vertical bands), but also learns enzymatic bias (strong diagonal band). (Center) A model trained on closed regions of chromatin learns enzymatic bias, but not pioneering motifs. (Right) The ChromBPNet architecture removes the patterns learned by the bias-only model and assigns importance only to the pioneering motifs. The tracks below all PISA heatmaps show the read count contributions, which capture the total accessible signal well, while the profile contributions more strongly capture the experimental bias (Supplementary Fig. 5). **b** A base attribution total (BAT) plot quantifies the strength of the bias. The first two models show a large spike in contribution near the diagonal of the PISA heatmap, while the right plot shows no such central spike. **c** PISA enables bias-minimized MNase-seq models. (left) A model trained on MNase endpoints learns the enzymatic bias twice: Once on the $5{^\prime}$ end of the nucleosome-sized fragment and once again on the $3{^\prime}$ end. By aligning and subtracting the PISA values for each strand (see section synthetic bias calculation), we construct a synthetic bias track that can be used to correct MNase sequence bias using the ChromBPNet architecture. Tracks below all PISA heatmaps show the tracks for profile contributions, not count contributions, since the total read counts do not substantially change across regions when predicting nucleosome profiles. **d** A BAT plot quantifies the effectiveness of MNase bias removal. The large spikes of importance at +80 and –80 are enzymatic bias, and these spikes are eliminated in the bias-corrected model (right).

In the uncorrected model, the ATAC-seq bias is visible as a diagonal band and overshadows the weaker effects of the three *Zelda* motifs that appear as vertical stripes (Fig. 4a, left). In the bias model, only the diagonal band is left (Fig. 4a, center), while the residual model only shows the effects of the three *Zelda* motifs (Fig. 4a, right). This confirms that the diagonal band is indeed the bias and that the bias was successfully removed in the residual model.

As a method for quantifying the effectiveness of the bias removal, we created a base attribution total (BAT) plot, which shows the average contribution of each input base to the output at a given distance (Δ) from the diagonal (Fig. 4b). More precisely, ${{{\rm{BAT}}}}(\Delta )={{{{\rm{mean}}}}}_{i}({{\mathbb{P}}}_{i\to i+\Delta })$, where Δ is the x-axis in the heatmap. As expected, BAT plots for both the uncorrected and the bias model show very strong signal within 10 bp (Fig. 4b left, center), while this local signal is not present in the bias-corrected model (Fig. 4b, right).

Having confirmed that PISA reveals experimental bias, we next turned to MNase-seq data. MNase has a strong AT sequence bias^34,47^, but it is difficult to separate this bias from sequence features that determine the nucleosome organization. We reasoned that a bias-minimized MNase-seq model would predict a cleaner nucleosome profile and enhance interpretation^15^.

We trained a BPReveal model on MNase-seq data from *S. cerevisiae* since yeast nucleosome occupancy is well-studied^48–50^, the small genome size permits very high coverage data^51^, and we can benchmark our results against previous deep learning models^26^. Since the MNase enzymatic bias is found at the fragments’ end, we recorded each fragment’s $5^{\prime}$ and $3^{\prime}$ ends as two separate tracks and trained BPReveal to predict both tracks simultaneously.

BPReveal achieved high prediction accuracy on experimental data from held-out chromosomes (Supplementary Table 3). It achieved higher prediction accuracies than the model by Routhier et al.^26^, using the same training data, even when the performance was scored by Pearson correlation, which is part of the loss function in the Routhier model (Supplementary Table 4). This suggests that BPNet-derived models trained with the BPReveal framework are well-suited to learning yeast MNase-seq data.

We then assessed whether PISA could detect the experimental bias of MNase-seq. PISA heatmaps showed a strong, narrow diagonal band, either to the left (in the $5^{\prime}$ model) or the right (in the $3^{\prime}$ model) of the nucleosome-sized fragments (Fig. 4c, left). This signal represents highly local effects in BAT plots (Fig. 4d, left), suggesting that local enzymatic sequence bias strongly contributes to the predictions. Apart from the bias, the PISA heatmaps also showed subtle positive and negative contribution scores within the nucleosome-sized fragments (shown combined in Fig. 4c, left). This signal likely represents intrinsic DNA sequence properties that determine the readiness to wrap around the histone core and form stable nucleosomes^50,52–55^.

We next explored ways to extract the bias from the PISA values and derive a synthetic bias track. The model’s independent prediction of the $5^{\prime}$ and $3^{\prime}$ ends serendipitously provided us with a method to extract the bias. The reasoning is as follows: If a particular base affects a nucleosome, then it should affect the entire nucleosome, including its $5^{\prime}$ end and $3^{\prime}$ end. But if a base only affects one endpoint of the nucleosome and not the other, then it is likely a bias signal. Since the two tracks differ in the bias but essentially not in the biological signal, subtracting their PISA heat maps cancels out the biological portions and leaves behind pure enzymatic bias. The effectiveness of this approach is seen in BAT plots (Fig. 4d).

The second innovation came from the insight that the efficiency property of Shapley values allows us to sum up the PISA values from the bias and construct a genome-wide synthetic MNase bias track to train a bias model. We confirmed (using TF-MoDISco) that the model did not learn any TF motif (Supplementary Fig. 6). We note that a synthetic bias track from as few as 1,500 regions produced equivalent results (Supplementary Fig. 7).

We then used this bias model to train and cross-validate a ChromBPNet-style model that captures the bias-minimized nucleosome signal in the residual model. PISA heatmaps confirmed that we have successfully separated the MNase-seq bias and nucleosome signal (Fig. 4c). A BAT plot of the corrected model also shows that the bias signal is largely absent and no longer stands out above the biological signal.

To benchmark the bias removal, we compared our results to those of other MNase bias removal methods (Supplementary Fig. 8). We trained a bias model on MNase-seq data obtained from naked DNA^51,56^ or used the statistical modeling tool seqOutBias^32^ before training on the MNase-seq data. BAT plots show that these methods reduced the bias signal, and quite considerably in the case of the seqOutBias-corrected data, but both methods left more uncorrected enzymatic bias than our corrected prediction (Supplementary Fig. 8).

Thus, PISA not only visualized the bias in MNase-seq data, but also allowed us to derive a synthetic bias track to train a bias-minimized MNase-seq model.

### PISA visualizes how de novo discovered motifs affect nucleosomes

**Fig. 5:**
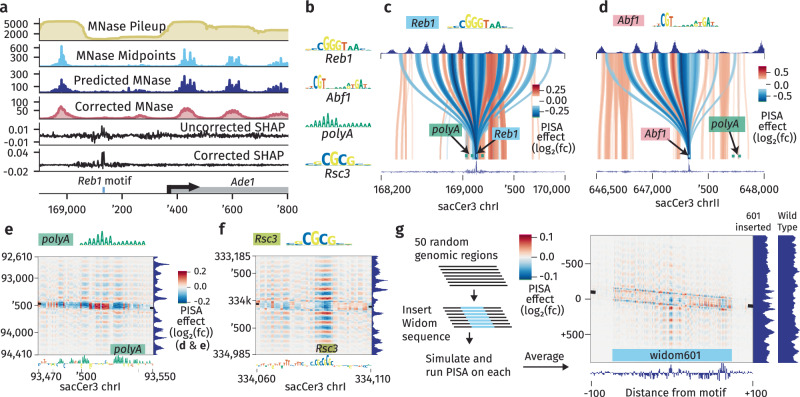
The bias-corrected MNase-seq model discovers motifs de novo that positions nucleosomes. **a** The model learns and corrects MNase data. The top track shows the MNase-seq data as a fragment pileup, a common way of plotting such data. Our models instead predict the endpoints of the fragments, providing higher resolution data. For convenience, we present the model’s predictions as midpoints. The model’s predictions match the experimental data well, and the corrected data remove much of the high-frequency noise in the uncorrected data. The importance of this correction is most visible in the contribution scores, which are noisy and fuzzy in the uncorrected model but clearly show a *Reb1* motif in the corrected model. **b** Four motifs identified by our bias-corrected MNase-seq model. **c** A PISA squid plot of the region in **a**. The central *Reb1* motif shows a long-range ringing effect. **d** A representative *Abf1* motif shows a similar long-range ringing effect. This particular motif instance shows a striking leaning effect, where the motif drives nucleosome positioning mostly to its left. **e** The *polyA* motif is more subtle than *Reb1* or *Abf1*. It tends to be more distributed, as in this example where a block of *polyA* has been annotated by TF-MoDISco, but several smaller clusters of A also contribute to position nearby nucleosomes. **f** The *Rsc3* motif also creates a ringing effect. In this instance, it is coupled with several short T repeats, which likely serve as a very degenerate *polyA*. **g** The widely-used Widom 601 sequence that strongly positions nucleosomes in vitro does not have a strong effect in vivo, consistent with previous observation^68^. PISA was applied to 50 random genomic regions where the Widom 601 sequence was inserted and then averaged. The average output prediction (601 inserted) is also shown to the right, in comparison to those of the wild-type sequences. The effect is, on the whole, very small. The complete analysis showing all plot types for all motifs is in Supplementary Fig. 9.

We next examined what the MNase-seq model learned. For an intuitive reading of the MNase-seq data, we displayed the midpoints of the fragments representing the nucleosome dyads, rather than the positions of the $5^{\prime}$ and $3^{\prime}$ ends that we trained the model on. As seen at the *Ade1* gene example locus, a known genetic marker in yeast, the experimental and predicted midpoint data look spiky. After removal of the bias, the nucleosome signal was smoother, and the background signal in the profile contribution scores was markedly reduced, revealing motifs such as *Reb1* (Fig. 5a).

We then ran TF-MoDISco to discover motifs de novo using either the contribution scores from the uncorrected model, the bias-only model, or the bias-corrected model. The bias-only model learned many motifs related to the MNase sequence bias, most notably sequences that transition from AT-rich to GC-rich regions, consistent with previous studies^34,47^ (Supplementary Fig. 6). The uncorrected model also learned biologically relevant motifs, but it was difficult to distinguish them from the many bias motifs it had also learned. In contrast, the motifs discovered from the bias-corrected model were recognizably biologically relevant and known to affect nucleosomes, including *Abf1* and *Reb1*, *polyA* motifs, CG-rich sequences, and a CGCG motif^49,57–59^ (Fig. 5b).

While these motifs were known, our model discovered and mapped these motifs genome-wide and helped provide insights into their effect sizes and ranges ^18,26,60^. The CGCG motif has been characterized as a recognition signal for Rsc3, a component of the RSC remodeler complex^61,62^, but the frequency of such motifs in the genome is unknown. *polyA* sequences resist DNA bending and are often found between nucleosomes^63–65^, but the exact sequence nature and context by which such sequences affect nucleosomes is not known. We therefore scanned the genome for motifs contributing to the nucleosome predictions and visualized their effects with PISA (Supplementary Fig. 10). As a control, we compared our discovered motifs to those discovered by traditional motif scanning.

PISA squid plots showed a striking effect of *Reb1* and *Abf1* motifs (Fig. 5c, d). Both induce a strong oscillating positional signal of ~750 bp (or 4-5 nucleosomes) emanating from the motif, usually extending in both directions but sometimes leaning towards one side. We discovered 656 *Abf1* motifs and 760 *Reb1* motifs, the majority of which were discoverable with traditional motif scanning (Supplementary Fig. 10). By contrast, the 1,543 *Rsc3* (CGCG) and 12,791 *polyA* motifs mapped by the model could not be identified by traditional motif scanning without discovering a large number of false positive motifs not associated with nucleosome positioning (Supplementary Fig. 10). This suggests that the model learned finer-grained combinatorial sequence context beyond individual motifs. Consistent with this, PISA squid plots and PISA heatmaps showed that the positioning effects of *Rsc3* motifs (Fig. 5(f)), and especially those of *polyA* motifs (Fig. 5e), were weaker, less localized and more enigmatic than those of *Reb1* and *Abf1* motifs (Supplementary Fig. 9). Since the weak *Rsc3* and *polyA* motifs were very abundant across the genome, their combined effect nevertheless made a substantial contribution to the nucleosome signal (Supplementary Fig. 10).

To put these effects into context, we asked what pattern the model would predict for the Widom 601 sequence, a ~150bp-long sequence often used by in vitro experiments to position nucleosomes^66^. To provide a neutral sequence context, we injected the 601 sequence into 50 random sequences, performed PISA on each prediction, and averaged the results (Fig. 5g)^67^ and points to differences between in vitro and in vivo experiments.

In summary, our model interpretation revealed that motifs play a major role in positioning nucleosomes across the genome, which is surprising given that previous debates in the field have mostly focused on the intrinsic effects of DNA sequence^50,57,58,68–73^.

### Barrier elements limit the effects of nucleosome-positioning motifs

PISA plots showed that a motif’s long-range nucleosome positioning effect, particularly around promoters, often leans to the left or right (Fig. 5d, Supplementary Video 1). We hypothesized that this is because motifs do not function in isolation, but that their effect on nucleosomes is influenced by other nearby motifs or motif combinations. Such sequences may correspond to domain boundaries seen in Micro-C data^74^ since there is a close link between nucleosome positioning and 3D chromatin organization in yeast^75^.

**Fig. 6:**
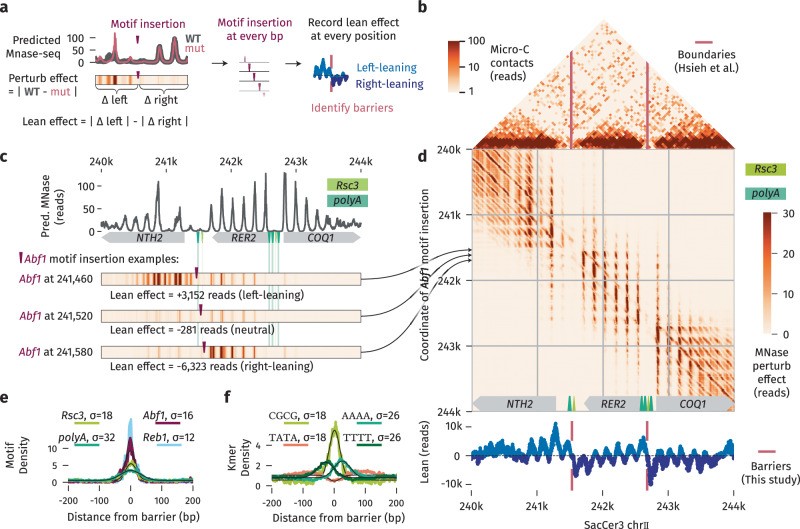
A model for nucleosome positioning learns chromatin organization. **a** Process for identifying barrier elements. (left) After inserting a strong positioning motif (purple pip), the changes in predicted nucleosome positions (pink line) from the wild-type prediction (gray) are recorded. The difference is summarized below as a heatstrip, showing that most effects are left of the insertion, resulting in a positive lean value. (center) The motif is injected at every possible position in the genome to record the lean effects. (right) Positions where lean values flip from positive (cyan) to negative (navy) represent a barrier (pink). **b** Published Micro-C data^75^ with identified boundaries (pink) for a region around the *RER2* gene. **c** Example insertions from the *RER2* region. (top) The wild-type MNase-seq prediction shows a wide nucleosome-depleted region between *NTH2* and *RER2*, a promoter region that contains mapped motifs. (bottom) Insertion of an *Abf1* motif at three positions (purple ticks) leads to dramatically different effects as seen on the heatstrips. The effect is left-leaning at position 241,460, overall weak at 241,520, and right-leaning at 241,580. This suggests that sequence elements near 241,520 limit the effect of motif insertions. **d** (top) When the motif is systematically injected at every position in the *RER2* region, the resulting heat map resembles the Micro-C contacts shown in **b**. (bottom) Lean effects for every motif insertion show positive values (cyan) for left-leaning and negative values (navy) for right-leaning effects. At a barrier (pink), the lean values flip from positive to negative. **e** Motif enrichments at all identified barriers in the yeast genome show that the *Abf1* and *Reb1* motifs are strongly enriched within 20 bp of the barrier positions. The *polyA* motif is similarly enriched, but over a broader window of 30 bp, likely because *polyA* has an asymmetric effect, and motif direction was not considered in this plot. **f** Short sequence elements enriched around barriers include CGCG, as well as AAAA, here seen with its characteristic asymmetric distribution mirrored by the opposite pattern for TTTT. TATA, which can be a core promoter element, shows a slight underenrichment near the identified barriers.

If domain boundaries block the effect of nucleosome-positioning motifs, we would expect to identify such barriers if we systematically insert nucleosome-positioning motifs at various positions along a genomic sequence (Fig. 6a). When the effect of the motif is attenuated on one side, we can quantify the “lean effect” as the difference between the effect on the left versus the right (Fig. 6a).

We tested this approach on a region around the *RER2* gene (Fig. 6c). This region was not part of the training set and contains two Micro-C domain boundaries that overlap with motifs mapped with our MNase-seq model. We systematically injected an *Abf1* motif at every base-pair position in this region and predicted the perturbation effect (wild-type – mutant). We indeed observed that inserting the motif slightly to the left of the first barrier produced nucleosome perturbations to the left of the motif, while inserting slightly to the right produced right-leaning effects (Fig. 6c). Moreover, when these perturbation effects were visualized for every motif insertion position (Fig. 6d), they resemble Micro-C interaction maps^74^ (Fig. 6b). This was also true for Micro-C data obtained in vitro^76^, when we performed similar perturbations on the *GPA2*-containing region (Supplementary Fig. 11a).

We therefore systematically performed motif perturbations across the genome using four different motifs and identified 4,498 barriers where the lean score consistently flips from left to right. The majority of these barriers are positioned near Micro-C-derived boundaries^74^, with more than half of them within <100 bp distance. Like Micro-C boundaries, our barriers are also preferentially found at promoters and are enriched for *Abf1*, *Reb1*, *Rsc3* and *polyA* motifs (Fig. 6e). Notably, our barriers contain these motifs at closer distances (e.g. *Abf1* is *σ* = 16bp versus *σ* = 34bp for the Micro-C boundaries). This suggests that our method of identifying barriers revealed domain boundaries at higher resolution than is currently possible with Micro-C data (Supplementary Fig. 11d).

We conclude that barriers or domain boundaries are indeed composed of nucleosome-positioning motifs. Unexpectedly though, we found that the majority were either not near a mapped TF motif or not near an experimentally determined ChIP-exo binding peak of Abf1, Reb1 or Rsc3 (Supplementary Fig. 11f). Instead, barriers or micro-C boundaries are often composed of multiple *polyA* motifs (Supplementary Fig. 11f), which cannot be mapped experimentally since no known yeast TF binds them.

To confirm the presence of short motifs, we analyzed the kmer distribution around barriers or Micro-C boundaries (Fig. 6f, Supplementary Fig. 11e). In addition to the CGCG kmer, AAAA kmers were indeed enriched and showed a strand-specific pattern on each side. These results suggest that combinations of *polyA* motifs can form domain boundaries through a mechanism that is not understood. Since *polyA* motifs have poor information content and are difficult to score in genomic sequences by traditional motif scanning methods, these results highlight the power of deep learning models to capture their effect on nucleosomes.

We conclude that our model trained on MNase-seq data has learned intricate rules by which motifs position nucleosomes and that motif combinations form barriers that resist the effect of motif perturbations.

### BPReveal allows the engineering of sequences with altered nucleosome properties

With the advent of deep learning in genomics, there has been a push to use model predictions to streamline the design of synthetic sequences^77,78^. Having trained a bias-minimized nucleosome model with the help of PISA, we tested whether the model was accurate enough to design sequences with altered nucleosome configurations. For this, we implemented a genetic algorithm (GA) in the BPReveal package, which finds sequence mutations that maximize a desired experimental outcome, defined and bounded by the user.

**Fig. 7:**
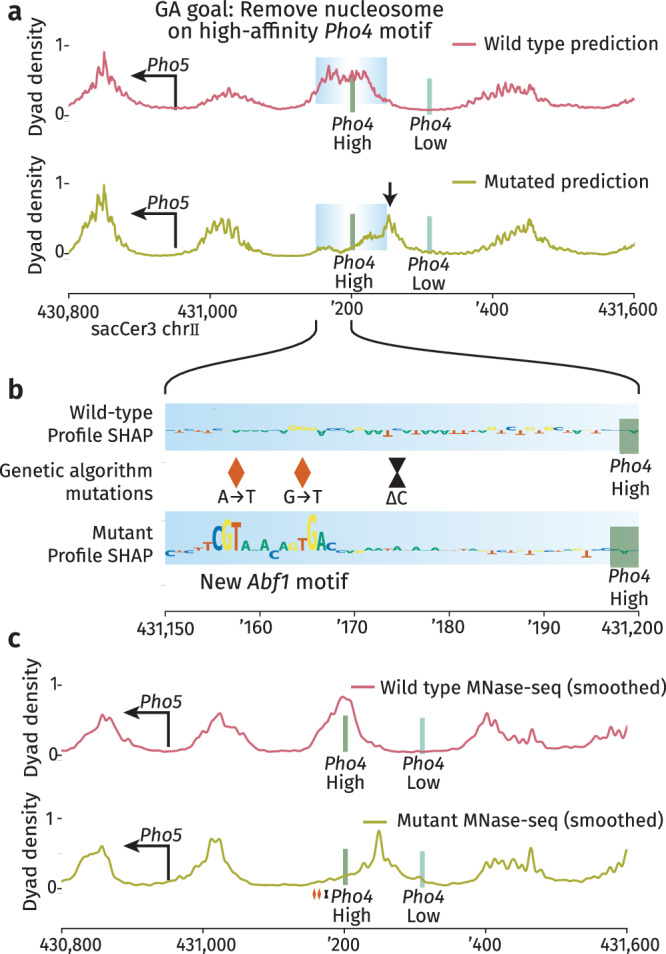
BPReveal’s genetic algorithm (GA) generates sequence designs for nucleosomes that can be experimentally validated. **a** The GA feature in BPReveal was directed to reduce nucleosome occupancy in the blue region centered on the high-affinity *Pho4* motif with three mutations. It returned mutations that induce a predicted shift of the nucleosome to the right side (indicated with an arrow). **b** The mutations introduced a new *Abf1* motif (orange diamonds) about 20 bp away from the *Pho4* motif, as visualized by the contribution scores obtained with DeepSHAP. An additional mutation (black hourglass) has only a small effect. **c** Experimental validation of this sequence design was performed by CRISPR/Cas9-mediated editing of the endogenous yeast PHO5 locus and performing MNase-seq on the wild-type and mutant strain (shown smoothed as there is no bias correction). This confirmed that the nucleosome is shifted from the *Pho4* motif, while leaving the rest of the nucleosome landscape largely unperturbed.

We used the well-characterized *Pho5* promoter, which is bound and induced by the TF Pho4 under phosphate starvation^79,80^ (Fig. 7). Under repressed conditions (i.e., high phosphate), a low-affinity *Pho4* motif is exposed, while a high-affinity *Pho4* motif is covered by a nucleosome^81–83^ (Fig. 7a). Since previous studies have analyzed the consequences of mutating the two *Pho4* motifs^84^, we decided to manipulate the nucleosome configuration without perturbing the motifs.

We used the GA to create a sequence design that minimizes the nucleosome on the high-affinity *Pho4* binding site with a maximum of three mutations. We obtained a design where two of the mutations introduce a new *Abf1* motif, which is predicted to create a nucleosome-depleted region and expose the *Pho4* motif by shifting the nucleosome to the right (arrow in Fig. 7a). A third mutation only slightly enhanced the effect (Fig. 7b). By repeating the GA sequence search, we found that the introduction of TF motifs was a frequent solution. Of the 409 different runs, 77 of them introduced a new *Abf1* motif, and 122 introduced a *Reb1* motif.

To validate this prediction, we created a yeast strain where we introduced the three mutations via CRISPR/Cas9-mediated editing. We then performed MNase-seq to map the nucleosomes in the wild-type strain and the mutant strain (Fig. 7c). We found that the nucleosome that covers the *Pho4* binding site in wild-type is shifted to the right in the mutant, thereby exposing the high-affinity *Pho4* site, as we had intended (Fig. 7c). We also performed MNase-seq on two other designed mutants. One of these agreed with our predictions, showing a shifted nucleosome to the left of the high-affinity site, whereas in the other, the model mis-predicted a stable nucleosome at the same position (Supplementary Fig. 12).

These experimental results demonstrate that our nucleosome model allows the design of strains with altered nucleosome properties, which represents an opportunity for simultaneously studying the role of DNA sequence, nucleosome positioning, and gene regulation in the future. Taken together, this highlights the use of PISA in creating synthetic bias tracks for MNase-seq data, enabling bias-minimized nucleosome models for unprecedented interpretation and sequence designs.

## Discussion

PISA is, at its core, a way to visualize how one stretch of DNA affects a biological signal in its surrounding region. We are not the first to ask this question, as classical genetic screens and statistics-based approaches of analyzing genomics data have long provided useful insights^85^. With the recent introduction of interpretable deep learning techniques in genomics, we now have an opportunity to investigate ever-finer positional details of the sequence-to-function relationships that ultimately form the cis-regulatory code. PISA adds to the current set of interpretation tools and has multiple distinct advantages.

PISA works on individual loci in a wild-type context. Traditional statistics-based methods are good at identifying abstract patterns and associations in genomic data, but applying these rules at individual regions of interest has been very challenging. Since deep learning models are trained to make accurate predictions for each region, they inherently learn how to identify and combine multiple sequence elements to predict the experimental outcome in a given region. However, current attribution approaches do not fully capture subtle cis-regulatory rules. PISA bridges this gap by providing a precise map by which relevant sequence elements drive genomics readouts at single-base resolution.

PISA plots are inherently visual and intuitive. We have used PISA heatmaps and squid plots for four different experimental datasets from three different organisms, and find the patterns to be consistent across the genome and explainable by known mechanisms. For example, in a model of TF binding in mESCs, PISA revealed a different influence range of the *Oct4-Sox2* and *Nanog* motif, consistent with their distinct effects on chromatin accessibility^36^. We also discovered a strong, localized footprinting effect on chromatin accessibility by a CA-repeat motif, *CAckle*, which is obscured when using traditional attribution methods but known to be functional in transcription studies^14,41^. Finally, we observed a leaning motif effect of nucleosome-positioning effects in PISA plots, which inspired our investigation into chromatin domain boundaries.

PISA provides an avenue for discovering and extracting specific patterns, such as experimental biases. Enzymatic biases in genomics assays are typically mixed in with the biological signal, and thus hard to distinguish with traditional attribution methods. In PISA heatmaps, the contribution scores of local enzymatic biases are distinguishable as distinct diagonal bands. In the case of MNase-seq, we leverage this property to create a synthetic bias track, which in turn can be used to train a bias-minimized MNase-seq model.

The ability to train a bias-minimized MNase-seq model from sequence alone and discover TF motifs that contribute to the nucleosome organization is unprecedented. Using yeast data, we discovered and visualized the long-range nucleosome positioning effects of motifs, including *polyA* motifs that were previously difficult to capture computationally or experimentally. We further showed that these motifs function as barriers that correspond to domain boundaries in the 3D chromatin organization, and designed minimal mutations that precisely reposition nucleosomes in vivo.

Our approach to identify barriers is complementary to those using Micro-C data, but has higher resolution and considerably lower sequencing depth requirements for larger genomes. We expect this approach to be applicable to more complex organisms, as long as the MNase-seq data are of high quality and high coverage.

Taken together, BPReveal and PISA provide a useful method for training a variety of experimental data and visualizing the intricate rules of the cis-regulatory code across various species. The approach is particularly useful for mapping and understanding the sequence basis of nucleosome positioning, enabling us to experimentally probe the role of nucleosomes in the cis-regulatory code.

There are limitations of PISA that should be kept in mind. First, PISA plots are only as good as the underlying model, and thus careful performance benchmarking is required before investing into interpretation. Second, PISA reveals sequence rules that the model learned, but not the proteins and regulatory mechanisms that create these rules inside cells. For example, PISA revealed that the *Zelda* motif not only drives chromatin accessibility but also the acetylation on the flanking nucleosomes. Supported by previous data^44–46^, we can guess that Zelda interacts with an acetyltransferase, but we cannot rule out that the effect is mediated indirectly through the creation of open chromatin. Third, we do not currently have a mathematical approach to extract general rules from PISA data. For example, PISA plots make it clear that our models have learned aspects of cooperativity, but we lack a formalism that would allow us to directly extract the relationships between motifs. Such gaps in knowledge are an opportunity for additional innovations, with the long-term goal of linking sequence rules and mechanisms in a coherent framework.

## Methods

### Data

#### ChIP-nexus

A BPReveal model was trained on Oct4, Sox2, Klf4, and Nanog ChIP-nexus experiments in mouse R1 mESC lines using the same processed bigwig tracks and peak coordinates as in the original study (GSE137193^86,87^). To the extent possible, we maintained the same model parameters as used previously.

#### ATAC-seq

A ChromBPNet-style model^15^ was trained on ATAC-seq data from 2-3h *Drosophila melanogaster* embryos using the BPReveal framework. The same processed bigwig files, peak coordinates, and model parameters were used for training (GSE218852^16^).

#### Published MNase-seq data

We used a published MNase-seq data set, specifically the wild-type experiments SRR12073988 and SRR12073989 from GSE153035^51^. Data were downloaded using sratoolkit version 3.2.0^88^. Paired-end reads were aligned against the sacCer3 genome using bowtie2 –very-sensitive -X 1000^89^ (Bowtie2 version 2.5.1). Aligned fragments that spanned more than 1 kb were eliminated, but no other size selection was performed. For all paired reads, we created coverage tracks of the $3^{\prime}$ endpoints, $5^{\prime}$ endpoints, and fragment midpoints. ($3^{\prime}$ and $5^{\prime}$ in this context are with respect to the genome; therefore all fragments are effectively treated as being on the positive strand.) The code used for this processing is included in the code repository for this paper at https://github.com/zeitlingerlab/bpreveal-manuscript.

For the performance comparison against Routhier et al.^26^, we used the tracks in the GitHub repository for that paper.

#### Micro-C data

We used the Micro-C data from Hsieh et al.^74^, with GEO accession GSE68016. Specifically, we used the wild-type replicates with accession numbers GSM1661107 to GSM1661126. Data were aligned to the sacCer3 genome using bowtie2 -U –very-sensitive-local, and reads were selected for plotting using custom scripts, which are available in the code repository for this manuscript. Briefly, all paired reads entirely contained in the display window were used to build the contact map, and each pixel in the contact map represents 50 bases.

To compare our identified barriers to those identified with Micro-C, we used the set of boundaries published previously^74^.

#### Histone modification ChIP

We used published H3K27ac ChIP-seq data from refs. ^16^, specifically from the GSM6757761, GSM6757762, GSM6757763, and GSM6757764 datasets. We used the same pipeline to call peaks and process bigwig files.

#### Custom yeast mutants

The *S. cerevisiae* strain BY4741, a derivative of S288C with the genotype MATa his3Δ1 leu2Δ0 met15Δ0 ura3Δ0, was used. A single isolate was whole-genome sequenced before strain construction. The three-point mutations were introduced using CRISPR/Cas9-based genome editing as described previously^90^. A 20 bp gRNA close to the mutation site was designed with BsaI overhangs and ordered as oligonucleotides. The gRNA-encoding oligonucleotides were annealed and cloned into the pCASB plasmid (Addgene 190175) using BsaIHF-v2 enzyme and T4 DNA ligase. The cloning reaction was transformed into *E. coli* and plated on Kanamycin selection plates. The plasmids were then isolated and verified by Sanger sequencing. A 160 bp homology-directed repair (HDR) template was designed to contain the desired mutant sequence, synthesized by Genescript and amplified by PCR. Yeast cells were then co-transformed with the cloned plasmid encoding Cas9 and gRNA, along with the HDR template. Transformants containing pCASB were selected on G418-containing media. Colonies were streaked twice, and isolates were screened for loss of the gRNA plasmid via replica plating to G418. For each isolate, genomic DNA was extracted, and the region of interest was amplified by PCR, purified, and verified by Sanger sequencing. DNA sequences for the gRNA and HDR templates are included in Supplementary Data 1.

#### MNase-seq experiments

MNase-seq experiments were performed essentially as described previously^91^ in replicates. Yeast cultures were grown at 30 ^∘^C in YPD to OD_600_ 0.81 and crosslinked with 1% formaldehyde for 15 minutes at room temperature. 125 mM Glycine was added to quench the reaction. Cell pellets were resuspended in spheroplasting buffer (1M Sorbitol, 5 mM *β*-mercaptoethanol, 50mM Tris pH 7.5, 2 mg/ml zymolyase (AMS Bio cat no: 120493-1); 1 mL of buffer per 20 mL of cell culture) and incubated for 15 min. at room temperature. The derived spheroplasts were treated with 100U MNase (NEB cat no: M0247S) for 30–40 min at 37 ^∘^C. The reactions were stopped by the addition of EDTA pH 8.0 (50 mM final conc.), and EGTA pH 8.0 (50 mM final conc.). Samples were then incubated with RNase A (Thermo Scientific, EN0531, final conc. 0.2 mg/ml) at 42 ^∘^C for 30 min to digest RNA. The crosslinks were reversed by adding SDS and proteinase K (Invitrogen cat no: 25530049, 1mg/ml final concentration) and incubation at 65 ^∘^C for 45 min. DNA was extracted using the Monarch PCR & DNA cleanup kit. Samples were resolved on 1% agarose gel to evaluate the digestion. Mononucleosome-sized bands were extracted, and libraries were constructed from 10ng purified DNA using the Watchmaker DNA Library Prep kit (cat no. 7K0102-096) from Watchmaker Genomics according to the manufacturer’s instructions. Paired-end sequencing was performed on AVITI (2x 75bp cycles). The data processing was identical to that used for the published MNase-seq samples. Reproducibility metrics showed good agreement between the profiles for all of the different strains (Supplementary Table 5).

### BPReveal implementation

The BPReveal package is a suite of tools for flexible training and interpretation of BPNet-derived sequence-to-profile models^3,9,15^. The code base is licensed under the GNU General Public License (Version 2 or later) and is available at https://github.com/mmtrebuchet/bpreveal. The architecture is shown in Supplementary Fig. 2. The input is a one-hot encoded DNA sequence, with typical input lengths around 3,000 bp. The models consist of an initial convolutional layer, followed by a stack of dilated convolutions that exponentially increase in receptive field. There are two outputs for each dataset: (1) the “profile” output is a vector representing the log-probability (i.e., logits) of finding a read at each position in the output window; (2) the “counts” output is a scalar representing the natural logarithm of the total number of reads observed in that window. For brevity, the two outputs are referred to as one “head” per dataset. The model can be trained as a multi-task model on several experiment types simultaneously, including but not limited to ChIP-seq, ATAC-seq, MNase-seq, and PRO-cap data.

BPReveal includes two significant expansions from the original BPNet architecture that were first implemented in ChromBPNet^15^ and ProCapNet^9^. First, instead of giving convolutional filters beyond the input window zeros, as is often done in image processing, the input length is increased to provide a DNA sequence for the entire receptive field ^15^. Second, multiple strands from one experiment can be combined into one output. The log-counts output then combines the total reads from both strands, and the profile logits have the shape (output-length, num-strands). This benefits the training of data such as PRO-cap, where reads are often primarily on one strand^9^.

The loss is the same as it was in the original BPNet:

1 $${{{{\rm{loss}}}}}_{{{{\rm{head}}}}}=	{\lambda }_{{{{\rm{head}}}}}{\left(\ln ({n}_{{{{\rm{head}}}}}^{{{{\rm{obs}}}}})-\ln ({n}_{{{{\rm{head}}}}}^{{{{\rm{pred}}}}})\right)}^{2}\\ 	 -{\gamma }_{{{{\rm{head}}}}}\ln \left({{{{\rm{P}}}}}_{{{{\rm{mult}}}}}({\vec{{{{\bf{k}}}}}}_{{{{\rm{head}}}}}^{{{{\rm{obs}}}}}| \vec{{{{\bf{p}}}}}_{{{{\rm{head}}}}}^{{{{\rm{pred}}}}})\right)$$

and

2 $${{{\rm{loss}}}}={\sum}_{{{{\rm{head}}}}\in {{{\rm{heads}}}}}{{{{\rm{loss}}}}}_{{{{\rm{head}}}}},$$

where *γ*_head_ and *λ*_head_ are (user-assigned) weights to the profile and counts components of the loss for each head, $\ln {{{{\rm{P}}}}}_{{{{\rm{mult}}}}}(\vec{{{{\bf{k}}}}}| \vec{{{{\bf{p}}}}})$ is the log-likelihood of observing the outcome $\vec{{{{\bf{k}}}}}$ (i.e., the observed experimental reads) given the probability distribution $\vec{{{{\bf{p}}}}}$ (i.e., the predicted logits from the model), and *n* is the total number of reads observed (or predicted) over an entire region.

We have designed BPReveal to be performant even on commodity hardware. The rapid turnaround time on training and interpreting enables exploration and tuning that are not practical for large models that take days to train (Supplementary Table 6). BPReveal is built using bedtools v2.31.1^92^, cpython 3.12.11, gcc 15.1.0, h5py 3.14.0, Keras 3.11.3^93^, Modisco 2.4.0^31^, numpy 2.0.1^94^, pybedtools 0.12.0^95^, pybigwig 0.3.24^96^, pysam v0.23.3^97^, scipy 1.16.2^98^, TensorFlow 2.20.0^99^, tf-keras 2.20.1, memesuite 5.5.8^85^, and samtools 1.20^100^.

### Bias regression

BPReveal includes a tool based on ChromBPNet^15^ to remove experimental biases from genomics data. The technique used in ChromBPNet was based on the assumption that

3 $${{{{\rm{P}}}}}_{{{{\rm{bias}}}},i}*{{{{\rm{P}}}}}_{{{{\rm{biology}}}},i}={{{{\rm{P}}}}}_{{{{\rm{experiment}}}},i},$$

where P_bias,*i*_ represents the probability density at base *i* due only to experimental biases, P_biology,*i*_ represents the probability density of the true biological profile at base *i*, and P_experiment,*i*_ is the probability density that would be measured by an experiment. Since BPReveal and ChromBPNet models both use logits to represent the output profiles, this becomes

4 $$\ln ({{{{\rm{P}}}}}_{{{{\rm{bias}}}},i})+\ln ({{{{\rm{P}}}}}_{{{{\rm{biology}}}},i})=\ln ({{{{\rm{P}}}}}_{{{{\rm{experiment}}}},i})$$

in the implementation.

BPReveal implements this strategy using two BPNet-like models: One submodel, called the solo model, is pre-trained to predict just the experimental bias, and its weights are frozen during the training of the second submodel. The other submodel, called the residual model, has its output logits added to the output logits of the frozen bias model. These combined logits are then used in the standard loss function, Eq. (2).

A feature introduced by BPReveal is that the bias is transformed to better match the experimental data before its weights are frozen:

5 $${{{\rm{f}}}}(\ln ({{{{\rm{P}}}}}_{{{{\rm{bias}}}},i}))+\ln ({{{{\rm{P}}}}}_{{{{\rm{biology}}}},i})=\ln ({{{{\rm{P}}}}}_{{{{\rm{experiment}}}},i}),$$

where f is a simple function applied to all of the outputs from the bias model. For all of the models used in this paper, we used the function f(*x*) = *w*_1_*sigmoid(*w*_2 _*x* + *w*_3_) + *w*_4_. ChromBPNet, by comparison, does not transform the logits from the bias model, and applies a pre-computed scaling factor to the counts output. (We find that this nonlinear transformation does not offer a significant improvement over ChromBPNet for ATAC-seq data.) The full architecture is shown in Supplementary Fig. 2. BPReveal also includes features for motif scanning based on the original BPNet algorithm^3^.

### PISA

The calculation of PISA values ${{\mathbb{P}}}_{i\to j}$ for a particular genomic coordinate *j* is implemented in BPReveal in the following way. An input sequence is selected such that the prediction for base *j* will occur in the output of the model’s predicted profile. DeepSHAP is then used to partition the logit at position *j* among each input base *i* that is in the receptive field of base *j*. The references used for DeepSHAP are generated by shuffling the input sequence; an option is provided to perform kmer-preserving shuffles using the ushuffle algorithm^101^. Pseudocode for the PISA algorithm is presented in Box 1.

Box 1 Algorithm 1: PISA pseudocode In this pseudocode, i and j refer to genomic coordinates, and so shapVals[0] refers to ${{\mathbb{P}}}_{(\,{{{\boldsymbol{j}}}}-{{{\rm{buf}}}}) \to {{{\boldsymbol{j}}}}}$. For ease of calculation, we choose input sequences such that base *j* is at the leftmost output of the model. The value of the resulting array P holds, at position (i,j), the value of ${{\mathbb{P}}}_{i\to j}$.

buf = (inputLength - outputLength) // 2sequence = genome.fetch( j - buf, j + outputLength + buf)target = model.outputs[headID][0, strandID]explainer = shap.DeepExplainer( (model.input, target), ushuffle.shuffleOneHot)shapVals = explainer.shap_values(sequence)for i in range(j - buf, j + buf): P[i + j, j] = sum(shapVals[i - j + buf])

### Synthetic bias calculation

The ChromBPNet approach to removing experimental bias has a key limitation: it requires a model that has been trained to predict only bias. This is not straightforward for MNase-seq data. Therefore, a synthetic bias track for MNase-seq is derived using PISA in the following way.

Consider an ideal dataset giving the exact positions of nucleosomes by their $5^{\prime}$ and $3^{\prime}$ end points, with no experimental bias. These data could be used to train a two-strand model *I*; this model would have outputs ${I}^{5^{\prime} }$ and ${I}^{3^{\prime} }$. Consider a nucleosome that spans from base ${j}^{5^{\prime} }$ to ${j}^{3^{\prime} }$. If a base *i* contributes to this nucleosome’s position, then

6 $${\mathbb{P}}{({I}^{5^{\prime} })}_{i \to {j}^{5^{\prime} }}={\mathbb{P}}{({I}^{3^{\prime} })}_{i\to {j}^{3^{\prime} }}$$

where ${\mathbb{P}}{({I}^{5^{\prime} })}_{i\to {j}^{5^{\prime} }}$ represents the PISA contribution identified by ${I}^{5^{\prime} }$ from the base at position *i* (the x-axis in a PISA heatmap) onto the readout at position ${j}^{5^{\prime} }$ (the y-axis in the PISA heatmap). Because base *i*’s role to causing the $5^{\prime}$ endpoint to occur at base ${j}^{5^{\prime} }$ is the same as its role in causing the $3^{\prime}$ endpoint to occur at base ${j}^{3^{\prime} }$, these two contributions will be equal. (This assumption is not strictly true in the case of a partial nucleosome, where a base might not alter the position of a nucleosome dyad but would cause only one observed endpoint to shift. We have found that isolating regions with uniform nucleosome size does not affect bias model performance. (Supplementary Fig. 7)).

Now consider the case of real MNase-seq data that has been used to train model *M*. In this case, the experimental readout is not just based on the nucleosome positions (which would have been captured by the ideal model *I*), but also on the enzyme’s sequence bias that determines the observed endpoints. We refer to a model that captures this enzymatic bias as *B*. By Eq. (5), we have f(P_*B*,*j*_)*P_*I*,*j*_ = P_*M*,*j*_ for each base *j*. ${\mathbb{P}}{({M}^{5^{\prime} })}_{i\to {j}^{5^{\prime} }}$ still includes base *i*’s role in causing a nucleosome to have its $5^{\prime}$ endpoint at base ${j}^{5^{\prime} }$, but it also captures the role of base *i* in whether or not the MNase enzyme would stop at base ${j}^{5^{\prime} }$ due to bias. This introduces an asymmetry: by the nature of the MNase enzymatic bias, however, base *i* will only have an effect on the enzyme’s bias at ${j}^{5^{\prime} }$ if *i* is close to ${j}^{5^{\prime} }$. However, ${j}^{5^{\prime} }$ must be about 150 bp away from ${j}^{3^{\prime} }$ (since they represent opposite ends of the same nucleosome), and therefore if $i\approx {j}^{5^{\prime} }$ then ${\mathbb{P}}{({M}^{3^{\prime} })}_{i\to {j}^{3^{\prime} }}$ will be due to base *i*’s effect on nucleosome positioning and will have a minimal contribution from bias.

Therefore, for a base *i* close to ${j}^{5^{\prime} }$:

7 $${\mathbb{P}}{({M}^{5^{\prime} })}_{i\to {j}^{5^{\prime} }}	={\mathbb{P}}{({I}^{5^{\prime} })}_{i\to {j}^{5^{\prime} }}+{\mathbb{P}}{({B}^{5^{\prime} })}_{i\to {j}^{5^{\prime} }}\\ {\mathbb{P}}{({M}^{3^{\prime} })}_{i\to {j}^{3^{\prime} }}	={\mathbb{P}}{({I}^{3^{\prime} })}_{i\to {j}^{3^{\prime} }}+0$$

The use of addition here is justified by two points: First, as established by ChromBPNet^15^, attribution scores show that addition of logits leads to residual models (i.e., the models without bias) with no contribution from bias motifs. We show that PISA plots of our MNase residual model show insignificant bias-like density along the diagonal and that our synthetic bias model has density only along the diagonal. Second, since PISA values are (approximate) Shapley values, an effect that is due to a linear combination of two processes will yield Shapley values that are a linear combination of the two corresponding causes: for two models *I* and *B*,

8 $${\mathbb{P}}{(I+B)}_{i\to j}={\mathbb{P}}{(I)}_{i\to j}+{\mathbb{P}}{(B)}_{i\to j},$$

where *I* and *B* are the ideal and bias models, respectively. (and *I* + f(*B*) = *M* by Eq. (5))

By Eqs. (6) and (7), if $i\approx {j}^{5^{\prime} }$ then

9 $${\mathbb{P}}{({M}^{5^{\prime} })}_{i\to {j}^{5^{\prime} }}-{\mathbb{P}}{({M}^{3^{\prime} })}_{i\to {j}^{3^{\prime} }}={\mathbb{P}}{({B}^{5^{\prime} })}_{i\to {j}^{5^{\prime} }},$$

and if $i\approx {j}^{3^{\prime} }$ then

10 $${\mathbb{P}}{({M}^{3^{\prime} })}_{i\to {j}^{3^{\prime} }}-{\mathbb{P}}{({M}^{5^{\prime} })}_{i\to {j}^{5^{\prime} }}={\mathbb{P}}{({B}^{3^{\prime} })}_{i\to {j}^{3^{\prime} }},$$

which allows us to calculate ${\mathbb{P}}(B)$ from model *M*, which was trained only on the experimental MNase-seq.

In order to calculate the bias at position ${j}^{5^{\prime} }$ we must know where ${j}^{3^{\prime} }$ is. We would expect that the offset Δ between the two end points would be on the order of 150 bp (the length of DNA in a nucleosome). We determine the precise value of Δ by comparing the match between ${\mathbb{P}}{({M}^{5^{\prime} })}_{i\to j}$ and ${\mathbb{P}}{({M}^{3^{\prime} })}_{i\to (j+\Delta )}$ for a range of Δ values. Since the contribution of the bias model should be limited to regions where *i* ≈ *j*, we exclude a 60 bp window around the predicted outputs. In other words, we align the PISA heatmaps of the two strands of model *M* so that they match, except for the places where enzymatic bias is strong. We determined an optimal offset of 179 bp using this method, very close to the average fragment length in our MNase dataset. We find that the precise value of this offset is flexible, with good results for any value between 174 and 184 bp (Supplementary Fig. 13).

By the efficiency property of Shapley values, we can use the bias PISA values to reconstruct a bias profile track:

11 $${\sum}_{i\in {{{\rm{input}}}}}{\mathbb{P}}{({B}^{5^{\prime} })}_{i\to {j}^{5^{\prime} }}={B}_{{j}^{5^{\prime} }}^{5^{\prime} }-{B}_{{{{\rm{ref}}}},{j}^{5^{\prime} }}^{5^{\prime} }$$

where ${B}_{{j}^{5^{\prime} }}^{5^{\prime} }$ is the enzymatic bias on the $5^{\prime}$ end of reads starting at base ${j}^{5^{\prime} }$, ${B}_{{{{\rm{ref}}}},{j}^{5^{\prime} }}^{5^{\prime} }$ is the output of the model on shuffled copies of that same sequence, and input contains all bases that are in the receptive field of the model when it makes a prediction at base ${j}^{5^{\prime} }$. The average enzymatic bias on many random sequences (${B}_{ref}^{5^{\prime} }$) will be uniform, and since ${B}^{5^{\prime} }$ predicts logit values, a constant offset has no effect on the profile. (Eq. 11 is formulated in terms of the $5^{\prime}$ bias, but the same algorithm also applies to the $3^{\prime}$ case.) Therefore, by summing the rows of the synthetic bias PISA heatmap, we can derive a profile of MNase bias.

The essential process of shifting and subtracting is given in Box 2.

To get a genome-wide profile of MNase bias, we performed genome-wide PISA. In *S. cerevisiae*, this takes a few days on three Nvidia A100 GPUs. For a significantly larger genome, performing PISA on the entire genome is impractical. But since we train a model on the synthetic bias track, it is only necessary to produce enough synthetic bias data to train a model, and then that model can predict the bias genome-wide. In yeast, we have found that a high-quality bias model can be trained on as few as 1500 regions (Supplementary Fig. 7).

With this synthetic bias track in hand, a model can be trained on pure bias, which is then used to train a residual model à la ChromBPNet, thus learning the underlying biology that mixes with the bias to give rise to the experimental output.

Box 2 Algorithm 2: MNase bias extraction In this pseudocode, P3 and P5 are the PISA heatmaps for the $3^{\prime}$ and $5^{\prime}$ endpoints. strand specifies which head the bias is to be calculated for. A full implementation can be found in extractBiasBigwigs.py in the repository for this paper.

def getBias(P3, P5, strand): # P3 and P5 are the PISA heatmaps for the 3' # and 5' endpoints. Strand specifies which # head the bias is to be calculated for. # Move the 5' PISA heatmap to the left to # align with the 3' heatmap, or vice versa. if strand == "3'": P5Shift = shiftLeft(P5, 179) difference = P3 - P5Shift if strand == "5'": P3Shift = shiftRight(P3, 179) difference = P5 - P3Shift # The bias will come from near the diagonal, # so zero PISA values more than 13 bp from # the diagonal as we know they are not from # bias. maxJ, maxI = difference.shape for i in range(maxI): for j in range(maxJ): if abs(i - j) > 13: difference[j,i] = 0 # Use the efficiency property of Shapley # values to generate a bias track. biasLogits = sumRows(difference) bias = softmax(biasLogits) # Bias has shape (maxJ,) return bias

### Lean values and barrier identification

To identify barriers from a trained MNase-seq model, we systematically insert a nucleosome-positioning motif at every position *k* across the genome (or across regions of interest). For each motif insertion, we then calculate the lean value *L*^*k*^, which records whether the insertion at position *k* tends to perturb nucleosome positions more to the left or to the right. Barriers are the positions *k* where the lean values *L*^*k*^ transition from positive to negative.

For robustness, we performed the procedure with four different nucleosome-positioning motifs: *Abf1* (ATCATTGTGCACGG), *Rsc3* (CGCG), *Reb1* (GCCGGGTAAC), and *polyA* (AAAAAAA).

To obtain lean values *L* for each position *k*, the motif is inserted in the center of the output window of length *N* (the prediction window is shifted for each motif position *k*). We then calculate the mutation effect ${\Delta }_{i}^{k}$ at each position *i* of the output window:

12 $${\Delta }_{i}^{k}=| {{{{\rm{WTPred}}}}}_{i}-{{{{\rm{MutPred}}}}}_{i}^{k}|$$

where WTPred and MutPred^*k*^ are the predicted MNase profiles (midpoints, not $3^{\prime}$ or $5^{\prime}$ end points) for the wild-type sequence and the mutated sequence with the motif insertion at position *k*. WTPred, MutPred^*k*^ and Δ^*k*^ are vectors of length *N*.

Then we calculate the sum of the differences on the left side (${E}_{{{{\rm{left}}}}}^{k}$) and right side (${E}_{{{{\rm{right}}}}}^{k}$) of the injected motif at position *k*. Both are scalars with reads as units:

13 $${{{{\bf{E}}}}}_{{{{\rm{left}}}}}^{k}={\sum}_{i=0}^{k-10}{\Delta }_{i}^{k}$$

14 $${{{{\bf{E}}}}}_{{{{\rm{right}}}}}^{k}={\sum}_{i=k+10}^{N}{\Delta }_{i}^{k}$$

The lean value **L**^*k*^ (also a scalar) is then defined by:

15 $${{{{\bf{L}}}}}^{k}={{{{\bf{E}}}}}_{{{{\rm{left}}}}}^{k}-{{{{\bf{E}}}}}_{{{{\rm{right}}}}}^{k}$$

**L**^*k*^ is positive when the effect is primarily to the left of the injected motif and negative when the effect is to the right. After performing this calculation genome-wide, we have a value of **L** at each position in the genome (except for chromosome edges).

To identify barriers in the lean values, we applied two orthogonal methods to detect transitions from positive to negative lean values. The first method is based on counting positive and negative lean values. For a position *k*, if the lean values to the left of *k* are mostly left leaning and the lean values to the right of *k* are mostly right leaning, then position *k* represents a point where a motif’s influence flips from one domain to another:

16 $${{{\rm{sign}}}}(x)=\left\{\begin{array}{ll}1 \hfill & x > 0 \\ 0 \hfill & x=0 \\ -1 \hfill & x < 0\end{array}\right.$$

17 $${{{{\bf{S}}}}}_{{{{\rm{count}}}}}^{k}={\sum}_{i=k-500}^{k}{{{\rm{sign}}}}({L}^{i})-{\sum}_{i=k}^{k+500}{{{\rm{sign}}}}({L}^{i})$$

When *S*_count_ is maximal, the lean values flip from mostly positive to mostly negative, thus representing a candidate barrier.

Our second metric for identifying barriers, *S*_deriv_, is based on the derivative of the lean values. When the derivative is large and negative, the lean values are rapidly switching from left to right, potentially representing a barrier. To avoid tracking the nucleosome-scale oscillation observed in lean values, we added B as a filter:

18 $${{{{\bf{S}}}}}_{{{{\rm{deriv}}}}}^{k}=-\frac{{{{\rm{d}}}}}{{{{\rm{d}}}}k}{{{\rm{B}}}}{({{{\bf{L}}}})}^{k}$$

where B represents a 10th-order bidirectional Butterworth lowpass filter with a critical frequency of 1/200. When **S**_deriv_ is maximal, the lean values are rapidly decreasing, pointing to a candidate barrier.

To identify the maxima in the two score tracks **S**_count_ and **S**_deriv_, we applied scipy.signal.find_peaks. The height parameter was set to either the 90th percentile (derivative) or 70th percentile (counting) of the corresponding scores, the distance between peaks was at least 200 bp, and the prominence was set to twice the height.

To derive reproducible boundary elements from these candidate barriers, we combined those from both scores, **S**_count_ and **S**_deriv_, for each of the four inserted nucleosome positioning motifs. Boundary elements were called if they scored in at least seven out of the eight tests. The lean value tracks, along with the called barrier elements, are available in the insulators directory of the data deposited with this paper.

To calculate the enrichment of kmers around barriers, we identified all matches to the kmer or motif within 200 bp of all barriers, and then mean normalized the resulting histogram. To determine the spread of the distribution, we used scipy.optimize.curve_fit to match a Gaussian function with a constant offset:

19 $$\rho (x)=\alpha*\frac{{{{{\rm{e}}}}}^{\frac{-{(x-\mu )}^{2}}{2{\sigma }^{2}}}}{\sqrt{2\pi {\sigma }^{2}}}+\beta$$

where *ρ*(*x*) represents the frequency of occurrence of a particular motif or kmer at a distance *x* from a barrier. *μ* is the location of the peak relative to the barrier, *σ* is the standard deviation of the Gaussian function, *α* is an arbitrary scaling constant, and *β* is a constant offset to match the genomic background content of the motif or kmer.

### Genetic algorithm

To design novel sequences with a desired profile, we implemented a genetic algorithm (GA). This GA designs small sets of mutations that can be applied to an initial sequence in order to maximize a user-defined property of the prediction. Instead of operating directly on the sequence, our GA is given a pool of possible mutations (usually, every substitution, insertion, or deletion, with exceptions to prevent important sequence features from being altered). The optimization procedure then selects a subset (of user-defined size) of the possible mutations that maximize the fitness function. In this way, the algorithm can explore a wide array of possible mutations, including indels, but the edit distance from the WT sequence is limited to the size of the set of mutations. To design the mutation presented in Fig. 6, we allowed for three mutations and our fitness function minimized the nucleosome density in a window spanning chrII:431150-431250. Since we expected to test our mutations in vivo by using CRISPR/Cas9-mediated editing, we ran the GA once for every possible PAM site in the region, allowing mutations up to 50 bp downstream of the PAM site. We disallowed mutations inside the *Pho5* gene body, on the *Fkh2* motif, or on either of the *Pho4* motifs. Of the 51 runs (one for each PAM site), we manually selected a design that was predicted to remove the nucleosome on the high-affinity *Pho4* motif while leaving the other nucleosomes undisturbed.

### Sequence-based motif scanning

To identify motifs purely from sequence, we used Fimo^102^ from MEME suite version 5.5.8^85^, using the JASPAR databases appropriate for each organism (2022 vertebrates for mouse, 2020 insects for fly, and 2024 fungi for yeast)^103^. For regex-based scanning, we simply searched for instances of the motif in the genome sequence. All relevant code is included in the repository for this paper.

### Reporting summary

Further information on research design is available in the Nature Portfolio Reporting Summary linked to this article.

## Supplementary information

Supplementary Information

Description of Additional Supplementary Files

Supplementary Data 1

Supplementary Video 1

Reporting Summary

Transparent Peer Review file

## Data Availability

All analysis code, predicted tracks, models, configuration files, importance score tracks, and motifs have been deposited in Zenodo under accession code 20318019^104^ and the Stowers Original Data Repository[https://www.stowers.org/research/publications/libpb-2546]. Additionally, all training data, raw model predictions, raw PISA values, and raw importance scores used in this study have been deposited in the Stowers Original Data Repository[https://www.stowers.org/research/publications/libpb-2546]. The sequencing data for the *Pho5* site mutations generated in this study have been deposited in the NCBI SRA under accession GSE313524. The data used to train the OSKN model are available on the NCBI SRA under accession GSE137193. The data used to train the accessibility model and H3K27Ac model are available on the NCBI SRA under accession GSE218852. The data used to train the MNase model are available on the NCBI SRA under accession GSE153035. The Micro-C data used to illustrate boundaries are available on the NCBI SRA under accession GSE68016. The in vitro Micro-C data used to illustrate boundaries near *ISC1* and GPA2 are available on the NCBI SRA under accession GSE220647.

## Code availability

The BPReveal package has been uploaded to GitHub at https://github.com/mmtrebuchet/bpreveal under the GPL license (version 2 or later). The specific version used for this publication is archived on Zenodo under accession code 20384413^105^. All source code used for the analysis and modeling in this study is available at https://github.com/zeitlingerlab/bpreveal-manuscript under the GPL license (version 2 or later). This code has also been archived on Zenodo under accession code 20384039^106^.

Users are permitted to reuse, modify, and distribute the code in accordance with the terms of the GPL (version 2 or later).

## References

[1] Zeitlinger, J. et al. Perspective on recent developments and challenges in regulatory and systems genomics. *Bioinform. Adv.* **5**, vbaf106 (2025). 10.1093/bioadv/vbaf106 PMC12233095 40626041

[2] Kelley, D. R., Snoek, J. & Rinn, J. L. Basset: learning the regulatory code of the accessible genome with deep convolutional neural networks. *Genome Res.* **26**, 990–999 (2016). 27197224 10.1101/gr.200535.115 PMC4937568

[3] Avsec, v et al. Base-resolution models of transcription-factor binding reveal soft motif syntax. *Nat. Genet.* **53**, 354–366 (2021). 33603233 10.1038/s41588-021-00782-6 PMC8812996

[4] Avsec, v et al. Effective gene expression prediction from sequence by integrating long-range interactions. *Nat. Methods* **18**, 1196–1203 (2021). 34608324 10.1038/s41592-021-01252-x PMC8490152

[5] Zhou, J. & Troyanskaya, O. G. Predicting effects of noncoding variants with deep learning-based sequence model. *Nat. Methods* **12**, 931–934 (2015). 26301843 10.1038/nmeth.3547 PMC4768299

[6] Chen, K. M., Wong, A. K., Troyanskaya, O. G. & Zhou, J. A sequence-based global map of regulatory activity for deciphering human genetics. *Nat. Genet.* **54**, 940–949 (2022). 35817977 10.1038/s41588-022-01102-2 PMC9279145

[7] Penzar, D. et al. LegNet: a best-in-class deep learning model for short DNA regulatory regions. *Bioinformatics* **39**, (2023). 10.1093/bioinformatics/btad457 PMC10400376 37490428

[8] Linder, J., Srivastava, D., Yuan, H., Agarwal, V. & Kelley, D. R. Predicting RNA-seq coverage from DNA sequence as a unifying model of gene regulation. *Nat. Genet.* **57**, 949–961 (2025). 10.1038/s41588-024-02053-6 PMC11985352 39779956

[9] Cochran, K. et al. Dissecting the cis-regulatory syntax of transcription initiation with deep learning. 10.1101/2024.05.28.596138 (2024).

[10] He, A. Y. & Danko, C. G. Dissection of core promoter syntax through single nucleotide resolution modeling of transcription initiation. 10.1101/2024.03.13.583868 (2024).

[11] Dudnyk, K., Cai, D., Shi, C., Xu, J. & Zhou, J. Sequence basis of transcription initiation in the human genome. *Science.* **384**, eadj0116 (2024). 10.1126/science.adj0116 PMC11223672 38662817

[12] Avsec, Ž. et al. Advancing regulatory variant effect prediction with AlphaGenome. *Nature* **649**, 1206–1218 (2026). 10.1038/s41586-025-10014-0 PMC12851941 41606153

[13] Dalal, K. et al. Interpreting regulatory mechanisms of hippo signaling through a deep learning sequence model.*Cell Genomics.* 100821 (2025). 10.1016/j.xgen.2025.100821 PMC12008814 40174587

[14] Horton, C. A. et al. Short tandem repeats bind transcription factors to tune eukaryotic gene expression. *Science* **381**, eadd1250 (2023). 37733848 10.1126/science.add1250

[15] Pampari, A. et al. ChromBPNet: bias factorized, base-resolution deep learning models of chromatin accessibility reveal cis-regulatory sequence syntax, transcription factor footprints and regulatory variants. *BioRxiv*. 10.1101/2024.12.25.630221 (2025).

[16] Brennan, K. J. et al. Chromatin accessibility in the *Drosophila* embryo is determined by transcription factor pioneering and enhancer activation. *Dev. Cell* **58**, 1898–1916.e9 (2023). 37557175 10.1016/j.devcel.2023.07.007 PMC10592203

[17] de Almeida, B. P., Reiter, F., Pagani, M. & Stark, A. DeepSTARR predicts enhancer activity from DNA sequence and enables the de novo design of synthetic enhancers. *Nat. Genet.* **54**, 613–624 (2022). 35551305 10.1038/s41588-022-01048-5

[18] Cakiroglu, S. A., Steinhauser, S., Smith, J., Xing, W. & Luscombe, N. M. ChromWave: Deciphering the DNA-encoded competition between transcription factors and nucleosomes with deep neural networks. *BioRxiv*. 10.1101/2021.03.19.436198 (2021).

[19] Kelley, D. R. et al. Sequential regulatory activity prediction across chromosomes with convolutional neural networks. *Genome Res.* **28**, 739–750 (2018). 29588361 10.1101/gr.227819.117 PMC5932613

[20] Koo, P. K., Majdandzic, A., Ploenzke, M., Anand, P. & Paul, S. B. Global importance analysis: An interpretability method to quantify importance of genomic features in deep neural networks. *PLoS Comput. Biol.* **17**, e1008925 (2021). 33983921 10.1371/journal.pcbi.1008925 PMC8118286

[21] Novakovsky, G., Dexter, N., Libbrecht, M. W., Wasserman, W. W. & Mostafavi, S. Obtaining genetics insights from deep learning via explainable artificial intelligence. *Nat. Rev. Genet.* **24**, 125–137 (2023). 36192604 10.1038/s41576-022-00532-2

[22] Srivastava, D., Aydin, B., Mazzoni, E. O. & Mahony, S. An interpretable bimodal neural network characterizes the sequence and preexisting chromatin predictors of induced transcription factor binding. *Genome Biol.* **22**, 20 (2021). 33413545 10.1186/s13059-020-02218-6 PMC7788824

[23] Majdandzic, A., Rajesh, C. & Koo, P. K. Correcting gradient-based interpretations of deep neural networks for genomics. *Genome Biol.* **24**, 109 (2023). 37161475 10.1186/s13059-023-02956-3 PMC10169356

[24] van Hilten, A., Katz, S., Saccenti, E., Niessen, W. J. & Roshchupkin, G. V. Designing interpretable deep learning applications for functional genomics: a quantitative analysis. Brief, Bioinform. **25** (2024). 10.1093/bib/bbae449 PMC11410376 39293804

[25] Minnoye, L. et al. Cross-species analysis of enhancer logic using deep learning. *Genome Res.* **30**, 1815–1834 (2020). 32732264 10.1101/gr.260844.120 PMC7706731

[26] Routhier, E., Pierre, E., Khodabandelou, G. & Mozziconacci, J. Genome-wide prediction of DNA mutation effect on nucleosome positions for yeast synthetic genomics. *Genome Res.* **31**, 317–326 (2021). 33355297 10.1101/gr.264416.120 PMC7849406

[27] Bontonou, M. et al. Studying limits of explainability by integrated gradients for gene expression models. *arXiv: 2303.11336*. 10.48550/arxiv.2303.11336 (2023).

[28] Sundararajan, M., Taly, A., Yan, Q. Precup, D. & Teh, Y. W. (eds) *Axiomatic attribution for deep networks*. (eds Precup, D. & Teh, Y. W.) *Proceedings of the 34th International Conference on Machine Learning - Volume 70*, ICML’17, 3319–3328 (JMLR.org, 2017).

[29] Shrikumar, A., Greenside, P. & Kundaje, A. Learning important features through propagating activation differences. *arXiv: 1704.02685*10.48550/arxiv.1704.02685 (2017).

[30] Lundberg, S. M. & Lee, S.-I.Guyon, I. et al. (eds) * A unified approach to interpreting model predictions*. (eds Guyon, I.*et al*.) *Advances in Neural Information Processing Systems 30*, 4765–4774 (Curran Associates, Inc., 2017).

[31] Shrikumar, A. et al. TF-MoDISco v0.4.2.2-alpha: Technical note. *arXiv: 1811.00416*10.48550/arXiv.1811.00416 (2018).

[32] Martins, A. L., Walavalkar, N. M., Anderson, W. D., Zang, C. & Guertin, M. J. Universal correction of enzymatic sequence bias reveals molecular signatures of protein/DNA interactions. *Nucleic Acids Res.* **46**, e9 (2018). 29126307 10.1093/nar/gkx1053 PMC5778497

[33] Schreiber, J. tangermeme: A toolkit for understanding cis-regulatory logic using deep learning models. *BioRxiv*10.1101/2025.08.08.669296 (2025).

[34] Hörz, W. & Altenburger, W. Sequence specific cleavage of DNA by micrococcal nuclease. *Nucleic Acids Res.* **9**, 2643–2658 (1981). 7279658 10.1093/nar/9.12.2643 PMC326882

[35] Soufi, A., Donahue, G. & Zaret, K. S. Facilitators and impediments of the pluripotency reprogramming factors’ initial engagement with the genome. *Cell* **151**, 994–1004 (2012). 23159369 10.1016/j.cell.2012.09.045 PMC3508134

[36] Maresca, M. et al. Pioneer activity distinguishes activating from non-activating SOX2 binding sites. * EMBO J.* **42**, e113150 (2023). 37691488 10.15252/embj.2022113150 PMC10577566

[37] Weilert, M. et al. Widespread low-affinity motifs enhance chromatin accessibility and regulatory potential in mESCs. *bioRxiv*10.1101/2025.11.18.685822 (2025).

[38] Liang, H.-L. et al. The zinc-finger protein zelda is a key activator of the early zygotic genome in drosophila. *Nature* **456**, 400–403 (2008). 18931655 10.1038/nature07388 PMC2597674

[39] Sun, Y. et al. Zelda overcomes the high intrinsic nucleosome barrier at enhancers during drosophila zygotic genome activation. *Genome Res.* **25**, 1703–1714 (2015). 26335633 10.1101/gr.192542.115 PMC4617966

[40] Schulz, K. N. et al. Zelda is differentially required for chromatin accessibility, transcription factor binding, and gene expression in the early drosophila embryo. *Genome Res.* **25**, 1715–1726 (2015). 26335634 10.1101/gr.192682.115 PMC4617967

[41] Yáñez Cuna, J. O. et al. Dissection of thousands of cell type-specific enhancers identifies dinucleotide repeat motifs as general enhancer features. *Genome Res.* **24**, 1147–1156 (2014). 24714811 10.1101/gr.169243.113 PMC4079970

[42] Koenecke, N., Johnston, J., Gaertner, B., Natarajan, M. & Zeitlinger, J. Genome-wide identification of Drosophila dorso-ventral enhancers by differential histone acetylation analysis. *Genome Biol.* **17**, 196 (2016). 10.1186/s13059-016-1057-2 PMC5037609 27678375

[43] Li, X.-Y., Harrison, M. M., Villalta, J. E., Kaplan, T. & Eisen, M. B. Establishment of regions of genomic activity during the Drosophila maternal to zygotic transition. *eLife* **3**, e03737 (2014). 10.7554/eLife.03737 PMC4358338 25313869

[44] Stampfel, G. et al. Transcriptional regulators form diverse groups with context-dependent regulatory functions. *Nature* **528**, 147–151 (2015). 26550828 10.1038/nature15545

[45] Marsh, A. J. et al. Catalytic-dependent and independent functions of the histone acetyltransferase CBP promote pioneer-factor-mediated zygotic genome activation. *Mol Cell.* **85**, 2409–2424.e8 (2025). 10.1016/j.molcel.2025.05.009 PMC12181052 40441155

[46] Green, B., Bouchier, C., Fairhead, C., Craig, N. L. & Cormack, B. P. Insertion site preference of Mu, Tn5, and Tn7 transposons. *Mob. DNA* **3**, 3 (2012). 22313799 10.1186/1759-8753-3-3 PMC3292447

[47] Dingwall, C., Lomonossoff, G. P. & Laskey, R. A. High sequence specificity of micrococcal nuclease. *Nucleic Acids Res.* **9**, 2659–2673 (1981). 6269057 10.1093/nar/9.12.2659 PMC326883

[48] Wolff, M. R., Schmid, A., Korber, P. & Gerland, U. Effective dynamics of nucleosome configurations at the yeast PHO5 promoter. *eLife* **10**, (2021). 10.7554/eLife.58394 PMC8004102 33666171

[49] Wang, X., Bai, L., Bryant, G. O. & Ptashne, M. Nucleosomes and the accessibility problem. *Trends Genet.* **27**, 487–492 (2011). 22019336 10.1016/j.tig.2011.09.001

[50] Segal, E. et al. A genomic code for nucleosome positioning. *Nature* **442**, 772–778 (2006). 16862119 10.1038/nature04979 PMC2623244

[51] Begley, V. et al. Xrn1 influence on gene transcription results from the combination of general effects on elongating RNA pol II and gene-specific chromatin configuration. *RNA Biol.* **18**, 1310–1323 (2021). 33138675 10.1080/15476286.2020.1845504 PMC8354610

[52] Peckham, H. E. et al. Nucleosome positioning signals in genomic DNA. *Genome Res.* **17**, 1170–1177 (2007). 17620451 10.1101/gr.6101007 PMC1933512

[53] Cui, F., Chen, L., LoVerso, P. R. & Zhurkin, V. B. Prediction of nucleosome rotational positioning in yeast and human genomes based on sequence-dependent DNA anisotropy. *BMC Bioinforma.* **15**, 313 (2014). 10.1186/1471-2105-15-313 PMC4261538 25244936

[54] Liu, G. et al. A deformation energy-based model for predicting nucleosome dyads and occupancy. *Sci. Rep.* **6**, 24133 (2016). 27053067 10.1038/srep24133 PMC4823781

[55] Basu, A. et al. Measuring DNA mechanics on the genome scale. *Nature* **589**, 462–467 (2021). 33328628 10.1038/s41586-020-03052-3 PMC7855230

[56] Gutiérrez, G. et al. Subtracting the sequence bias from partially digested MNase-seq data reveals a general contribution of TFIIS to nucleosome positioning. *Epigenet. Chromat.* **10**, 58 (2017). 10.1186/s13072-017-0165-x PMC5719526 29212533

[57] Tillo, D. & Hughes, T. R. G+C content dominates intrinsic nucleosome occupancy. *BMC Bioinforma.* **10**, 442 (2009). 10.1186/1471-2105-10-442 PMC2808325 20028554

[58] Kaplan, N. et al. The DNA-encoded nucleosome organization of a eukaryotic genome. *Nature* **458**, 362–366 (2009). 19092803 10.1038/nature07667 PMC2658732

[59] Oberbeckmann, E. et al. Genome information processing by the INO80 chromatin remodeler positions nucleosomes. *Nat. Commun.* **12**, 3231 (2021). 34050142 10.1038/s41467-021-23016-z PMC8163841

[60] Sala, A. et al. An integrated machine-learning model to predict nucleosome architecture. *Nucleic Acids Res.* **52**, 10132–10143 (2024). 39162225 10.1093/nar/gkae689 PMC11417389

[61] Badis, G. et al. A library of yeast transcription factor motifs reveals a widespread function for Rsc3 in targeting nucleosome exclusion at promoters. *Mol. Cell* **32**, 878–887 (2008). 19111667 10.1016/j.molcel.2008.11.020 PMC2743730

[62] Floer, M. et al. A RSC/nucleosome complex determines chromatin architecture and facilitates activator binding. *Cell* **141**, 407–418 (2010). 20434983 10.1016/j.cell.2010.03.048 PMC3032599

[63] Barnes, T. & Korber, P. The active mechanism of nucleosome depletion by poly(dA:dT) tracts in vivo. *Int. J. Mol. Sci.* **22**, 8233 (2021). 10.3390/ijms22158233 PMC8347975 34360997

[64] Raveh-Sadka, T. et al. Manipulating nucleosome disfavoring sequences allows fine-tune regulation of gene expression in yeast. *Nat. Genet.* **44**, 743–750 (2012). 22634752 10.1038/ng.2305

[65] Anderson, J. D. & Widom, J. Poly(dA-dT) promoter elements increase the equilibrium accessibility of nucleosomal DNA target sites. *Mol. Cell. Biol.* **21**, 3830–3839 (2001). 11340174 10.1128/MCB.21.11.3830-3839.2001 PMC87046

[66] Thåström, A. et al. Sequence motifs and free energies of selected natural and non-natural nucleosome positioning DNA sequences. *J. Mol. Biol.* **288**, 213–229 (1999). 10329138 10.1006/jmbi.1999.2686

[67] Lancrey, A. et al. Nucleosome positioning on large tandem DNA repeats of the ‘601’ sequence engineered in saccharomyces cerevisiae. *J. Mol. Biol.* **434**, 167497 (2022). 35189129 10.1016/j.jmb.2022.167497

[68] Segal, E. & Widom, J. What controls nucleosome positions? *Trends Genet.* **25**, 335–343 (2009). 19596482 10.1016/j.tig.2009.06.002 PMC2810357

[69] Struhl, K. & Segal, E. Determinants of nucleosome positioning. *Nat. Struct. Mol. Biol.* **20**, 267–273 (2013). 23463311 10.1038/nsmb.2506 PMC3740156

[70] Zhang, Y. et al. Intrinsic histone-DNA interactions are not the major determinant of nucleosome positions in vivo. *Nat. Struct. Mol. Biol.* **16**, 847–852 (2009). 19620965 10.1038/nsmb.1636 PMC2823114

[71] Kaplan, N. et al. Nucleosome sequence preferences influence in vivo nucleosome organization. *Nat. Struct. Mol. Biol.* **17**, 918–920 (2010). 20683473 10.1038/nsmb0810-918 PMC2969171

[72] Zhang, Y. et al. Evidence against a genomic code for nucleosome positioning. reply to “nucleosome sequence preferences influence in vivo nucleosome organization”. *Nat. Struct. Mol. Biol.* **17**, 920–923 (2010). 20683474 10.1038/nsmb0810-920 PMC4742340

[73] Lorch, Y., Maier-Davis, B. & Kornberg, R. D. Role of DNA sequence in chromatin remodeling and the formation of nucleosome-free regions. *Genes Dev.* **28**, 2492–2497 (2014). 25403179 10.1101/gad.250704.114 PMC4233242

[74] Hsieh, T.-H. S. et al. Mapping nucleosome resolution chromosome folding in yeast by micro-c. *Cell* **162**, 108–119 (2015). 26119342 10.1016/j.cell.2015.05.048 PMC4509605

[75] Wiese, O., Marenduzzo, D. & Brackley, C. A. Nucleosome positions alone can be used to predict domains in yeast chromosomes. *Proc. Natl. Acad. Sci. USA* **116**, 17307–17315 (2019). 31416914 10.1073/pnas.1817829116 PMC6717315

[76] Oberbeckmann, E., Quililan, K., Cramer, P. & Oudelaar, A. M. In vitro reconstitution of chromatin domains shows a role for nucleosome positioning in 3D genome organization. *Nat. Genet.* **56**, 483–492 (2024). 38291333 10.1038/s41588-023-01649-8 PMC10937381

[77] Taskiran, I. I. et al. Cell-type-directed design of synthetic enhancers. *Nature* **626**, 212–220 (2024). 38086419 10.1038/s41586-023-06936-2 PMC10830415

[78] Routhier, E. et al. In silico design of DNA sequences for in vivo nucleosome positioning. *Nucleic Acids Res.* **52**, 6802–6810 (2024). 38828788 10.1093/nar/gkae468 PMC11229325

[79] Korber, P. & Barbaric, S. The yeast PHO5 promoter: from single locus to systems biology of a paradigm for gene regulation through chromatin. *Nucleic Acids Res.* **42**, 10888–10902 (2014). 25190457 10.1093/nar/gku784 PMC4176169

[80] Lam, F. H., Steger, D. J. & O’Shea, E. K. Chromatin decouples promoter threshold from dynamic range. *Nature* **453**, 246–250 (2008). 18418379 10.1038/nature06867. PMC2435410

[81] Brown, C. R., Mao, C., Falkovskaia, E., Jurica, M. S. & Boeger, H. Linking stochastic fluctuations in chromatin structure and gene expression. *PLoS Biol.* **11**, e1001621 (2013). 23940458 10.1371/journal.pbio.1001621 PMC3735467

[82] Vogel, K., Hörz, W. & Hinnen, A. The two positively acting regulatory proteins PHO2 and PHO4 physically interact with PHO5 upstream activation regions. *Mol. Cell. Biol.* **9**, 2050–2057 (1989). 2664469 10.1128/mcb.9.5.2050-2057.1989 PMC362998

[83] Rudolph, H. & Hinnen, A. The yeast PHO5 promoter: phosphate-control elements and sequences mediating mRNA start-site selection. *Proc. Natl. Acad. Sci. USA* **84**, 1340–1344 (1987). 2881299 10.1073/pnas.84.5.1340 PMC304424

[84] Rajkumar, A. S., Dénervaud, N. & Maerkl, S. J. Mapping the fine structure of a eukaryotic promoter input-output function. *Nat. Genet.* **45**, 1207–1215 (2013). 23955598 10.1038/ng.2729

[85] Bailey, T. L., Johnson, J., Grant, C. E. & Noble, W. S. The MEME suite. *Nucleic Acids Res.* **43**, W39–49 (2015). 25953851 10.1093/nar/gkv416 PMC4489269

[86] Avsec, Z. BPNet manuscript data 1 [Data set]. Zenodo. 10.5281/zenodo.4294904 (2019).

[87] Avsec, Z. BPNet manuscript data 2 [Data set]. Zenodo. 10.5281/zenodo.3371216 (2019).

[88] Durbrow, K. et al. ncbi/sra-tools. https://github.com/ncbi/sra-tools (2026).

[89] Langmead, B., Wilks, C., Antonescu, V. & Charles, R. Scaling read aligners to hundreds of threads on general-purpose processors. *Bioinformatics* **35**, 421–432 (2019). 30020410 10.1093/bioinformatics/bty648 PMC6361242

[90] Aguilar, R. R., Shen, Z.-J. & Tyler, J. K. A simple, improved method for scarless genome editing of budding yeast using CRISPR-cas9. *Methods Protocols* **5**, 79 (2022). 10.3390/mps5050079 PMC9607540 36287051

[91] McKnight, L. E. et al. Rapid and inexpensive preparation of genome-wide nucleosome footprints from model and non-model organisms. *STAR Protoc.* **2**, 100486 (2021). 34041500 10.1016/j.xpro.2021.100486 PMC8141940

[92] Quinlan, A. R. & Hall, I. M. BEDTools: a flexible suite of utilities for comparing genomic features. *Bioinformatics* **26**, 841–842 (2010). 20110278 10.1093/bioinformatics/btq033 PMC2832824

[93] Chollet, F. et al. Keras. https://keras.io (2015).

[94] Harris, C. R. et al. Array programming with NumPy. *Nature* **585**, 357–362 (2020). 32939066 10.1038/s41586-020-2649-2 PMC7759461

[95] Dale, R. K., Pedersen, B. S. & Quinlan, A. R. Pybedtools: a flexible python library for manipulating genomic datasets and annotations. *Bioinformatics* **27**, 3423–3424 (2011). 21949271 10.1093/bioinformatics/btr539 PMC3232365

[96] Ryan, D. et al. deeptools/pyBigWig: 0.3.24 (0.3.24). Zenodo. 10.5281/zenodo.14679776 (2025).

[97] Heger, A. et al. pysam-developers/pysam. https://github.com/pysam-developers/pysam (2026).

[98] Virtanen, P. et al. SciPy 1.0: Fundamental Algorithms for Scientific Computing in Python. *Nat. Methods* **17**, 261–272 (2020). 32015543 10.1038/s41592-019-0686-2 PMC7056644

[99] TensorFlow Developers. TensorFlow (v2.20.0). Zenodo. 10.5281/zenodo.16852354 (2025).

[100] Li, H. et al. The sequence alignment/map format and SAMtools. *Bioinformatics* **25**, 2078–2079 (2009). 19505943 10.1093/bioinformatics/btp352 PMC2723002

[101] Jiang, M., Anderson, J., Gillespie, J. & Mayne, M. uShuffle: a useful tool for shuffling biological sequences while preserving the k-let counts. *BMC Bioinforma.* **9**, 192 (2008). 10.1186/1471-2105-9-192 PMC2375906 18405375

[102] Grant, C. E., Bailey, T. L. & Noble, W. S. FIMO: scanning for occurrences of a given motif. *Bioinformatics* **27**, 1017–1018 (2011). 21330290 10.1093/bioinformatics/btr064 PMC3065696

[103] Rauluseviciute, I. et al. JASPAR 2024: 20th anniversary of the open-access database of transcription factor binding profiles. *Nucleic Acids Res.* **52**, D174–D182 (2024). 37962376 10.1093/nar/gkad1059 PMC10767809

[104] McAnany, C. et al. Positional Interpretation of Cis-Regulatory Code and Nucleosome Organization with Deep Learning Models: Data, models, and code [Data set]. Zenodo. 10.5281/zenodo.20318019 (2026). 10.1038/s41467-026-74807-1 PMC13469612 42401553

[105] McAnany, C., Moeller, P. & Weilert, M. mmtrebuchet/bpreveal: Zenodo release (v5.2.2-zenodo). Zenodo. 10.5281/zenodo.20384113 (2026).

[106] McAnany, C. mmtrebuchet/bpreveal-manuscript: Archive for paper (paper). Zenodo. 10.5281/zenodo.20384039 (2026).

