# Supplementary material for: Positional interpretation of cis-regulatory code and nucleosome organization with deep learning models
Source: Nat Commun. 2026 Jul 4;17:8326. doi: 10.1038/s41467-026-74807-1 (PMC13469612; doi:10.1038/s41467-026-74807-1)
Supplement: Supplementary file 1 [file 41467_2026_74807_MOESM1_ESM.pdf]

Supplementary Information for Positional Interpretation of Cis-Regulatory Code and Nucleosome Organization with Deep Learning Models

Charles E. McAnany[1], Melanie Weilert[1], Grishma Mehta[1,2], Fahad Kamulegeya[1], Jennifer M. Gardner[1], Jacob Schreiber[3,4], Anshul Kundaje[5,6], Julia Zeitlinger[1,7*]

[1*]Stowers Institute for Medical Research, Kansas City, MO, 64110, USA.
[2]Indian Institute of Science Education & Research, Pashan, Pune 411008, India.
[3]Research Institute of Molecular Pathology, Vienna BioCenter, Vienna, 1030, Austria.
[4]Department of Genomics and Computational Biology, UMass Chan Medical School, Worcester, MA, 01655, USA.
[5]Department of Genetics, Stanford University, Palo Alto, CA, 94304, USA.
[6]Department of Computer Science, Stanford University, Palo Alto, CA, 94304, USA.
[7]Department of Pathology & Laboratory Medicine, The University of Kansas Medical Center, Kansas City, KS, 66160, USA.

*Corresponding author(s). E-mail(s): jbz@stowers.org;

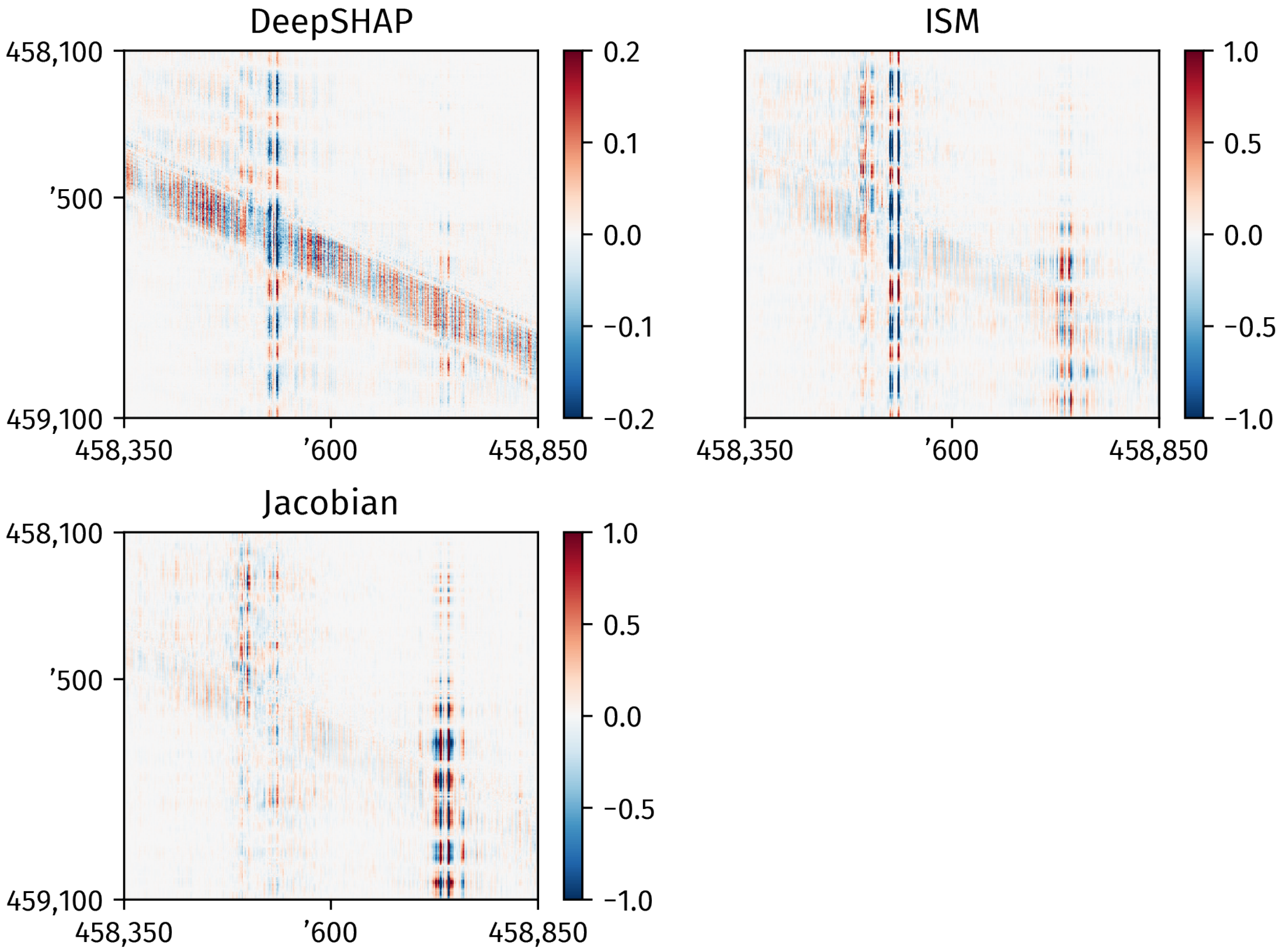

Supplementary Figure 1 PISA plots can be generated using saturation mutagenesis. We show three ways of generating PISA heatmaps for a locus. DeepSHAP is the method used in this paper, and is based on Shapley values. In silico mutagenesis (ISM) uses saturation mutagenesis to determine the effect of every possible mutation. Qualitatively, ISM detects the same motif as the DeepSHAP implementation, though shows less density in the nucleosome body outside of the motif instances. We believe this is a result of the distributed nature of the intrinsic component of nucleosome positioning. Any single mutation has a small effect (low ISM score), but the intrinsic component as a whole has a large role in nucleosome positioning and so the Shapley value assigned to those bases reflects their overall role. Directly calculating the derivative of the output with respect to the input is known to be hazardous in deep learning, and PISA is no exception. Calculating a Jacobian, while computationally cheap, provides low-quality interpretations. We do not recommend it.

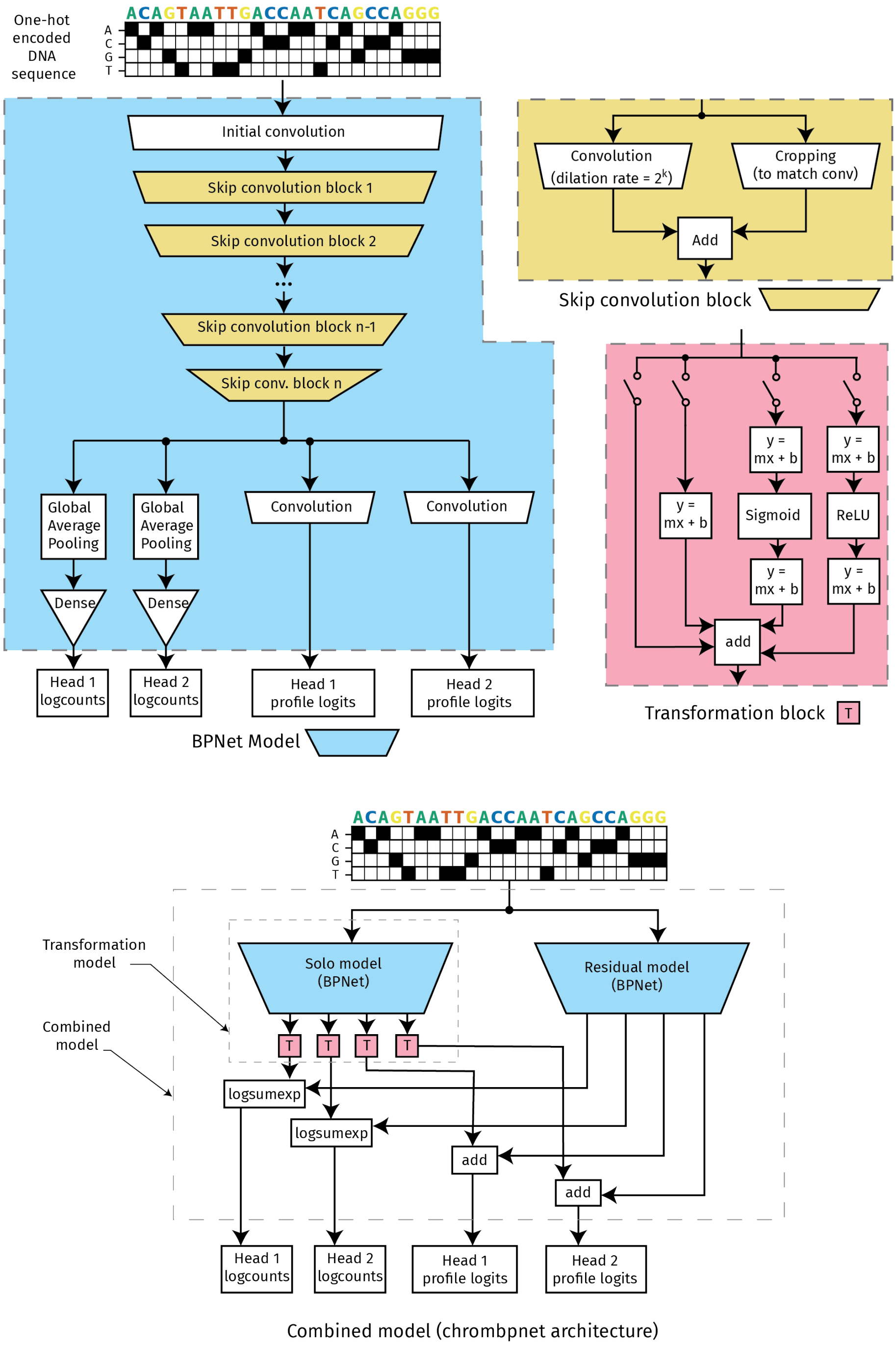

Supplementary Figure 2 BPReveal architecture. (blue) A typical BPNet-style model with two output heads. The input to a BPReveal model is one-hot encoded DNA sequence. The core of the model is a stack of dilated convolutional layers with skip connections (yellow trapezoids). (yellow) The architecture of a dilated convolutional layer with skip connection. (red) The architecture of the transformation model, used to regress the predictions of a solo (i.e., bias) model to better match experimental data. (bottom) A complete BPReveal model incorporating ChromBPNet-style bias correction. The solo model is pre-trained on enzymatic bias, and then its weights are frozen. One transformation model is applied to each output of the solo model, and the weights in the transformation model are trained on the experimental data. Finally, the weights of both the solo and transformation model are frozen, and a new residual model is trained to learn the experimental data.

Supplementary Table 1 Performance comparison for OSKN model We trained a BPReveal model using the same data as were used in Avsec *et al*[1]. We assess the quality of a model's predictions against an experimental dataset using two metrics. The profile Jensen-Shannon divergence measures the similarity between the predicted and observed data at high resolution, while the Spearman correlation of the counts measures the performance of the model over the entire output window. Overall, BPReveal performs similarly to the previous state of the art in TF binding models.

		Counts Spearman correlation (higher is better)					
		Training set			Validation set		
TF	strand	BPReveal	Avsec	Δ	BPReveal	Avsec	Δ
Oct4	positive	0.576	0.469	**0.107**	0.512	0.429	**0.083**
Oct4	negative	0.577	0.464	**0.113**	0.513	0.425	**0.088**
Sox2	positive	0.496	0.491	**0.005**	0.427	0.450	-0.024
Sox2	negative	0.500	0.488	**0.012**	0.424	0.443	-0.019
Klf4	positive	0.684	0.526	**0.159**	0.646	0.488	**0.158**
Klf4	negative	0.684	0.529	**0.154**	0.647	0.491	**0.156**
Nanog	positive	0.616	0.661	-0.045	0.579	0.642	-0.064
Nanog	negative	0.618	0.665	-0.047	0.580	0.648	-0.068

		Profile Jensen-Shannon divergence (lower is better)					
		Training set			Validation set		
TF	strand	BPReveal	Avsec	Δ	BPReveal	Avsec	Δ
Oct4	positive	0.696	0.680	0.017	0.696	0.679	0.018
Oct4	negative	0.697	0.679	0.018	0.697	0.679	0.018
Sox2	positive	0.758	0.753	0.005	0.757	0.751	0.006
Sox2	negative	0.758	0.752	0.006	0.758	0.752	0.006
Klf4	positive	0.675	0.642	0.033	0.677	0.644	0.033
Klf4	negative	0.675	0.640	0.034	0.677	0.643	0.034
Nanog	positive	0.676	0.675	0.001	0.676	0.675	0.001
Nanog	negative	0.676	0.675	0.001	0.677	0.674	0.003

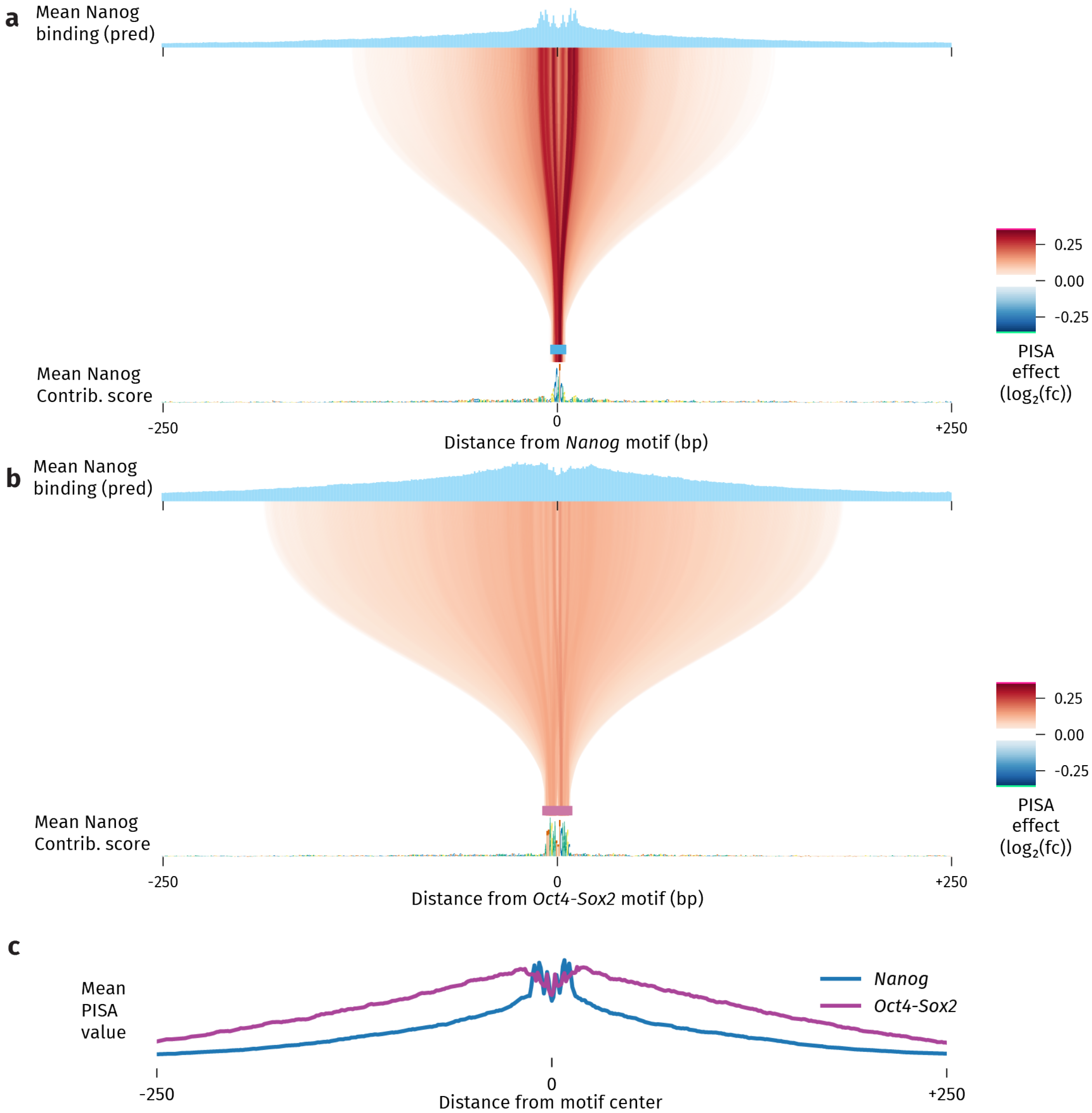

Supplementary Figure 3 The positional effects of *Oct4-Sox2* and *Nanog* are consistent across many instances. We performed PISA on 400 *Oct4-Sox2* motifs and 400 *Nanog* motifs identified by our Nanog binding model. (a) The average PISA plot for all *Nanog* motifs shows a strong effect close to the motif, associated with sharp spikes of Nanog binding. (b) *Oct4-Sox2* shows a broader character reflective of its role as a pioneer. Nanog binding is enhanced in a broad region around the *Oct4-Sox2* motif. (c) The average PISA effects from the two motifs. While the effects are similar in magnitude, their extents are clearly very different.

Supplementary Table 2 Performance comparison for ATAC-seq model. We trained a BPReveal model on the same data used in Brennan *et al*[2], and assessed the two models' accuracy using the same metrics as in Supplementary Table 1. Our BPReveal model uses essentially the same architecture as ChromBPNet, and so the results are unsurprisingly very similar.

Counts Spearman correlation (higher is better)

Training set			Validation set		
BPReveal	Brennan	Δ	BPReveal	Brennan	Δ
0.753	0.759	-0.007	0.634	0.590	**0.043**

Profile Jensen-Shannon divergence (lower is better)

Training set			Validation set		
BPReveal	Brennan	Δ	BPReveal	Brennan	Δ
0.277	0.289	**-0.012**	0.291	0.308	**-0.017**

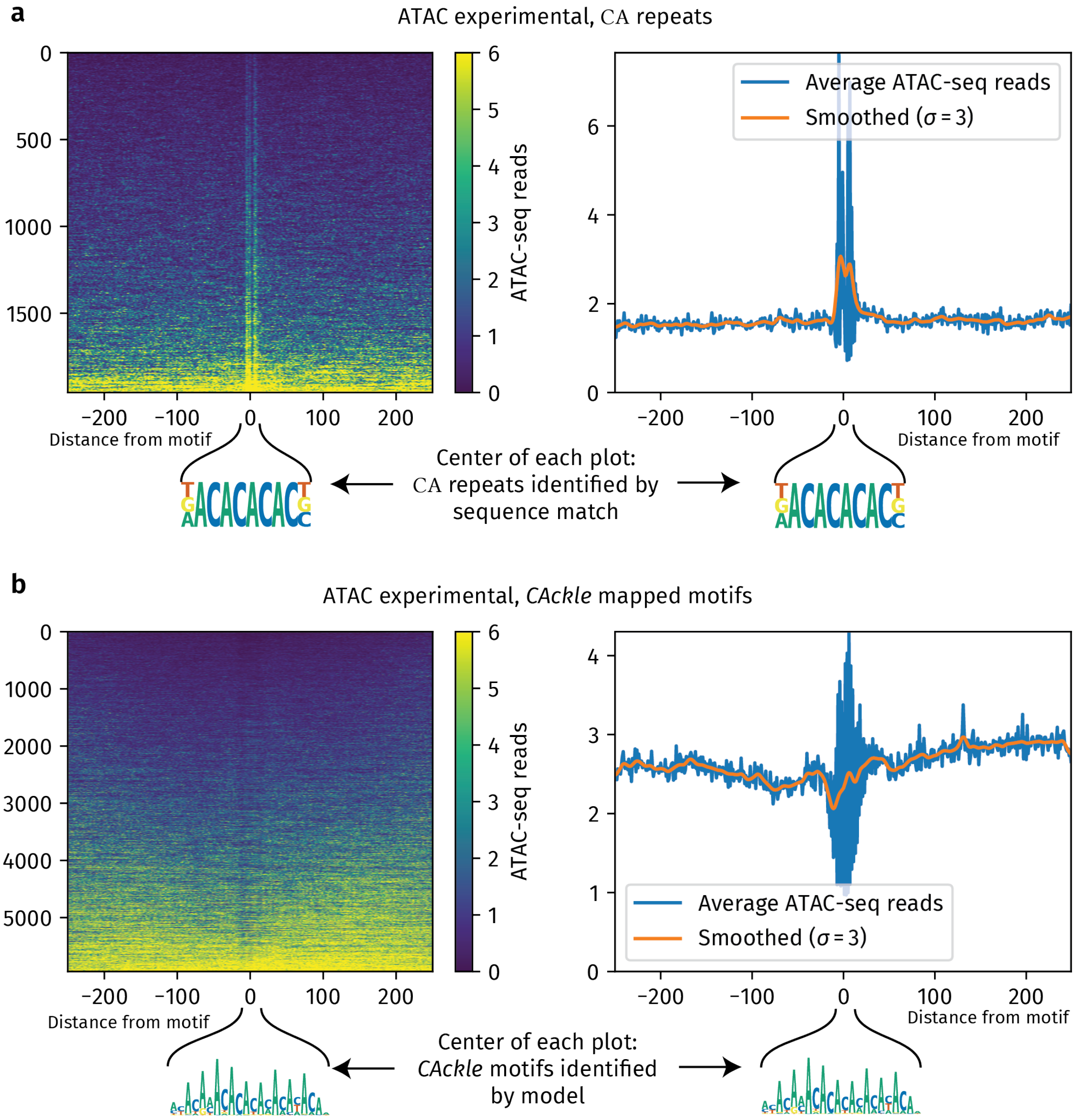

Supplementary Figure 4 The *CAckle* motif contributes to Tn5 enzymatic bias, but can also create a depleted footprint. We show heatmaps and metapeaks of experimental ATAC-seq data at `CA` repeats. (a) Mapping `CA` repeats by sequence alone shows a pronounced spike in ATAC-seq reads at the site of the `CA` repeat. (left) A heatmap of ATAC-seq profiles at `CA` repeats, ordered by total ATAC-seq reads in the region. (right) An average profile of the of the `CA` repeat instances shows that many ATAC-seq reads occur at the site of the repeat. (b) By using model-derived motifs instead of just sequence match, we see that *CAckle* motif instances can cause a local dip in ATAC-seq reads at some regions. A heatmap of ATAC-seq profiles at mapped *CAckle* motifs (left) and an average profile over all mapped motif instances (right) show a slight dip in ATAC-seq reads at the *CAckle* motif. For ease of visualization, a $\sigma = 1$ Gaussian filter has been applied to both heatmaps. The orange traces in the average profiles are smoothed with a $\sigma = 3$ Gaussian filter applied to the (unsmoothed) blue average profile traces.

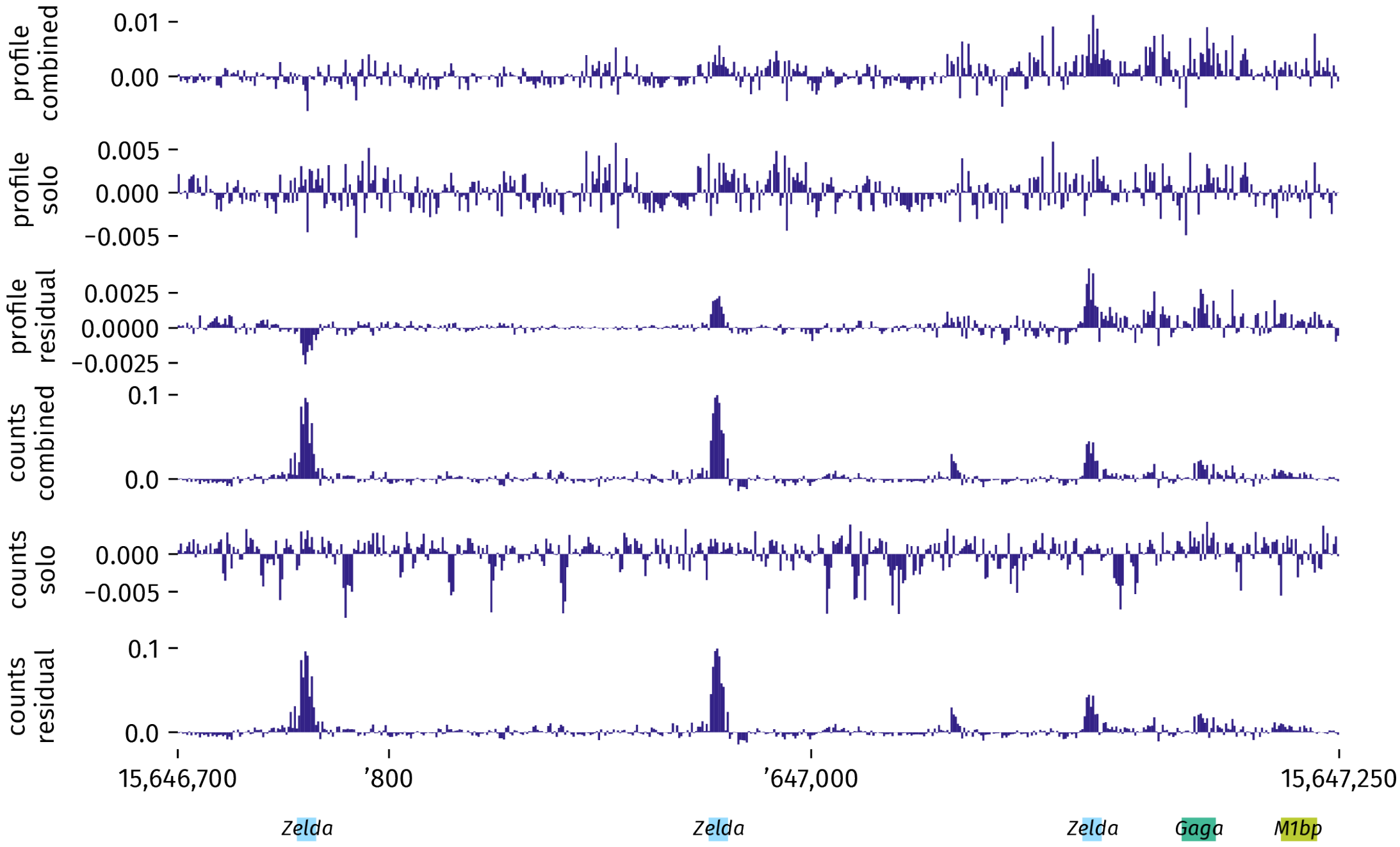

Supplementary Figure 5 Importance score comparison for ATAC bias correction. The ChromBPNet bias correction strategy leads to a dramatic improvement in profile contribution scores, but does not improve counts contribution scores. We show importance score tracks at the *sog* locus for the combined model (which predicts the actual experimental data, including bias), the solo model (which only predicts bias), and the residual model (which does not include bias). The profile contribution scores for the combined and solo model both show a great deal of noise due to bias, whereas the bias-corrected residual model shows much clearer peaks at the three *Zelda* motifs. The enzymatic bias encountered in ATAC-seq has a much smaller effect on the total counts prediction, such that the combined model already clearly shows the three *Zelda* motifs; the counts contribution scores from the residual model are the same as for the combined model in this case.

Supplementary Table 3 Performance data for BPReveal MNase model. These statistics are based on the 5′ endpoint predictions from the model trained on the data from Begley *et al*[3]. (Metrics for the 3′ endpoint predictions (not shown) are within 1% of the values in this table.)

Metric	Training set	Validation set
Profile Jensen-Shannon divergence (lower is better)	0.235	0.260
Profile Pearson (higher is better)	0.856	0.815
Counts Pearson (higher is better)	0.717	0.626
Counts Spearman (higher is better)	0.711	0.612

Supplementary Table 4 Performance comparison for Routhier MNase model. A BPReveal model was trained on the same data as the model in Routhier *et al*[4], and the BPReveal model performs as well or better than the previous state of the art. The correlation metric for the Routhier model is base-by-base for an entire region of a chromosome, and therefore there is no distinction between a profile and counts metric. Our metrics here are for the validation set.

Metric	BPReveal	Routhier
Counts Pearson (higher is better)	**0.770**	0.68
Profile Pearson (higher is better)	**0.700**	0.68

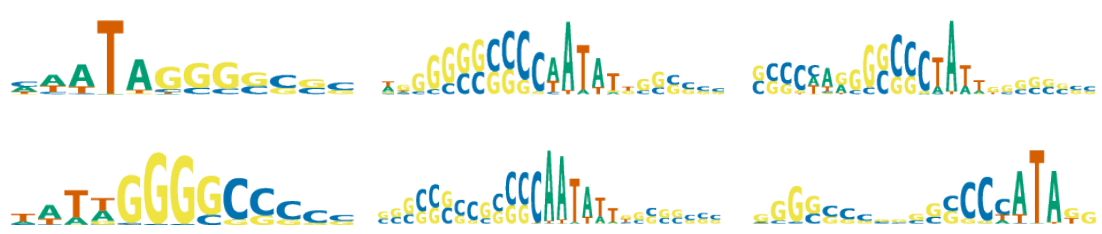

Supplementary Figure 6 Motifs identified by MNase bias model. Motifs identified from profile contribution scores for our MNase bias model show a transition from `AT`-rich to `CG`-rich regions, consistent with the well-characterized bias of the MNase enzyme. The six motifs shown here are the six most frequent seqlets identified by TF-MoDISco; none of the 40 motifs it identified this model resembled a biologically-relevant motif.

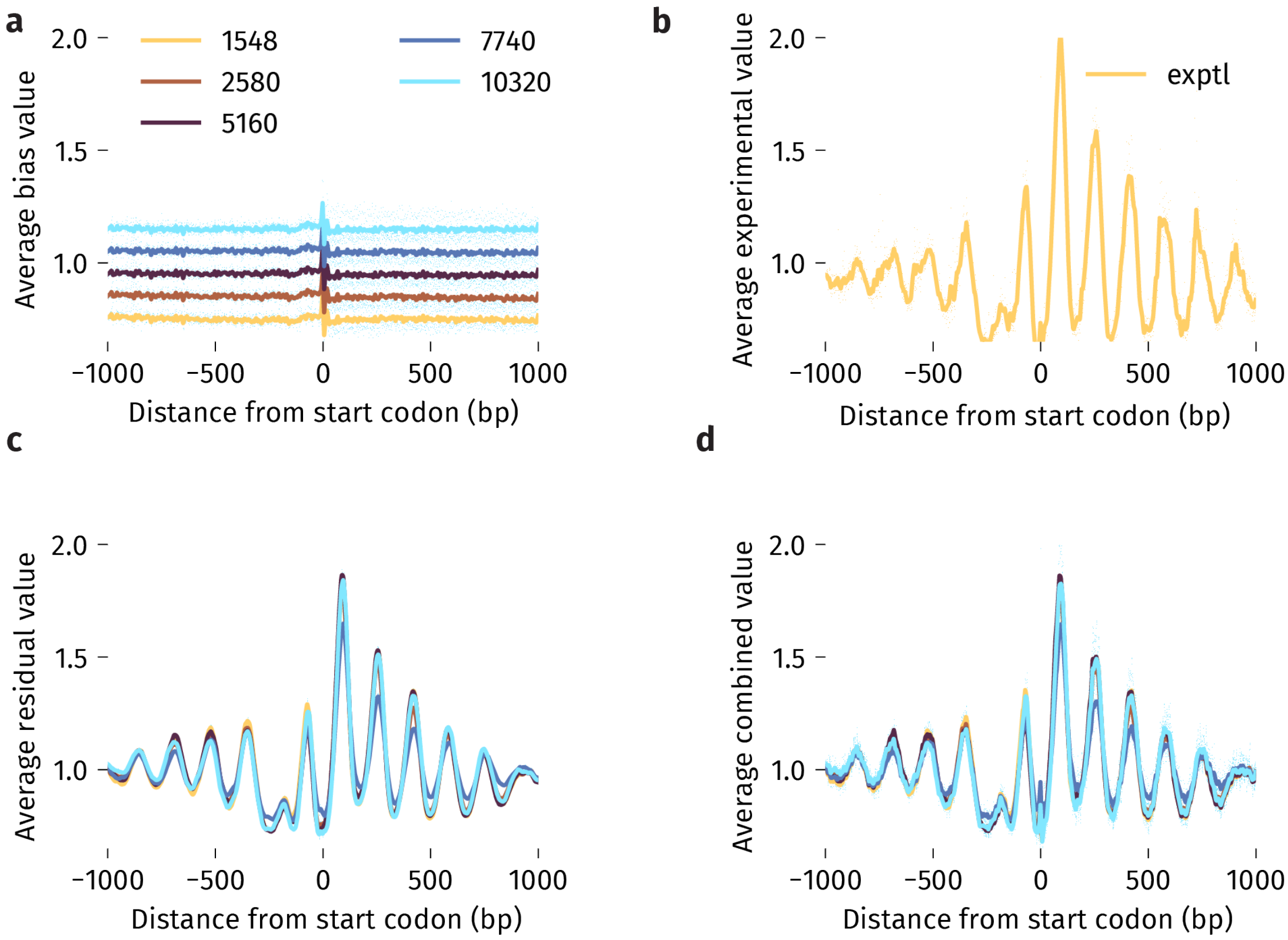

Supplementary Figure 7 Bias models can be trained with very little data. Since calculating PISA values genome-wide is not feasible in larger genomes, we trained bias models on subsets of our synthetic bias track to see how much data is necessary to train a high-quality bias model. To select the subsets, we sorted the regions by the variance of the fragment lengths in the experimental data and selected the regions with the most uniform fragment lengths. We trained models on 15, 25, 50, 75, and 100 percent of the synthetic bias track, corresponding to 1548, 2580, 5160, 7740, and 10320 training regions. In all cases, the bias correction was very similar, suggesting that the bias model requires very little data to train. To assess how much biological signal had leaked into the bias model, we calculated the average predicted profile around each gene start. (a) The average profile of the bias model at gene start sites. Tracks are offset for clarity of visualization. In all cases, the bias model shows a similar and largely featureless profile. The flat profile shows that the bias model has not learned nucleosome positioning around promoters. (b) The average experimental MNase-seq profile at the same regions, showing the classic nucleosome organization at promoters. (c) The average profile from our bias-corrected residual models. All of the predicted profiles are very similar, even when the bias model is trained on very little data. (d) The average profiles of our combined models. Again, all of the predicted profiles look very similar to the experimental profile in (b).

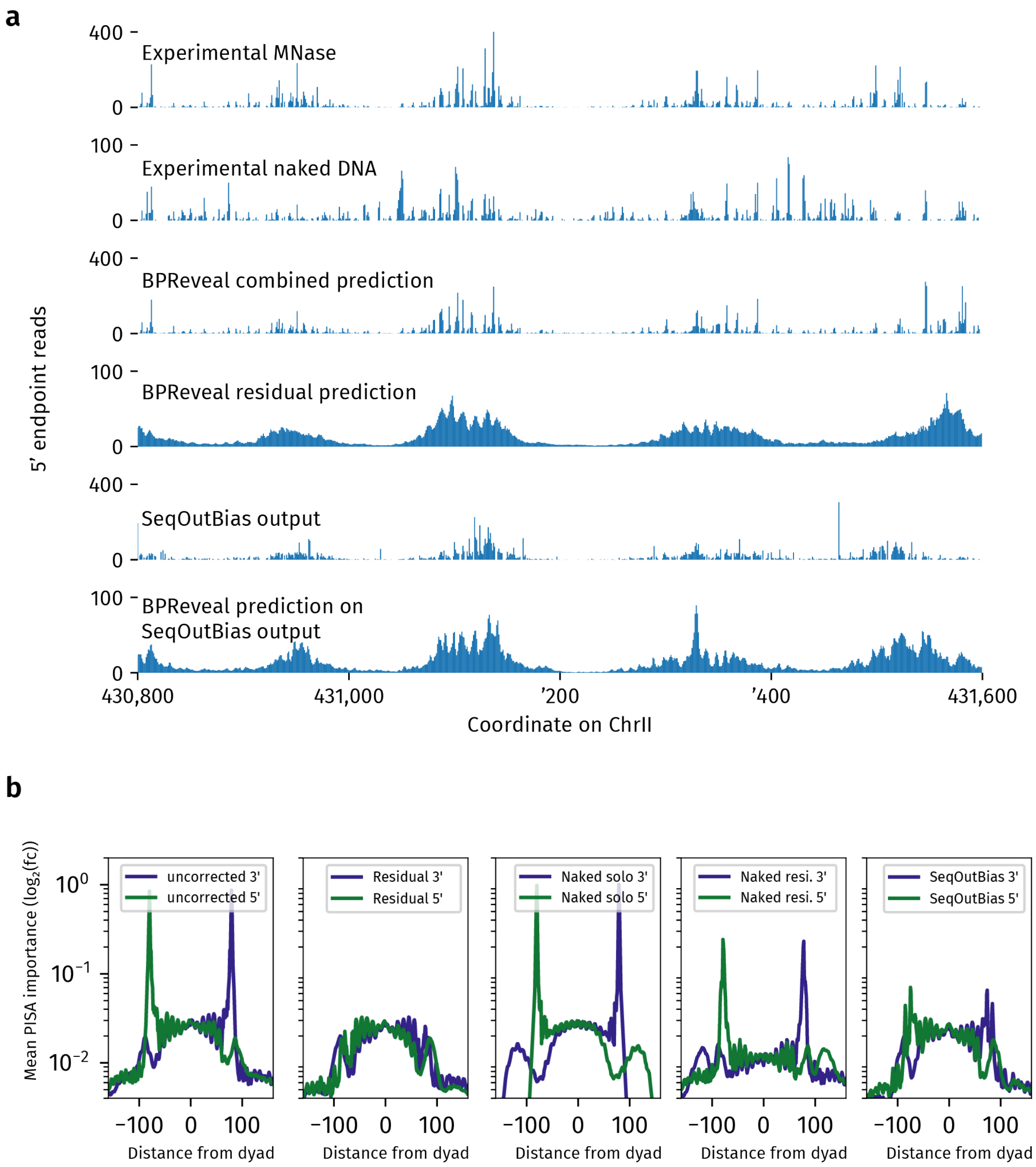

Supplementary Figure 8 Comparison of other bias-reduction techniques. In (a), we show several tracks that explore potential bias-correction strategies. In each track, we show only 5′ endpoints of the dataset. Experimental MNase is the MNase dataset from[3]. The experimental naked DNA sample is also from[3] and captures the behavior of the MNase enzyme on naked DNA. The BPReveal combined prediction is a BPReveal model trained on the experimental MNase data, including enzymatic bias. The BPReveal residual prediction is the bias-minimized result using the technique described in this paper. The SeqOutBias output is the track generated by SeqOutBias when given the experimental MNase data. We also trained a BPReveal model on the bias-minimized output from SeqOutBias, which is shown in the bottom track. In (b), we use BAT plots to quantify the effectiveness of bias removal using three techniques. The uncorrected model is trained on MNase data without any bias correction and shows a maximum value of 0.87, the residual model uses the bias removal strategy described in this paper and has a maximum value inside the nucleosome of 0.03, the naked solo model is trained only on the naked DNA experimental track but still shows contribution from inside the nucleosome body and a peak BAT value of 1.01, and the naked residual model is trained using the naked DNA model as a bias model and performing a ChromBPNet-style correction to remove that bias, which leaves a bias spike value of 0.23. Finally, the SeqOutBias model was trained on the bias-minimized output from SeqOutBias run on the experimental MNase data and shows a bias spike BAT value of 0.07.

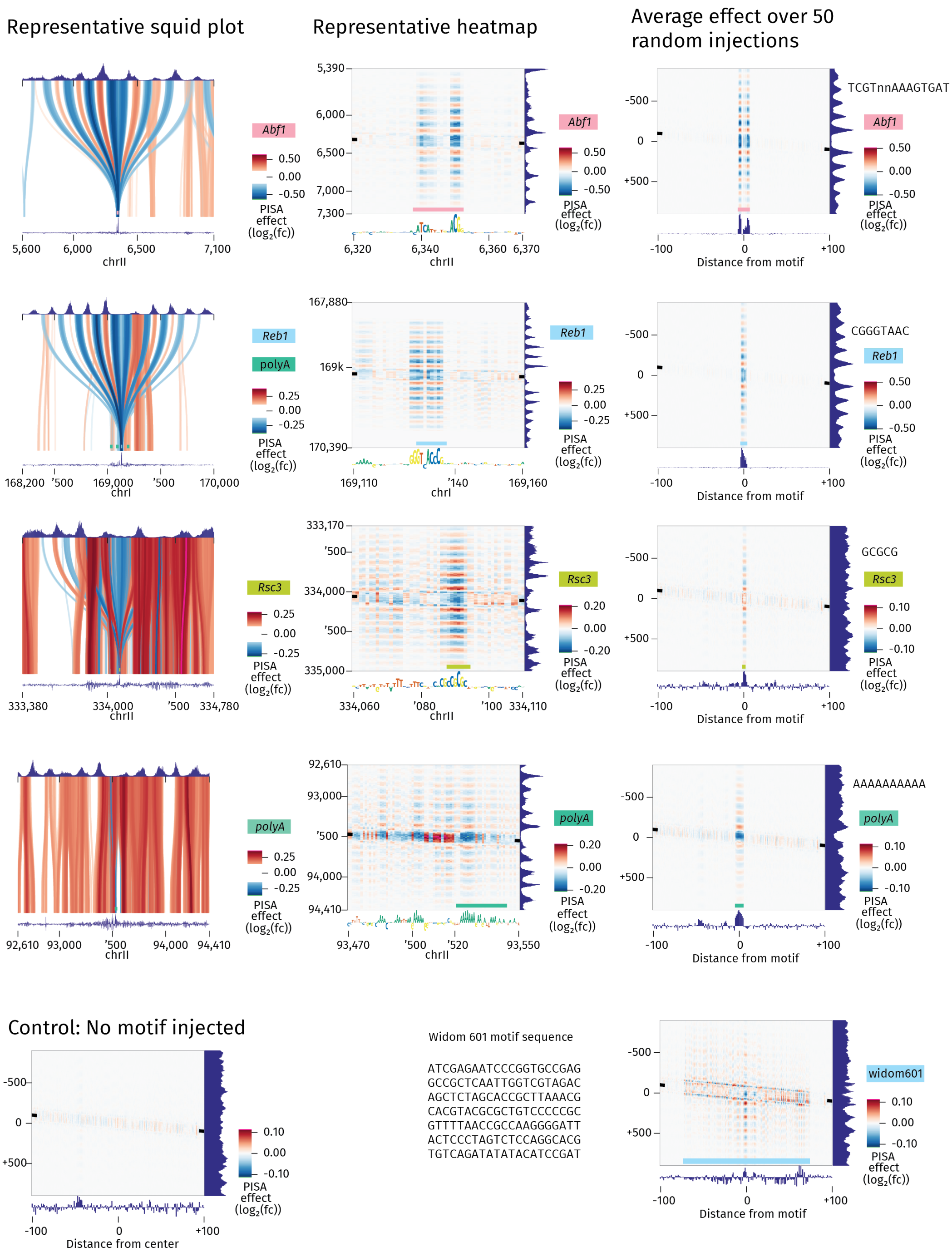

Supplementary Figure 9 All MNase PISA plots. For completeness, we show PISA squid plots (left) and heatmaps (center) for representative instances of the four motifs discussed in Figure 5 along with average PISA heatmaps generated by injecting the motif in 50 random genomic regions (right). The *Reb1* and *Abf1* motifs both show a characteristic strong positioning effect both in their actual genomic context and when injected into randomly-selected sequences from elsewhere in the genome. The *Rsc3* motif has a weaker effect than the two TFs, but still leads to nucleosome positioning. The *polyA* motif has a more distributed role. While one stretch of `A` has been identified by our motif mapping tool, several other short stretches of `A` repeats are also playing a role in nucleosome positioning at this locus. (bottom, left) We show the average PISA heatmaps drawn from 50 randomly-selected genomic regions. All of the injection effects in the right column are considerably stronger than the background level. (bottom, right) The effect of injecting the entire Widom 601 sequence is comparable to the effect of a single *polyA* or *Rsc3* motif injection, despite the Widom sequence being over an order of magnitude longer and specifically designed to position nucleosomes *in vitro*[5].

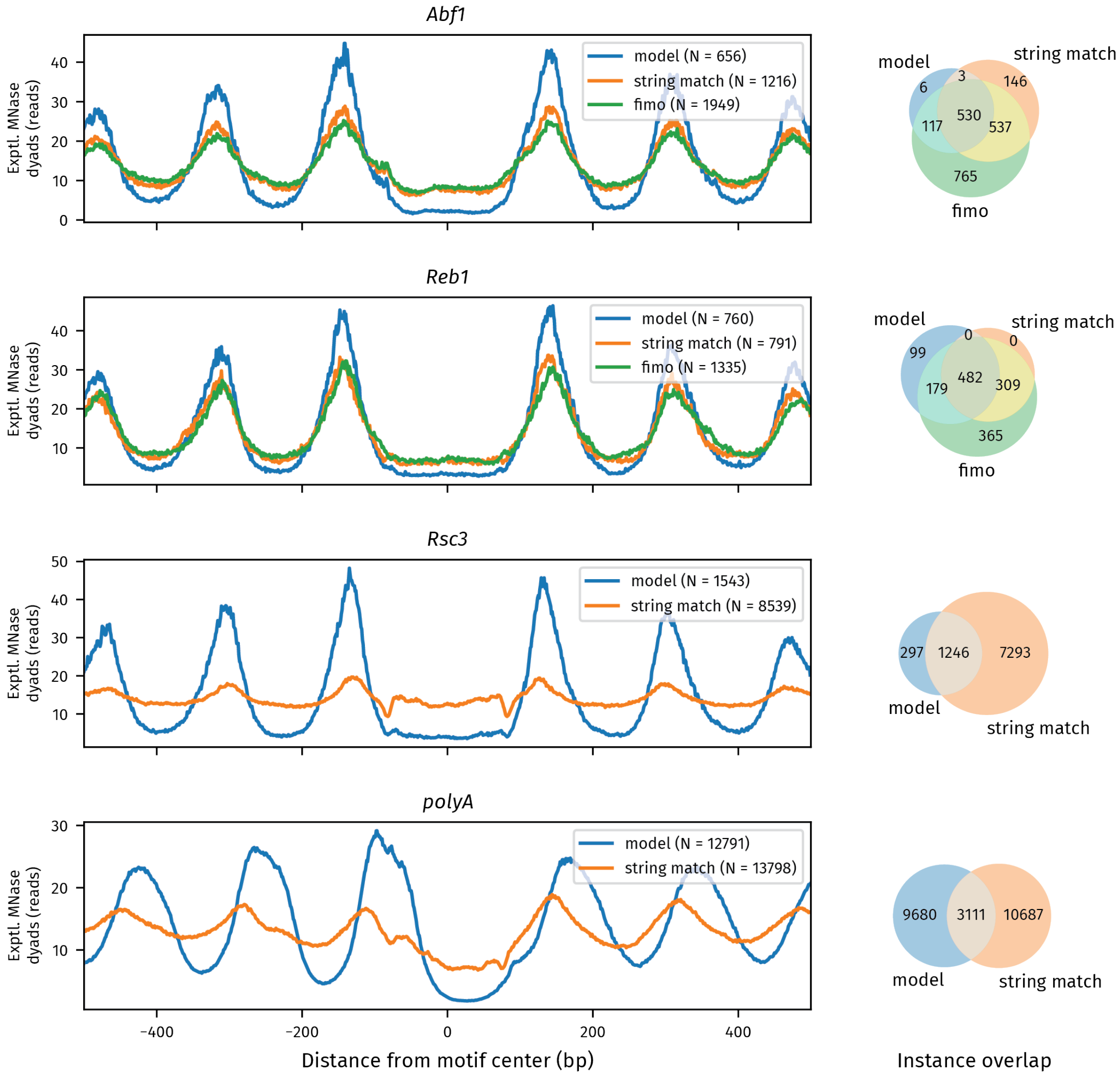

Supplementary Figure 10 Four motifs identified by MNase residual model For each motif, we show the average dyad density around that motif when called by three different methods. "model" motifs were discovered by scanning importance scores using the TF-MoDISco patterns. "fimo" motifs are identified using the motif as defined in JASPAR, and are identified purely by sequence match. "string match" motifs are based on scanning the genome to exact matches for a regular expression that matches the motif. The Venn diagram on the right shows how often the motif instances identified by the various methods overlap. For *Abf1* and *Reb1*, the model tends to identify motifs with stronger effects (blue lines) than the text-based algorithms (green and orange lines). For *Rsc3*, even though an *Rsc3* motif is in the JASPAR database, `fimo` did not identify any instances. The advantage of using a model to identify motifs is evident in that model-derived *Rsc3* motifs have much stronger nucleosome positioning effect than pure sequence-based motif calls. For *polyA*, there is no canonical JASPAR motif, and so `fimo` cannot be straightforwardly applied. Again, the model is much better at identifying functional *polyA* motifs than a simple sequence match.

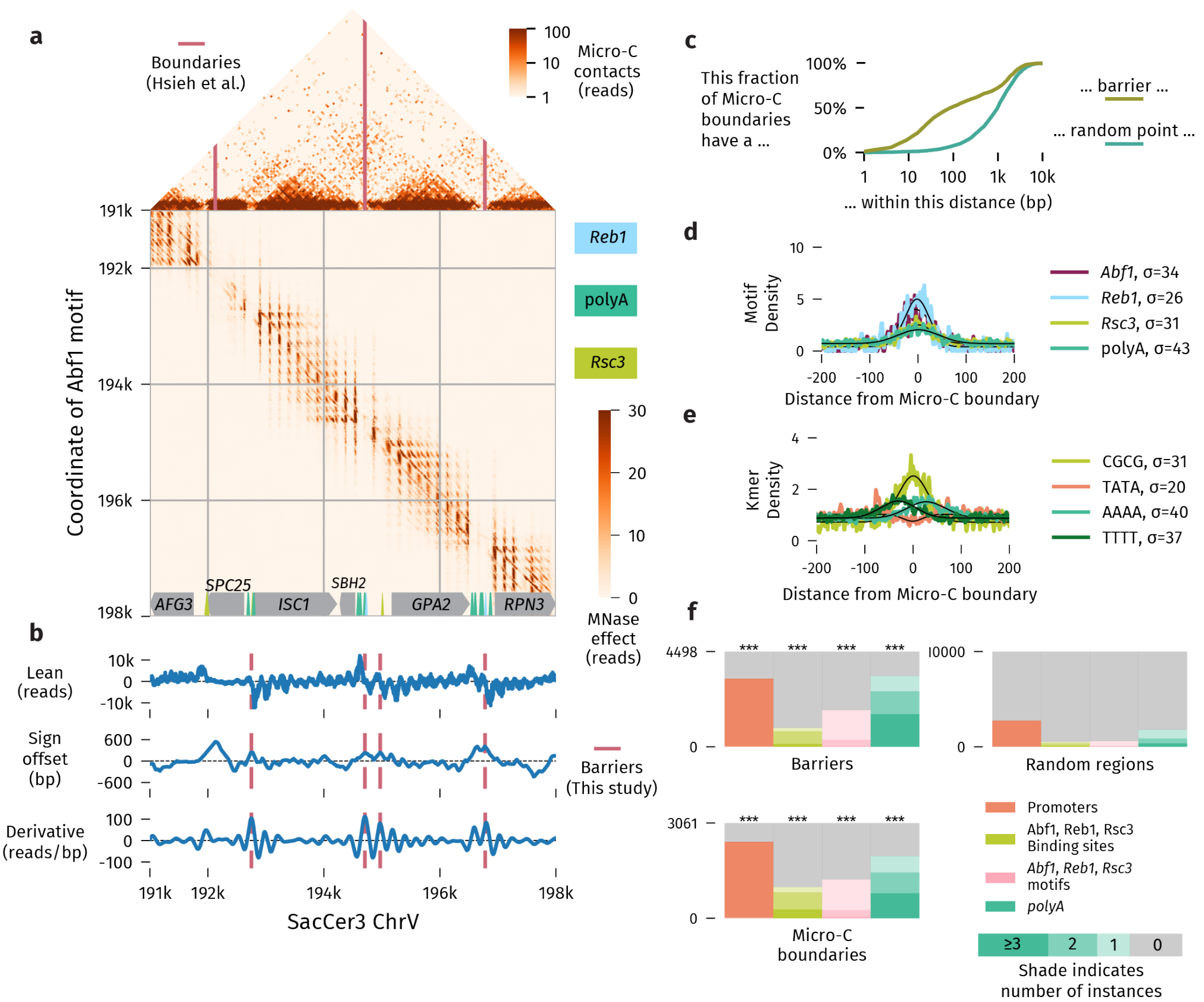

Supplementary Figure 11 Additional barrier-like elements. (a) Effects of inserting an *Abf1* motif at the same region used in Oberbeckmann et al.[6]. Our results are concordant with the results in Figure 2 of their work. With the improved resolution afforded by our motif-scanning strategy, we can observe a single nucleosome between the promoters of *SBH2* and *GPA2* that seems to be an island unto itself. Further, our model shows that the NDR between *SPC25* and *AFG3* is not caused by an *Abf1* motif as suggested previously[6], but rather by an *Rsc3* motif at the end of the *SPC25* open reading frame. (b) Lean scores and called peaks for barrier elements in the same region. Pink lines indicate consensus peaks. The sign offset and derivative scores used to call barrier peaks are described in the methods. It is interesting to note that the derivative-based analysis fails to detect a barrier in the very wide NDR on the left of the region, but clearly shows the lonely nucleosome between *SBH2* and *GPA2* (c) Our barriers overlap well with boundaries. For each Micro-C boundary, we identified the closest barrier annotated by our method. We show the cumulative fraction of boundaries that have a barrier within the given distance range (olive). We performed the same calculation using a random set of points (teal); random points are an order of magnitude further from Micro-C boundaries than our barriers. (d) Enrichment of four motifs at boundaries identified using Micro-C data[7]. The strong *Abf1* and *Reb1* motifs are highly enriched within a small window around the domain boundaries, while the *polyA* motif is more broad. (e) Enrichment of four kmers around boundaries identified previously[7]. The pattern is similar to that observed with our method, though our perturbation-based analysis is able to map these sequence elements at higher resolution. (f) Overlap of our barriers and published Micro-C boundaries with several region sets. For each barrier and boundary, we searched 147 bp upstream and downstream and counted the number of Abf1, Reb1, and Rsc3 binding sites determined by ChIP-exo (yellow)[8], instances of the corresponding motifs identified by our model (pink), model-identified *polyA* motifs (green), and promoters (orange). The bars are colored according to the number of times a particular type of region occurred in a barrier region. The lightest color represents one overlap, the middle color represents two, and the darkest color represents three or more. For example, our barriers often overlap with three or more *polyA* motifs since the dark green bar is larger than either of the other two green bars. Conversely, most barriers overlap with exactly one promoter since the light orange bar is far larger than the darker orange segments. For comparison, we also calculated the same overlap distributions for random regions of the genome. For each overlap category, we performed a chi-squared test to determine if the overlap counts in the barriers differed from the count from a random sample of the genome. In every case, the barriers and boundaries are statistically significantly enriched relative to random regions ($p < 10^{-10}$, Bonferroni corrected). While barriers and boundaries are certainly enriched for *Abf1*, *Rsc3*, and *Reb1*, it is striking that over half of the barriers and boundaries do not co-occur with one of those motifs. In many cases, barrier formation appears to be mediated by multiple *polyA* motifs.

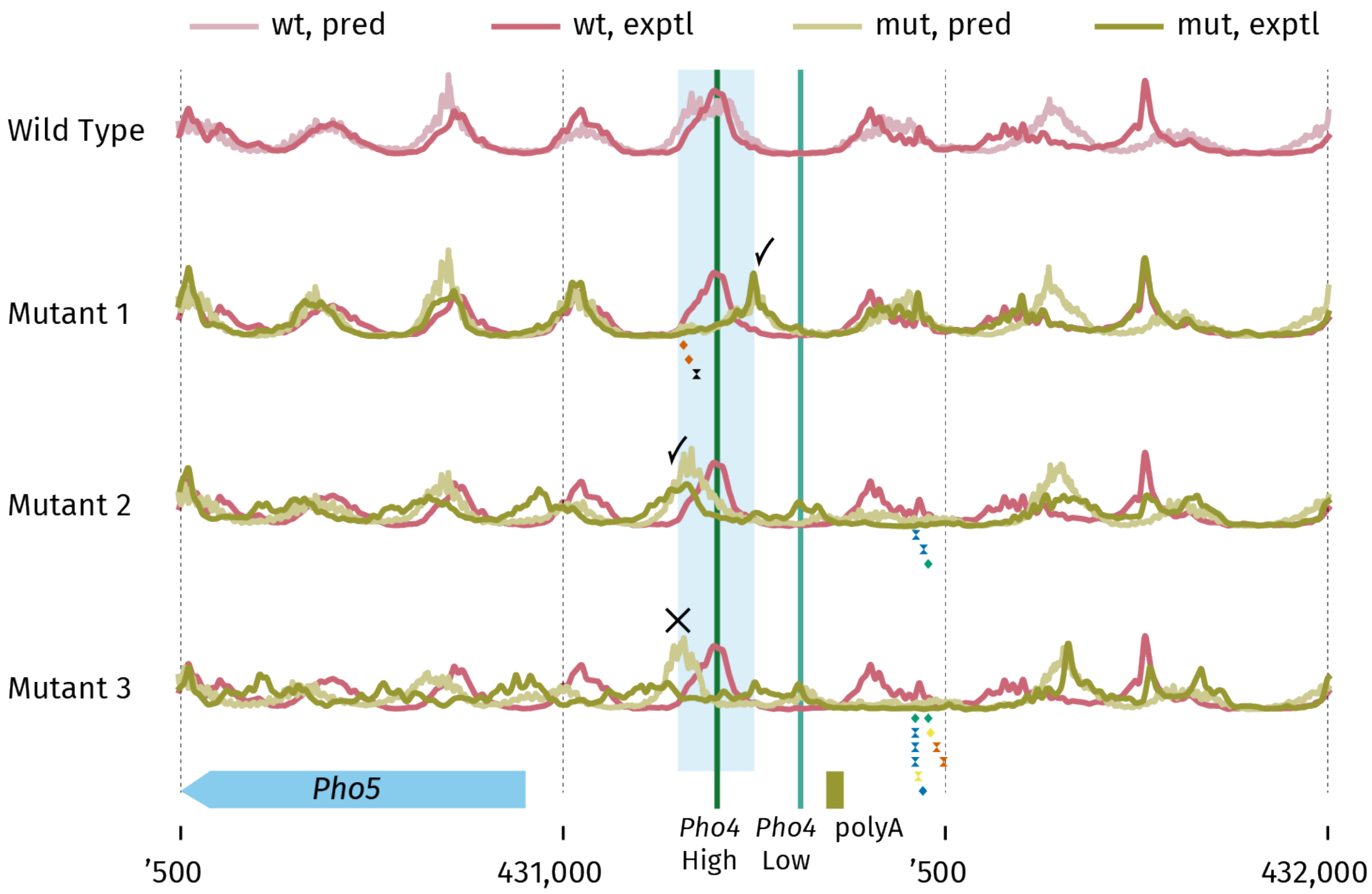

Supplementary Figure 12 Additional CRISPR experiments. We constructed three CRISPR lines using the genetic algorithm. Mutant 1 is the one presented in the main text. Its fitness function aimed to reduce nucleosome density in the blue region. The other two mutants (2 and 3) were designed using a fitness function that increased nucleosome density over both *Pho4* motifs. Mutant 3 was allowed to introduce 10 mutations, whereas 2 was allowed 5. Curiously, in both cases, the GA settled on a design where the mutations are introduced several hundred bases away from the *Pho4* motifs. Experimental MNase-seq data generated using CRISPR-edited yeast strains showed that mutant 1 and 2 produced the desired effect, whereas the model mis-predicted a nucleosome to the left of the high-affinity *Pho4* motif in mutant 3. Unfortunately, neither the prediction nor the experimental MNase data for mutants 2 and 3 show a strong nucleosome over the low-affinity *Pho4* site, suggesting that such a chromatin configuration is not readily achieved.

Supplementary Table 5 Reproducibility of CRISPR strain MNase-seq. We compare our CRISPR replicate datasets and mutant lines using the same Pearson's R method used to calculate performance metrics. The profile score is the average correlation between the individual reads in corresponding 1000 bp windows, while the counts score measures the total number of reads in each of the 1000 bp windows and then measures the correlation between those sums between two different datasets.

Dataset 1	Dataset 2	Profile correlation	Counts correlation
WT rep 1	WT rep 2	0.922	0.860
1A rep 1	1A rep 2	0.899	0.766
1B rep 1	1B rep 2	0.950	0.978
2A rep 1	2A rep 2	0.940	0.925
1A pooled	WT pooled	0.951	0.956
1B pooled	WT pooled	0.970	0.963
1B pooled	1A pooled	0.948	0.915
2A pooled	WT pooled	0.964	0.956
2A pooled	1A pooled	0.962	0.970
2A pooled	1B pooled	0.959	0.902

Supplementary Table 6 Timing data for BPReveal tasks BPReveal models are generally small enough that they can be trained and interpreted on commodity hardware. The data on this table are collected on a computer with 64 GB of RAM, 24 CPU cores, and a single NVIDIA GeForce RTX 3090 GPU. Collecting genome-wide PISA to construct the synthetic bias track took several days on three A100 GPUs.

Task	Job size	Time (h:mm:ss)	Time per item
Building ATAC training data	46848 regions	0:00:16	-
Building OSKN training data	142746 regions, 8 tasks	0:02:51	-
Training ATAC bias model	82 Epochs	0:06:50	4.6 s / epoch
Training ATAC combined model	27 Epochs	0:09:36	20 s/epoch
Training MNase transformation model	75 epochs	0:13:42	10 s/epoch
Training MNase combined model	71 epochs	0:38:43	32 s/epoch
Predicting MNase genome-wide	23864 regions	0:00:23	1549 regions/s
Predicting OSKN binding on peaks	116085 regions, 8 tasks	0:01:57	1062 regions/s
Calculating ATAC contribution scores	36981 regions	0:23:17	28 regions/s
Calculating MNase contribution scores	23864 regions, 2 tasks	0:33:32	12.5 regions/s
PISA on one region, OSKN model	2400 bp	0:01:17	33 bp/s
PISA to calculate synthetic bias	100000 bp	1:04:23	26 bp/s
ATAC motif scanning	36981 regions, 7 motifs	0:01:20	500 regions/s

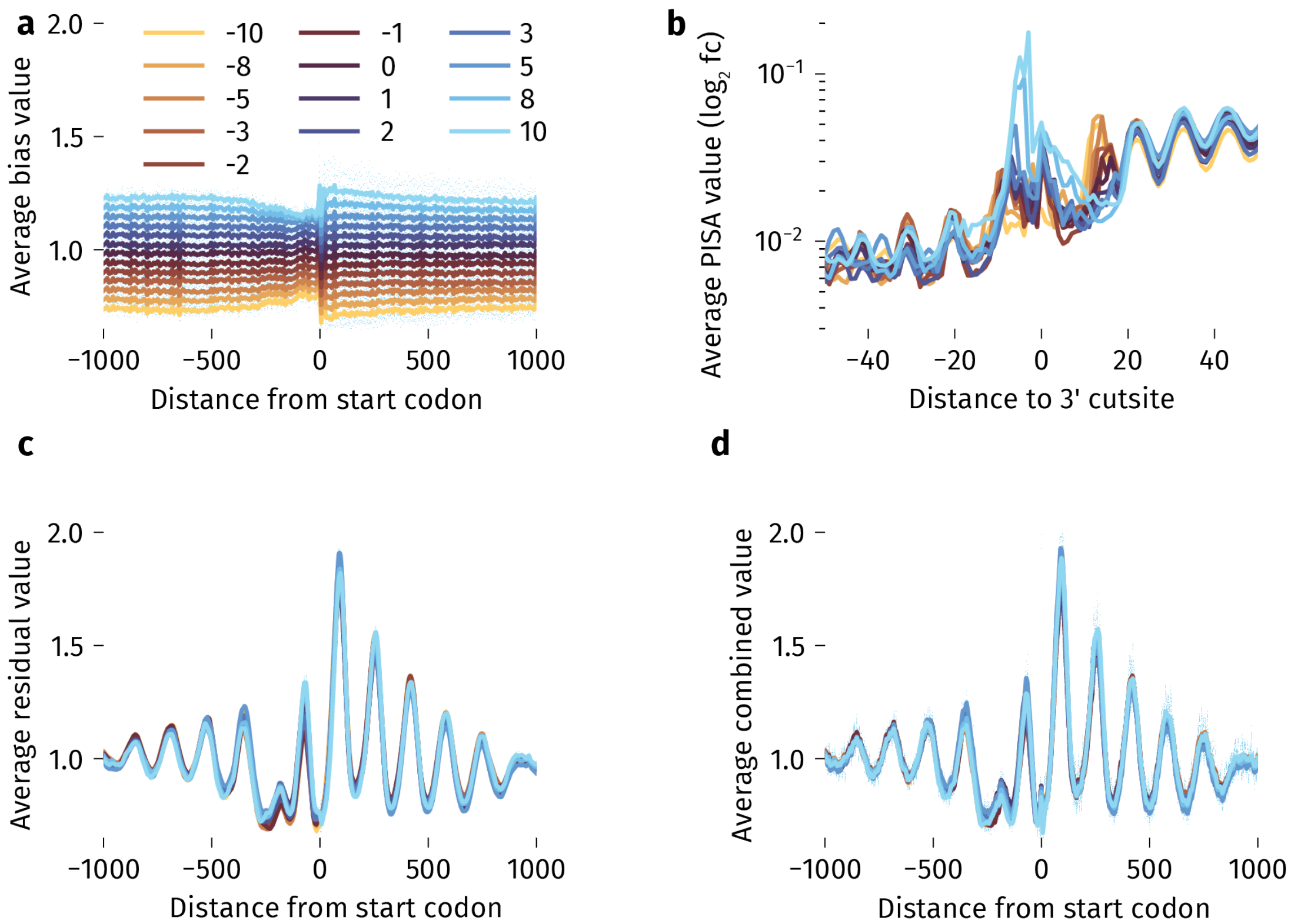

Supplementary Figure 13 The offset used to construct the synthetic bias track is flexible. The offset of 179 bp used to calculate our synthetic bias track is very close to the average length of the fragments in our MNase-seq dataset. To determine how precise this offset must be, we trained models using offset values from 169 (yellow) to 189 (blue). We show metapeaks over every gene start, as in Supplementary Figure 7. (a) The average profile of the bias models is relatively uniform, though offsets above 5 bp show a symmetric pattern around the gene start. This shows that offset values can be off by up to 5 bp without introducing a non-bias signal into the bias model. The traces in this plot are offset in the y-axis for visual clarity. (b) BAT plots of residual models, showing that the spike at the cutsite is well-corrected except for large positive offsets. (c) The average profile from our bias-corrected residual models. On the whole, the profiles are very similar for every offset value. (d) The average profiles of our combined models. Again, all models agree well with experimental profiles.

1 Supplementary References

References

[1] Avsec, v. *et al.* Base-resolution models of transcription-factor binding reveal soft motif syntax. *Nature Genetics* **53**, 354–366 (2021). URL http://dx.doi.org/10.1038/s41588-021-00782-6.

[2] Brennan, K. J. *et al.* Chromatin accessibility in the *Drosophila* embryo is determined by transcription factor pioneering and enhancer activation. *Developmental Cell* **58**, 1898–1916.e9 (2023). URL https://linkinghub.elsevier.com/retrieve/pii/S1534580723003477.

[3] Begley, V. *et al.* Xrn1 influence on gene transcription results from the combination of general effects on elongating RNA pol II and gene-specific chromatin configuration. *RNA Biology* **18**, 1310–1323 (2021). URL http://dx.doi.org/10.1080/15476286.2020.1845504.

[4] Routhier, E., Pierre, E., Khodabandelou, G. & Mozziconacci, J. Genome-wide prediction of DNA mutation effect on nucleosome positions for yeast synthetic genomics. *Genome Research* **31**, 317–326 (2021). URL http://dx.doi.org/10.1101/gr.264416.120.

[5] Thåström, A. *et al.* Sequence motifs and free energies of selected natural and non-natural nucleosome positioning DNA sequences. *Journal of Molecular Biology* **288**, 213–229 (1999). URL http://dx.doi.org/10.1006/jmbi.1999.2686.

[6] Oberbeckmann, E., Quililan, K., Cramer, P. & Oudelaar, A. M. In vitro reconstitution of chromatin domains shows a role for nucleosome positioning in 3D genome organization. *Nature Genetics* **56**, 483–492 (2024). URL http://dx.doi.org/10.1038/s41588-023-01649-8.

[7] Hsieh, T.-H. S. *et al.* Mapping nucleosome resolution chromosome folding in yeast by micro-c. *Cell* **162**, 108–119 (2015). URL http://dx.doi.org/10.1016/j.cell.2015.05.048.

[8] Rossi, M. J. *et al.* A high-resolution protein architecture of the budding yeast genome. *Nature* **592**, 309–314 (2021). URL http://www.nature.com/articles/s41586-021-03314-8.
