# Supplementary material for: Positional interpretation of cis-regulatory code and nucleosome organization with deep learning models
Source: Nat Commun. 2026 Jul 4;17:8326. doi: 10.1038/s41467-026-74807-1 (PMC13469612; doi:10.1038/s41467-026-74807-1)
Supplement: Supplementary file 2 [file 41467_2026_74807_MOESM2_ESM.pdf]

Description of Additional Supplementary Files

Supplementary Data 1: DNA sequences for gRNA and HDR templates used to construct mutant yeast strains.

Supplementary Video 1: We generated PISA squid plots for every Reb1 motif instance in our validation and test sets, and sorted them by how the motif effect leans. It is striking that in many instances where the motif's effect is asymmetric, there is not another nearby motif that seems to conflict with it.
