# Supplementary material for: Positional interpretation of cis-regulatory code and nucleosome organization with deep learning models
Source: Nat Commun. 2026 Jul 4;17:8326. doi: 10.1038/s41467-026-74807-1 (PMC13469612; doi:10.1038/s41467-026-74807-1)
Supplement: Supplementary file 3 [file 41467_2026_74807_MOESM5_ESM.pdf]

nature portfolio

Corresponding author(s): Julia Zeitlinger

Last updated by author(s): Jun 4, 2026

Reporting Summary

Nature Portfolio wishes to improve the reproducibility of the work that we publish. This form provides structure for consistency and transparency in reporting. For further information on Nature Portfolio policies, see our Editorial Policies and the Editorial Policy Checklist.

Statistics

For all statistical analyses, confirm that the following items are present in the figure legend, table legend, main text, or Methods section.

n/a	Confirmed	
☐	☒	The exact sample size (n) for each experimental group/condition, given as a discrete number and unit of measurement
☒	☐	A statement on whether measurements were taken from distinct samples or whether the same sample was measured repeatedly
☒	☐	The statistical test(s) used AND whether they are one- or two-sided<br>*Only common tests should be described solely by name; describe more complex techniques in the Methods section.*
☒	☐	A description of all covariates tested
☒	☐	A description of any assumptions or corrections, such as tests of normality and adjustment for multiple comparisons
☐	☒	A full description of the statistical parameters including central tendency (e.g. means) or other basic estimates (e.g. regression coefficient) AND variation (e.g. standard deviation) or associated estimates of uncertainty (e.g. confidence intervals)
☒	☐	For null hypothesis testing, the test statistic (e.g. F, t, r) with confidence intervals, effect sizes, degrees of freedom and P value noted<br>*Give P values as exact values whenever suitable.*
☒	☐	For Bayesian analysis, information on the choice of priors and Markov chain Monte Carlo settings
☒	☐	For hierarchical and complex designs, identification of the appropriate level for tests and full reporting of outcomes
☐	☒	Estimates of effect sizes (e.g. Cohen's d, Pearson's r), indicating how they were calculated

Our web collection on statistics for biologists contains articles on many of the points above.

Software and code

Policy information about availability of computer code

Data collection	sratoolkit 3.2.0
Data analysis	bedtools 2.31.1, cpython 3.12.11, gcc 15.1.0, h5py 3.14.0, Keras 3.11.3, modisco 2.4.0, numpy 2.0.1, pybedtools 0.12.0, pybigwig 0.3.24, pysam 0.23.3, scipy 1.16.2, tensorflow 2.20.0, tf-keras 2.20.1, bpreveal 5.2.2, bowtie2 2.5.1, meme 5.5.8, samtools 1.20, sratoolkit 3.2.0, Code for the paper is available at https://github.com/zeitlingerlab/bpreveal-manuscript.

For manuscripts utilizing custom algorithms or software that are central to the research but not yet described in published literature, software must be made available to editors and reviewers. We strongly encourage code deposition in a community repository (e.g. GitHub). See the Nature Portfolio guidelines for submitting code & software for further information.

Data

Policy information about availability of data

All manuscripts must include a data availability statement. This statement should provide the following information, where applicable:

- Accession codes, unique identifiers, or web links for publicly available datasets
- A description of any restrictions on data availability
- For clinical datasets or third party data, please ensure that the statement adheres to our policy

All analysis code, predicted tracks, models, configuration files, importance score tracks, and motifs have been deposited in Zenodo under accession code 20318019 [zenodo.org/records/20318019] and the Stowers Original Data Repository[https://www.stowers.org/research/publications/libpb-2546].
Additionally, all training data, raw model predictions, raw PISA values, and raw importance scores used in this study have been deposited in the Stowers Original

Data Repository[https://www.stowers.org/research/publications/libpb-2546]. The sequencing data for the Pho5 site mutations generated in this study have been deposited in the NCBI SRA under accession GSE313524[www.ncbi.nlm.nih.gov/geo/query/acc.cgi?acc=GSE313524]. The data used to train the OSKN model are available on the NCBI SRA under accession GSE137193[www.ncbi.nlm.nih.gov/geo/query/acc.cgi?acc=GSE137193]. The data used to train the accessibility model and H3K27Ac model are available on the NCBI SRA under accession GSE218852[www.ncbi.nlm.nih.gov/geo/query/acc.cgi?acc=GSE218852]. The data used to train MNase model are available on the NCBI SRA under accession GSE153035[www.ncbi.nlm.nih.gov/geo/query/acc.cgi?acc=GSE153035]. The Micro-C data used to illustrate boundaries are available on the NCBI SRA under accession GSE68016[www.ncbi.nlm.nih.gov/geo/query/acc.cgi?acc=GSE68016].

Research involving human participants, their data, or biological material

Policy information about studies with human participants or human data. See also policy information about sex, gender (identity/presentation), and sexual orientation and race, ethnicity and racism.

Reporting on sex and gender	This research did not involve human participants.
Reporting on race, ethnicity, or other socially relevant groupings	This research did not involve human participants.
Population characteristics	This research did not involve human participants.
Recruitment	This research did not involve human participants.
Ethics oversight	This research did not involve human participants.

Note that full information on the approval of the study protocol must also be provided in the manuscript.

Field-specific reporting

Please select the one below that is the best fit for your research. If you are not sure, read the appropriate sections before making your selection.

[x] Life sciences [ ] Behavioural & social sciences [ ] Ecological, evolutionary & environmental sciences

For a reference copy of the document with all sections, see nature.com/documents/nr-reporting-summary-flat.pdf

Life sciences study design

All studies must disclose on these points even when the disclosure is negative.

Sample size	Two replicate MNase-seq experiments were performed on the CRISPR-derived Pho5 mutant lines, in line with typical practices in genomics.
Data exclusions	We excluded one of our four mutants since we discovered that the CRISPR had failed to introduce the correct mutation.
Replication	We performed replicate MNase-seq experiments on all of our mutants; the results were reproducible.
Randomization	Randomization is not applicable for this study design.
Blinding	Blinding was not necessary for this study.

Reporting for specific materials, systems and methods

We require information from authors about some types of materials, experimental systems and methods used in many studies. Here, indicate whether each material, system or method listed is relevant to your study. If you are not sure if a list item applies to your research, read the appropriate section before selecting a response.

Materials & experimental systems

n/a	Involved in the study	
[x]	[ ]	Antibodies
[x]	[ ]	Eukaryotic cell lines
[x]	[ ]	Palaeontology and archaeology
[ ]	[x]	Animals and other organisms
[x]	[ ]	Clinical data
[x]	[ ]	Dual use research of concern
[x]	[ ]	Plants

Methods

n/a	Involved in the study	
[x]	[ ]	ChIP-seq
[x]	[ ]	Flow cytometry
[x]	[ ]	MRI-based neuroimaging

Animals and other research organisms

Policy information about studies involving animals; ARRIVE guidelines recommended for reporting animal research, and Sex and Gender in Research

Laboratory animals	This study did not involve laboratory animals.
Wild animals	This study did not involve wild animals.
Reporting on sex	Sex information was not collected in this study.
Field-collected samples	This study did not involve field-collected samples.
Ethics oversight	No ethics approval was required for research on S. cerevisiae.

Note that full information on the approval of the study protocol must also be provided in the manuscript.

Plants

Seed stocks	This research did not involve plants.
Novel plant genotypes	This research did not involve plants.
Authentication	This research did not involve plants.
