# Supplementary material for: Positional interpretation of cis-regulatory code and nucleosome organization with deep learning models
Source: Nat Commun. 2026 Jul 4;17:8326. doi: 10.1038/s41467-026-74807-1 (PMC13469612; doi:10.1038/s41467-026-74807-1)
Supplement: Supplementary file 4 [file 41467_2026_74807_MOESM6_ESM.pdf]

nature portfolio

Peer Review File

Positional Interpretation of Cis-Regulatory Code and Nucleosome Organization with Deep Learning Models

Corresponding Author: Professor Julia Zeitlinger

Parts of this Peer Review File have been redacted as indicated to maintain the confidentiality of unpublished data.

This file contains all reviewer reports in order by version, followed by all author rebuttals in order by version.

Version 0:

Reviewer comments:

Reviewer #1

(Remarks to the Author)
Summary
McAnany et al. introduce PISA (Pairwise Influence by Sequence Attribution), an interpretability framework for sequence-to-profile neural networks. By avoiding the aggregation of contribution scores across the output window, PISA aims to resolve the specific spatial range of motif effects. The authors apply this tool to ChIP-nexus/seq, ATAC-seq, and MNase-seq data, introducing a novel method for synthesizing enzymatic bias tracks for MNase-seq by exploiting 5'/3' nucleosome symmetry. Finally, they use the model to define chromatin barrier elements and experimentally validate a genetic algorithm for nucleosome engineering at a single yeast locus.
The spatial visualizations and MNase bias-correction logic are innovative, and the authors demonstrate them in intriguing and exciting applications. The manuscript often leans on visual intuition over unassailable quantitative rigor. For the most part, it toes my comfort line; I don't leave the paper overly suspicious of any claims, but I do believe the claims could be strengthened in several places that I comment on below.

Major Comments
MNase bias scalability. The authors note that calculating PISA genome-wide for S. cerevisiae took "a few days on three Nvidia A100 GPUs" and suggest that for larger genomes, one should train a bias model on a subset of PISA-derived data. To substantiate this recommendation, it would be valuable to see a learning curve analysis: training the bias model on increasingly large subsets of the yeast genome and evaluating its prediction accuracy against the full genome-wide PISA calculation. This would empirically define how much synthetic training data is sufficient and demonstrate the feasibility of this strategy for mammalian genomes.

Framing of PISA Novelty. The manuscript frames PISA as a "new interpretation tool" that "overcomes the obstacles" of current attribution methods (Introduction). However, mathematically, PISA is the application of DeepSHAP to individual output positions without the subsequent aggregation step typically performed in the field. The PISA value P_ij is simply the raw Shapley value matrix, which DeepSHAP inherently calculates. While visualizing this matrix (via squid plots and heatmaps) is a valuable contribution and yields new biological insights, framing it as a novel algorithm or tool distinct from DeepSHAP is misleading. I recommend revising the text to characterize PISA as a visualization framework or analytical strategy that leverages existing attribution scores, rather than a new attribution method itself.

Chromatin barrier quantification. The authors claim their model identifies barrier elements that "resemble Micro-C interaction maps". Currently, this claim is supported primarily by visual comparison of the RER2 locus. The analysis would be strengthened by a genome-wide statistical comparison between their predicted "lean" values/barriers and experimental Micro-C boundary scores.

Barrier conceptual circularity. In Figure 6, the authors define barrier elements using a "lean" metric (resistance to nucleosome shifting) and report these are enriched for Abf1 and Reb1 motifs. This reasoning appears circular. Abf1 and Reb1 are known to create rigid, phased nucleosomes; a model trained on MNase-seq must learn this. Therefore, "discovering" that barriers are Abf1/Reb1 sites effectively reduces to the trivial observation that strong nucleosome positioners resist shifting.

To demonstrate that barriers capture novel biology rather than just high-affinity binding, the authors should computationally decouple lean from standard occupancy. A scatter plot comparing Lean Score vs. Predicted Occupancy would clarify if the metric identifies a distinct mechanical property or merely proxies for GRF binding strength. Discordant sites (High Lean/Low Occupancy) would be particularly strong evidence of novelty.

Minor Comments
Robustness to biological asymmetry. The synthetic bias derivation assumes nucleosome signals are perfectly symmetric and that a single genome-wide offset (179bp) correctly aligns 5'/3' contributions at all loci. However, local variation in fragment length (e.g. due to partial unwrapping, remodeling, or sequence-dependent MNase digestion kinetics) would cause incomplete cancellation, potentially leaking biological signal into the bias track. While Extended Data Figure 4 suggests the bias model avoids capturing obvious regulatory motifs, this does not rule out the loss of subtler asymmetric features. To rigorously quantify this potential signal loss, I recommend the authors either (1) simulate asymmetric nucleosomes to establish a leakage error margin, or (2) demonstrate that the predicted "bias" magnitude does not systematically correlate with known asymmetric genomic features (e.g., +1 nucleosomes at TSSs). That being said, if the authors determine that these computational validations are out of scope, I would be satisfied with a revision to the Discussion that explicitly acknowledges this limitation.

Offset sensitivity. The synthetic bias derivation relies on a 179bp offset determined by maximizing agreement between shifted 5'/3' PISA matrices. How sensitive is the resulting bias track to this parameter? A brief sensitivity analysis showing that results are stable across a reasonable range (e.g., ±10bp) would increase confidence in the approach's robustness.

Experimental validation. The use of a Genetic Algorithm to engineer nucleosome positioning is a compelling proof-of-concept. However, validation is restricted to a single successful edit at the PHO5 promoter. While the Introduction frames this as a "proof-of-principle," the Results suggest broader capability. Could the authors clarify if other designs were attempted experimentally? Understanding the hit rate would be insightful. If further validation is not feasible, I recommend adding Discussion text explicitly framing this as an N=1 demonstration and outlining potential failure modes for broader application.

BPReveal bias transformation. In Methods Section 4.3, the authors introduce a more expressive, non-linear transformation for the bias model. Extended Data Table 2 indicates that this technique improves performance on both profile prediction (lower JSD) and total read count prediction (higher Spearman correlation) compared to the standard linear approach. Given this consistent improvement, do the authors recommend that this non-linear transformation become the new standard for all future BPNet/ChromBPNet applications, or are there specific contexts where the simpler linear transformation is still preferred?

tangermeme. Line 123 says tangermeme is “a PyTorch-based version of BPNet”, but its introducing manuscript describes it much differently.

(Remarks on code availability)
The results appear reproducible.

Reviewer #2

(Remarks to the Author)
This manuscript presents an innovative approach to attributions for sequence-to-function neural networks. PISA addresses several limitations of existing attribution methods by quantifying the impact of input sequence positions at each position in the output. This approach has several advantages, notably the ability to identify both positive and negative effects of individual motifs on model predictions. The higher-resolution attributions of PISA also facilitate identifying biases in experimental assays such as MNase-seq. The authors demonstrate that PISA can generate useful interpretations of neural networks, eliminate biases from MNase-seq for improved interpretability, and guide the design of experiments to change nucleosome positioning in vivo. The ability to attribute positive and negative effects to motifs proves especially useful for examining the impact of motifs on nucleosome positioning. The software comes as a useful general-purpose package with high quality visualization tools and will thus be useful to the field.

Comments:
1. PISA’s ability to deconvolve attributions between individual input and output positions is powerful and the manuscript compellingly demonstrates several important applications of the approach. But the validation of the individual motif effects revealed by the attributions is limited. Most of the vignettes show the results of PISA attributions on a single sequence in one condition. Experimental validation of attributions at scale is not feasible, but it would increase confidence in the method if the performance of the attributions were tested more extensively on some examples of motif or transcription factor perturbations, taken from published data. For example, there are some Zelda-bound loci that remain accessible in embryos lacking Zelda (PMID 26335634). Can PISA distinguish the effects of the Zelda motif at sites where Zelda is required for accessibility, from Zelda motifs that remain accessible in the absence of Zelda? One study examined NANOG binding when OCT4 was knocked down (PMID 34143975). Can sites bound by NANOG in the absence of OCT4 be identified by PISA (as sites where a nearby OCT4/SOX2 motif has no attribution connection to the NANOG motif)? Such comparisons wouldn’t necessarily require training new models. Published data could be used to identify Zelda-dependent vs Zelda-independent accessible sites, which could then be compared in the already-trained model.

2. The initial discussion of bias correction on p. 9 is difficult to follow. The second paragraph mentions BPReveal models trained to predict Drosophila embryo ATAC-seq data. I'm assuming these models were trained using the ChromBPNet strategy, but this isn't stated explicitly. The paragraph mentions a 'combined model'. Is this just a model without the bias removed? Is the main point of this section (discussing Fig. 4a,b) to demonstrate that PISA can visualize bias effects?

3. How reproducible are the new MNase-seq experiments? A measure of reproducibility should be included in the Methods.

(Remarks on code availability)
The code is well organized, well documented, and is broadly applicable. It will be a useful resource for the community.

Reviewer #3

(Remarks to the Author)
This manuscript introduces PISA, an interpretation method for sequence-to-profile models that assigns nucleotide contribution scores to each position of the predicted output profile. By retaining this two-dimensional attribution structure, PISA enables spatial analysis of nucleotide influence along the predicted output signal, providing higher resolution than standard attribution approaches based on profile summary statistics. The method is implemented within the BPReveal framework and applied across transcription factor binding, chromatin accessibility, and MNase-seq datasets. Through these examples, the authors show how PISA yields intuitive visualizations that expose motif influence, motif syntax structure, and bias effects that are otherwise masked by profile-aggregated attribution analyses. Overall, the paper presents a well-executed and practically useful interpretability framework, with complementary visualization strategies that offer distinct but consistent perspectives across diverse biological settings.

Concerns:

• Generality of motif-level PISA insights. Core observations such as long-range influence for specific motifs and mixed positive or negative effects are currently illustrated through a small number of striking loci. These examples are intuitive and visually compelling, and additional motif-level summaries across the genome, even in coarse form, would help clarify how representative these behaviors are and how broadly they extend beyond the showcased cases. It would be useful for the discussion to briefly address whether such global summaries are feasible in practice, or whether variability in profile shape and signal structure across loci poses challenges for direct aggregation.

• Quantitative context for MNase bias correction. The PISA-derived synthetic bias track is a conceptually elegant contribution, and its impact is demonstrated primarily through qualitative visualizations and extended data. Including a small set of standardized quantitative metrics in the main text would help readers assess the practical magnitude of the improvement and how it compares to established methods.

• Role of PISA in the sequence design demonstration. The PHO5 promoter design experiment, including experimental validation, is an interesting and well-executed addition. However, this example primarily highlights the capabilities of the predictive model and the genetic algorithm, with PISA playing a more limited role in the design process itself. While PISA can provide mechanistic insight into the designed sequences, similar qualitative interpretations might plausibly be obtained using standard attribution methods. As a result, this application is not the strongest illustration of PISA's distinctive advantages and so I would just be careful with the benefits of PISA for this application.

• Computational considerations. A concise discussion of runtime and scalability of PISA analyses would help readers gauge practical considerations for broader use. In this context, it may be worth noting that while base-resolution attributions are a strength of the method, alternative aggregation choices, such as attributing to short local windows of the predicted profile rather than individual positions, could yield smoother attribution maps and potentially reduce computational cost. This need not be explored in the current work, but could be mentioned as a possible extension or optional variant of the approach.

• Positioning relative to existing attribution methods. PISA is implemented using DeepSHAP and builds directly on Shapley-based attributions for sequence-to-profile models. Clarifying how this formulation relates conceptually to other methods that could give similar insights, such as Jacobian-based approaches, would help situate the method within the broader interpretability literature. It may also be useful to state explicitly that while DeepSHAP is the attribution method explored here, the PISA formulation itself could, in principle, be paired with other first-order attribution schemes.

• Missing Extended Data panel. Extended Data Figure 8e is referenced in the text but does not appear to be present in the current figure set, which currently includes panels a through d.

(Remarks on code availability)
The github codebase is clearly organized, with straightforward installation instructions and well-written ReadTheDocs documentation. Providing a small number of example use cases, such as simple end-to-end workflows or Colab notebooks demonstrating typical analyses, would further lower the barrier to adoption and help new users get started more quickly.

Reviewer #4

(Remarks to the Author)
I co-reviewed this manuscript with one of the reviewers who provided the listed reports. This is part of the Nature Communications initiative to facilitate training in peer review and to provide appropriate recognition for Early Career Researchers who co-review manuscripts.

(Remarks on code availability)
The github codebase is clearly organized, with straightforward installation instructions and well-written ReadTheDocs documentation. Providing a small number of example use cases, such as simple end-to-end workflows or Colab notebooks demonstrating typical analyses, would further lower the barrier to adoption and help new users get started more quickly.

Version 1:

Reviewer comments:

Reviewer #1

(Remarks to the Author)
The authors have satisfactorily addressed my concerns.

(Remarks on code availability)

Reviewer #2

(Remarks to the Author)
The manuscript has been strengthened by the revisions and all reviewer comments have been appropriately addressed.

(Remarks on code availability)
The code is well organized and will be a usable resource for the community.

Reviewer #3

(Remarks to the Author)
The authors have addressed all of my concerns. PISA represents a powerful new method, and the community should be excited about the deeper insights it offers into profile-based sequence-to-function models.

(Remarks on code availability)

Reviewer #4

(Remarks to the Author)
I co-reviewed this manuscript with one of the reviewers who provided the listed reports. This is part of the Nature Communications initiative to facilitate training in peer review and to provide appropriate recognition for Early Career Researchers who co-review manuscripts.

(Remarks on code availability)
The github codebase is clearly organized, with straightforward installation instructions and well-written ReadTheDocs documentation. Providing a small number of example use cases, such as simple end-to-end workflows or Colab notebooks demonstrating typical analyses, would further lower the barrier to adoption and help new users get started more quickly.

Open Access This Peer Review File is licensed under a Creative Commons Attribution 4.0 International License, which permits use, sharing, adaptation, distribution and reproduction in any medium or format, as long as you give appropriate credit to the original author(s) and the source, provide a link to the Creative Commons license, and indicate if changes were made.
In cases where reviewers are anonymous, credit should be given to 'Anonymous Referee' and the source.
The images or other third party material in this Peer Review File are included in the article's Creative Commons license, unless indicated otherwise in a credit line to the material. If material is not included in the article's Creative Commons license and your intended use is not permitted by statutory regulation or exceeds the permitted use, you will need to obtain permission directly from the copyright holder.
To view a copy of this license, visit https://creativecommons.org/licenses/by/4.0/

Comments to reviewers of Positional Interpretation of Cis-Regulatory Code and Nucleosome Organization with Deep Learning Models

Charles E. McAnany[1], Melanie Weilert[1], Grishma Mehta[1,2], Fahad Kamulegeya[1], Jennifer M. Gardner[1], Jacob Schreiber[3,4], Anshul Kundaje[5,6], Julia Zeitlinger[1,7*]

[1*]Stowers Institute for Medical Research, Kansas City, MO, 64110, USA.
[2]Indian Institute of Science Education & Research, Pashan, Pune 411008, India.
[3]Research Institute of Molecular Pathology, Vienna BioCenter, Vienna, 1030, Austria.
[4]Department of Genomics and Computational Biology, UMass Chan Medical School, Worcester, MA, 01655, USA.
[5]Department of Genetics, Stanford University, Palo Alto, CA, 94304, USA.
[6]Department of Computer Science, Stanford University, Palo Alto, CA, 94304, USA.
[7]Department of Pathology & Laboratory Medicine, The University of Kansas Medical Center, Kansas City, KS, 66160, USA.

1 Overview

We appreciate the thoughtful and helpful comments from all three reviewers. Of course everyone says that, but we really mean it. These reviewer comments pointed out great opportunities to strengthen our manuscript and it is clear that all of the reviewers thought deeply about our results. Broadly, our revisions fall into three parts.

First of all, all reviewers felt that it would be helpful to extend our insights obtained from PISA to more examples, and we have now done so as much as we could. We performed PISA on hundreds of motif instances to show that the broad effect of *Oct4-Sox2* and the local effect of *Nanog* are present globally. We show a video of many PISA squid plots of the *Reb1* motif, and we quantified how our identified barriers compare to published Micro-C chromatin domains.

Second, we have significantly expanded our validation of the bias correction strategy for MNase. We now show that the bias model does not learn nucleosome positioning signals around TSSs and that our correction algorithm works with very small amounts of data.

Third, we have expanded our analysis and discussion of the properties of the barriers we identify. We now quantify their overlap with the previously annotated boundaries from Micro-C, and analyze their motif composition. This shows that a large fraction of the barriers is characterized by *polyA* motifs, which have previously been difficult to identify experimentally and computationally, thus underscoring the power of deep learning approaches.

In this response, we will use black text for reviewer comments and *italic text for text taken directly from the manuscript.*

2 Reviewer 1

2.1 Summary

McAnany et al. introduce PISA (Pairwise Influence by Sequence Attribution), an interpretability framework for sequence-to-profile neural networks. By avoiding the aggregation of contribution scores across the output window, PISA aims to resolve the specific spatial range of motif effects. The authors apply this tool to ChIP-nexus/seq, ATAC-seq, and MNase-seq data, introducing a novel method for synthesizing enzymatic bias tracks for MNase-seq by exploiting 5'/3' nucleosome symmetry. Finally, they use the model to define chromatin barrier elements and experimentally validate a genetic algorithm for nucleosome engineering at a single yeast locus. The spatial visualizations and MNase bias-correction logic are innovative, and the authors demonstrate them in intriguing and exciting applications. The manuscript often leans on visual intuition over unassailable quantitative rigor. For the most part, it toes my comfort line; I don't leave the paper overly suspicious of any claims, but I do believe the claims could be strengthened in several places that I comment on below.

2.2 Major Comments

2.2.1 MNase bias scalability

The authors note that calculating PISA genome-wide for S. cerevisiae took "a few days on three Nvidia A100 GPUs" and suggest that for larger genomes, one should train a bias model on a subset of PISA-derived data. To substantiate this recommendation, it would be valuable to see a learning curve analysis: training the bias model on increasingly large subsets of the yeast genome and evaluating its prediction accuracy against the full genome-wide PISA calculation. This would empirically define how much synthetic training data is sufficient and demonstrate the feasibility of this strategy for mammalian genomes.

We have trained our bias model on several subsets of our synthetic bias track and find that a very small amount of training data - as few as 1,500 regions - is indeed sufficient to match the performance of the bias model trained genome-wide. We have added Extended Data Figure 7 showing this result and we mention this finding in the methods section.

2.2.2 Framing of PISA Novelty

The manuscript frames PISA as a "new interpretation tool" that "overcomes the obstacles" of current attribution methods (Introduction).

> However, mathematically, PISA is the application of DeepSHAP to individual output positions without the subsequent aggregation step typically performed in the field. The PISA value P_ij is simply the raw Shapley value matrix, which DeepSHAP inherently calculates. While visualizing this matrix (via squid plots and heatmaps) is a valuable contribution and yields new biological insights, framing it as a novel algorithm or tool distinct from DeepSHAP is misleading. I recommend revising the text to characterize PISA as a visualization framework or analytical strategy that leverages existing attribution scores, rather than a new attribution method itself.

We agree that the PISA algorithm is computationally trivial - the novelty is in its idea and biological application. To avoid this misunderstanding, we have now revised the text, removed the phrase "new interpretation tool" and made sure we highlight that PISA uses DeepSHAP as a backend:

> *Here we overcome these obstacles by introducing a new strategy called pairwise influence by sequence attribution (PISA) to visualize the range and level by which each individual base impacts each genomic coordinate at an individual locus. We implemented PISA using DeepSHAP in a package called BPReveal, which expands the capabilities of BPNet and ChromBPNet to support multiple different data types. (line 108 right)*

And we also clarify that the "current interpretation strategies" we are referring to are the ones used by previous genomics models that aggregate the entire output profile before calculating attribution scores:

> *A major drawback is that current attribution strategies for sequence-to-profile models (such as the counts and profile metrics used in BPNet) rely on reductive representations of how each base impacts an output prediction. Attribution methods typically quantify an input's effects on the entire output window, and thus do not reveal where in the experimental profile a motif exerts its influence and whether the influence is narrow or broad. (line 117 left)*

2.2.3 Chromatin barrier quantification

> The authors claim their model identifies barrier elements that "resemble Micro-C interaction maps". Currently, this claim is supported primarily by visual comparison of the RER2 locus. The analysis would be strengthened by a genome-wide statistical comparison between their predicted "lean" values/barriers and experimental Micro-C boundary scores.

This was a great suggestion for strengthening the manuscript. We have now identified barriers genome-wide and quantified the overlap with published domain boundaries called from Micro-C data. Half of the Micro-C boundaries lie within 87 bp of one of our barriers, showing that they frequently co-occur. We show this in

Extended Data Figure 12(c). Furthermore, both our barriers and the domain boundaries contain binding sites of Rsc3, Abf1, and Reb1. Interestingly, TF binding is a poor predictor, with peaks from experimental binding data overlapping poorly with both our called barriers and the published domain boundaries. The *polyA* motif seems to be enriched around barriers, often with several motif instances nearby. We have added these analyses to the manuscript (Extended Data Figure 12(f)), and have rewritten the text accordingly.

2.2.4 Barrier conceptual circularity

> In Figure 6, the authors define barrier elements using a "lean" metric (resistance to nucleosome shifting) and report these are enriched for Abf1 and Reb1 motifs. This reasoning appears circular. Abf1 and Reb1 are known to create rigid, phased nucleosomes; a model trained on MNase-seq must learn this. Therefore, "discovering" that barriers are Abf1/Reb1 sites effectively reduces to the trivial observation that strong nucleosome positioners resist shifting. To demonstrate that barriers capture novel biology rather than just high-affinity binding, the authors should computationally decouple lean from standard occupancy. A scatter plot comparing Lean Score vs. Predicted Occupancy would clarify if the metric identifies a distinct mechanical property or merely proxies for GRF binding strength. Discordant sites (High Lean/Low Occupancy) would be particularly strong evidence of novelty.

We can see how this analysis appeared circular. It is indeed not surprising that the barriers we have discovered consist of nucleosome-positioning motifs. However, a closer analysis revealed that a majority of the barriers do **not** co-occur with known nucleosome-positioning motifs, but rather co-occur with combinations of weak *polyA* motifs, which are difficult to discover computationally and experimentally. We have rewritten this section to avoid the impression of circularity, added the additional analyses, and emphasized more why our approach of identifying barriers - and thus chromatin domain boundaries - is useful:

> *Like Micro-C boundaries, our barriers are also preferentially found at promoters and are enriched for* Abf1*,* Reb1*,* Rsc3 *and* polyA *motifs [...] We conclude that barriers or domain boundaries are indeed composed of nucleosome-positioning motifs. Unexpectedly though, we found that the majority were either not near a mapped TF motif or not near an experimentally determined ChIP-exo binding peak of Abf1, Reb1 or Rsc3. Instead, barriers or micro-C boundaries are often composed of multiple* polyA *motifs, which cannot be mapped experimentally since no known yeast TF binds them.* *(line 785 right)*

2.3 Minor Comments

2.3.1 Robustness to biological asymmetry

> The synthetic bias derivation assumes nucleosome signals are perfectly symmetric and that a single genome-wide offset (179bp) correctly aligns 5'/3' contributions at all loci. However, local variation in fragment length (e.g. due to partial unwrapping, remodeling, or sequence-dependent MNase digestion kinetics) would cause incomplete cancellation, potentially leaking biological signal into the bias track. While Extended Data Figure 4 suggests the bias model avoids capturing obvious regulatory motifs, this does not rule out the loss of subtler asymmetric features. To rigorously quantify this potential signal loss, I recommend the authors either (1) simulate asymmetric nucleosomes to establish a leakage error margin, or (2) demonstrate that the predicted "bias" magnitude does not systematically correlate with known asymmetric genomic features (e.g., +1 nucleosomes at TSSs). That being said, if the authors determine that these computational validations are out of scope, I would be satisfied with a revision to the Discussion that explicitly acknowledges this limitation.

We agree that the assumed symmetry is a definite limitation of our method. To partially address this concern, we trained a set of new bias models only on regions with low variability in fragment length, thus preventing the bias model from seeing partial nucleosomes. We have combined this analysis with our analysis limiting the amount of training data for the bias model, so the low-data traces in Extended Data Figure 7 correspond to the strictest cutoff for fragment length homogeneity. We saw no difference in the bias model when we selected regions with higher and higher uniformity, suggesting that partial nucleosomes are sufficiently rare in the yeast genome that the bias model did not learn them.

2.3.2 Offset sensitivity

> The synthetic bias derivation relies on a 179bp offset determined by maximizing agreement between shifted 5'/3' PISA matrices. How sensitive is the resulting bias track to this parameter? A brief sensitivity analysis showing that results are stable across a reasonable range (e.g., ±10bp) would increase confidence in the approach's robustness.

We have added Extended Data Figure 14, showing predictions and BAT plots of the bias-corrected model at offsets from -10 bp to +10 bp. We find that the bias model does start to learn about the TSS when the bias is off by 5 bp or more, and this also corresponds to the point where a bias spike begins to appear in the BAT plot. For every offset, the residual model still matches the nucleosome profile around the TSS very well, showing that only a small amount of biological information was able to leak into the bias model even with offsets as large as 10 bp.

2.3.3 Experimental validation

The use of a Genetic Algorithm to engineer nucleosome positioning is a compelling proof-of-concept. However, validation is restricted to a single successful edit at the PHO5 promoter. While the Introduction frames this as a "proof-of-principle," the Results suggest broader capability. Could the authors clarify if other designs were attempted experimentally? Understanding the hit rate would be insightful. If further validation is not feasible, I recommend adding Discussion text explicitly framing this as an N=1 demonstration and outlining potential failure modes for broader application.

We have now created three mutants using our genetic algorithm and added them in Extended Data Figure 13 and we describe them in the main text. (We designed a fourth mutant but our CRISPR attempts were unsuccessful.)

We also performed MNase-seq on two other designed mutants. One of these agreed with our predictions, showing a shifted nucleosome to the left of the high-affinity site, whereas in the other, the model mis-predicted a stable nucleosome at the same position. (line 880 right)

2.3.4 BPReveal bias transformation

In Methods Section 4.3, the authors introduce a more expressive, nonlinear transformation for the bias model. Extended Data Table 2 indicates that this technique improves performance on both profile prediction (lower JSD) and total read count prediction (higher Spearman correlation) compared to the standard linear approach. Given this consistent improvement, do the authors recommend that this non-linear transformation become the new standard for all future BPNet/ChromBPNet applications, or are there specific contexts where the simpler linear transformation is still preferred?

The nonlinear transformation model provides a small improvement but may not be worth the effort for most cases and is not our recommendation for ATAC-seq. We now mention this in the methods:

ChromBPNet, by comparison, does not transform the logits from the bias model, and applies a pre-computed scaling factor to the counts output. (We find that this nonlinear transformation does not offer a significant improvement over ChromBPNet for ATAC-seq data.) (line 1193 right)

2.3.5 tangermeme

Line 123 says tangermeme is "a PyTorch-based version of BPNet", but its introducing manuscript describes it much differently.

We have updated the text to clarify that tangermeme is a suite of tools and that PISA is just one of the tools it contains.

> *As an example, we have implemented* PISA *into tangermeme, a PyTorch-based suite of tools for* BPNet*-like models[1].* ***(line 131 right)***

2.4 Remarks on code availability

> The results appear reproducible.

3 Reviewer 2

3.1 Summary

This manuscript presents an innovative approach to attributions for sequence-to-function neural networks. PISA addresses several limitations of existing attribution methods by quantifying the impact of input sequence positions at each position in the output. This approach has several advantages, notably the ability to identify both positive and negative effects of individual motifs on model predictions. The higher-resolution attributions of PISA also facilitate identifying biases in experimental assays such as MNase-seq. The authors demonstrate that PISA can generate useful interpretations of neural networks, eliminate biases from MNase-seq for improved interpretability, and guide the design of experiments to change nucleosome positioning in vivo. The ability to attribute positive and negative effects to motifs proves especially useful for examining the impact of motifs on nucleosome positioning. The software comes as a useful general-purpose package with high quality visualization tools and will thus be useful to the field.

3.2 Comments

3.2.1 More extensive validation

PISA's ability to deconvolve attributions between individual input and output positions is powerful and the manuscript compellingly demonstrates several important applications of the approach. But the validation of the individual motif effects revealed by the attributions is limited. Most of the vignettes show the results of PISA attributions on a single sequence in one condition. Experimental validation of attributions at scale is not feasible, but it would increase confidence in the method if the performance of the attributions were tested more extensively on some examples of motif or transcription factor perturbations, taken from published data. For example, there are some Zelda-bound loci that remain accessible in embryos lacking Zelda (PMID 26335634). Can PISA distinguish the effects of the Zelda motif at sites where Zelda is required for accessibility, from Zelda motifs that remain accessible in the absence of Zelda? One study examined NANOG binding when OCT4 was knocked down (PMID 34143975). Can sites bound by NANOG in the absence of OCT4 be identified by PISA (as sites where a nearby OCT4/SOX2 motif has no attribution connection to the NANOG motif)? Such comparisons wouldn't necessarily require training new models. Published data could be used to identify Zelda-dependent vs Zelda-independent accessible sites, which could then be compared in the already-trained model.

[editorial note: unpublished, confidential data redacted]

We thank the reviewer for the suggestion and have now added more validations of our insights by scaling to hundreds of examples across the genome. For example, we have added Extended Data Figure 3, illustrating a large-scale PISA analysis to show that the pioneering effect of *Oct4-Sox2* and the local effect of *Nanog* are observed globally.

We have also carefully analyzed why some Zelda-bound regions remain accessible in the absence of Zelda. We downloaded the Zelda ChIP-seq data used in that publication[2] and analyzed how they relate to the accessibility changes caused by Zelda RNAi [3]. Strikingly, in regions where our model has not identified any *Zelda* motifs, the ChIP-seq data and RNAi data are effectively uncorrelated, whereas the correlation is significant in regions with more *Zelda* motifs (Reviewer Figure 1). This suggests that the "false positives" are due to nonspecific binding in regions that lack *Zelda* motifs. However, these insights showcase the model accuracy and not PISA, which is why we have not included these analyses in the manuscript. Instead, we decided to showcase different motif shapes by the different range of effects of the *Oct4-Sox2* motif versus the *Nanog* motif in Extended Data Figure 3, and we have also added an Extended Data video showing PISA squid plots of many *Reb1* motifs to illustrate how endogenous motifs lean.

For the reviewer's interest, we explored the *Klf4* locus in figure 4(c) of PMID 34143975 and identified a weak *Oct4* motif that did not contribute to Nanog binding

[editorial note: unpublished, confidential data redacted]

as well as a strong *Sox2* motif that does contribute to Nanog binding (Reviewer Figure 2).

3.2.2 Clarity of bias correction

The initial discussion of bias correction on p. 9 is difficult to follow. The second paragraph mentions BPReveal models trained to predict Drosophila embryo ATAC-seq data. I'm assuming these models were trained using the ChromBPNet strategy, but this isn't stated explicitly.

> The paragraph mentions a 'combined model'. Is this just a model without the bias removed? Is the main point of this section (discussing Fig. 4a,b) to demonstrate that PISA can visualize bias effects?

We agree and have now rewritten the text extensively. We now explain the ATAC-seq bias removal strategy (à la ChromBPNet) when we first introduce the Drosophila ATAC-seq model. In the bias detection section, we now distinguish when we are considering the combined (uncorrected) model, the bias model, and the residual model. We also make clear that PISA is not just used to detect the experimental biases, but also plays a central role in constructing the bias track for MNase-seq.

3.2.3 Reproducibility

> How reproducible are the new MNase-seq experiments? A measure of reproducibility should be included in the Methods.

We have added Extended Data Table 5, showing the reproducibility of our experimental results. We compare the two replicates of each mutant, and compare the pooled results between all pairs of mutants. All replicate-replicate comparisons have a Pearson correlation of at least 0.75, and all comparisons between pooled samples have Pearson correlation values of at least 0.9.

3.3 Remarks on code availability

> The code is well organized, well documented, and is broadly applicable. It will be a useful resource for the community.

4 Reviewer 3

4.1 Summary

This manuscript introduces PISA, an interpretation method for sequence-to-profile models that assigns nucleotide contribution scores to each position of the predicted output profile. By retaining this two-dimensional attribution structure, PISA enables spatial analysis of nucleotide influence along the predicted output signal, providing higher resolution than standard attribution approaches based on profile summary statistics. The method is implemented within the BPReveal framework and applied across transcription factor binding, chromatin accessibility, and MNase-seq datasets. Through these examples, the authors show how PISA yields intuitive visualizations that expose motif influence, motif syntax structure, and bias effects that are otherwise masked by profile-aggregated attribution analyses. Overall, the paper presents a well-executed and practically useful interpretability framework, with complementary visualization strategies that offer distinct but consistent perspectives across diverse biological settings.

4.2 Concerns

4.2.1 Generality of motif-level PISA insights

Core observations such as long-range influence for specific motifs and mixed positive or negative effects are currently illustrated through a small number of striking loci. These examples are intuitive and visually compelling, and additional motif-level summaries across the genome, even in coarse form, would help clarify how representative these behaviors are and how broadly they extend beyond the showcased cases. It would be useful for the discussion to briefly address whether such global summaries are feasible in practice, or whether variability in profile shape and signal structure across loci poses challenges for direct aggregation.

We have now expanded some of the insights obtained from PISA to more examples. First, we have measured the shapes of the effects of hundreds of *Oct4-Sox2* and *Nanog* motifs and we see that the broad nature of *Oct4-Sox2* and narrow nature of *Nanog* are reproduced globally. This result is included as Extended Data Figure 3. Second, we have calculated asymmetry values (distinct from lean values) for every *Reb1* motif identified by our MNase-seq model (limited to our validation and test chromosomes). To illustrate the range of asymmetry in endogenous motifs, we have included a supplemental video showing the PISA plots for all of these motifs, sorted by their asymmetry value. More broadly, though, we do not currently have a general framework for aggregating PISA data beyond averaging, and we have added this limitation to our discussion:

[W]e do not currently have a mathematical approach to extract general rules from PISA data. For example, PISA plots make it clear that our models have learned aspects of cooperativity, but we lack a formalism that would allow us to directly extract the relationships between motifs. *(line 972 right)*

4.2.2 Quantitative context for MNase bias correction

The PISA-derived synthetic bias track is a conceptually elegant contribution, and its impact is demonstrated primarily through qualitative visualizations and extended data. Including a small set of standardized quantitative metrics in the main text would help readers assess the practical magnitude of the improvement and how it compares to established methods.

We have added quantifications to the BAT plots legends showing the bias correction effectiveness in Extended Data Figure 8. In brief, using naked DNA to generate a synthetic bias is hardly better than no bias correction at all. SeqOutBias causes a considerable reduction in bias, which we now mention in the text since it could be used as an alternative to our bias correction approach:

To benchmark the bias removal, we compared our results to those of other MNase bias removal methods. We trained a bias model on MNase-seq data obtained from naked DNA[4, 5] or used statistical modeling such as seqOutBias[6] before training on the MNase-seq data. BAT plots show that these methods reduced the bias signal, and quite considerably in the case of the seqOutBias-corrected data, but both methods left more uncorrected enzymatic bias than our corrected prediction. Our PISA-derived bias track has the strongest reduction in bias. *(line 589 left)*

4.2.3 Role of PISA in the sequence design demonstration

The PHO5 promoter design experiment, including experimental validation, is an interesting and well-executed addition. However, this example primarily highlights the capabilities of the predictive model and the genetic algorithm, with PISA playing a more limited role in the design process itself. While PISA can provide mechanistic insight into the designed sequences, similar qualitative interpretations might plausibly be obtained using standard attribution methods. As a result, this application is not the strongest illustration of PISA's distinctive advantages and so I would just be careful with the benefits of PISA for this application.

We agree that the design of the *Pho5* mutant is not a direct application of PISA, but rather represents a general experimental validation of the bias-minimized MNase-seq model that was enabled through PISA. We have rewritten this section to make clear that the purpose is to validate the model rather than PISA itself:

> *Having trained a bias-minimized nucleosome model with the help of PISA, we tested whether the model was accurate enough to design sequences with altered nucleosome configurations. [...] Taken together, this highlights the use of PISA in creating synthetic bias tracks for MNase-seq data, enabling bias-minimized nucleosome models for unprecedented interpretation and sequence designs.* (line 860 right)

4.2.4 Computational considerations

> A concise discussion of runtime and scalability of PISA analyses would help readers gauge practical considerations for broader use. In this context, it may be worth noting that while base-resolution attributions are a strength of the method, alternative aggregation choices, such as attributing to short local windows of the predicted profile rather than individual positions, could yield smoother attribution maps and potentially reduce computational cost. This need not be explored in the current work, but could be mentioned as a possible extension or optional variant of the approach.

We have tried to make BPReveal practical to use on commodity hardware, and we have added Extended Data Table 6 to illustrate typical times for model training and interpretation. We also note that ISM is implemented as an optional interpretation backend in BPReveal, though it is much slower than DeepSHAP.

4.2.5 Positioning relative to existing attribution methods

> PISA is implemented using DeepSHAP and builds directly on Shapley-based attributions for sequence-to-profile models. Clarifying how this formulation relates conceptually to other methods that could give similar insights, such as Jacobian-based approaches, would help situate the method within the broader interpretability literature. It may also be useful to state explicitly that while DeepSHAP is the attribution method explored here, the PISA formulation itself could, in principle, be paired with other first-order attribution schemes.

We agree that PISA-style visualization could be used with a variety of attribution approaches, and we now mention this in the main text. We have added Extended Data Figure 1 illustrating PISA maps derived from directly calculating a Jacobian (i.e., input * gradient) and by performing saturation mutagenesis. Directly calculating a derivative is messy, but saturation mutagenesis is able to identify motifs effectively. Interestingly, an ISM-based PISA assigns very little importance to the DNA forming the core of a nucleosome and instead focuses almost entirely on motif effects. We

speculate that this is due to the distributed and redundant nature of the intrinsic signals for nucleosome positioning.

4.2.6 Missing Extended Data panel

> Extended Data Figure 8e is referenced in the text but does not appear to be present in the current figure set, which currently includes panels a through d.

Oops! Thank you for the catch.

4.3 Remarks on code availability

> The github codebase is clearly organized, with straightforward installation instructions and well-written ReadTheDocs documentation. Providing a small number of example use cases, such as simple end-to-end workflows or Colab notebooks demonstrating typical analyses, would further lower the barrier to adoption and help new users get started more quickly.

References

[1] Schreiber, J. tangermeme: A toolkit for understanding cis-regulatory logic using deep learning models. *BioRxiv* (2025). URL http://biorxiv.org/lookup/doi/10.1101/2025.08.08.669296.

[2] Harrison, M. M., Li, X.-Y., Kaplan, T., Botchan, M. R. & Eisen, M. B. Zelda binding in the early drosophila melanogaster embryo marks regions subsequently activated at the maternal-to-zygotic transition. *PLOS Genetics* **7**, 1–13 (2011). URL https://doi.org/10.1371/journal.pgen.1002266.

[3] Brennan, K. J. *et al.* Chromatin accessibility in the *Drosophila* embryo is determined by transcription factor pioneering and enhancer activation. *Developmental Cell* **58**, 1898–1916.e9 (2023). URL https://linkinghub.elsevier.com/retrieve/pii/S1534580723003477.

[4] Begley, V. *et al.* Xrn1 influence on gene transcription results from the combination of general effects on elongating RNA pol II and gene-specific chromatin configuration. *RNA Biology* **18**, 1310–1323 (2021). URL http://dx.doi.org/10.1080/15476286.2020.1845504.

[5] Gutiérrez, G. *et al.* Subtracting the sequence bias from partially digested MNase-seq data reveals a general contribution of TFIIS to nucleosome positioning. *Epigenetics & Chromatin* **10**, 58 (2017). URL http://dx.doi.org/10.1186/s13072-017-0165-x.

[6] Martins, A. L., Walavalkar, N. M., Anderson, W. D., Zang, C. & Guertin, M. J. Universal correction of enzymatic sequence bias reveals molecular signatures of protein/DNA interactions. *Nucleic Acids Research* **46**, e9 (2018). URL http://dx.doi.org/10.1093/nar/gkx1053.
